# Decoupled systems on trial: Eliminating bottlenecks to improve aquaponic processes

**DOI:** 10.1371/journal.pone.0183056

**Published:** 2017-09-28

**Authors:** Hendrik Monsees, Werner Kloas, Sven Wuertz

**Affiliations:** 1 Leibniz-Institute of Freshwater Biology and Inland Fisheries, Berlin, Germany; 2 Albrecht Daniel Thaer-Institute of Agricultural and Horticultural Sciences, Humboldt University Berlin, Berlin, Germany; University of Delhi, INDIA

## Abstract

In classical aquaponics (coupled aquaponic systems, 1-loop systems) the production of fish in recirculating aquaculture systems (RAS) and plants in hydroponics are combined in a single loop, entailing systemic compromises on the optimal production parameters (e.g. pH). Recently presented decoupled aquaponics (2-loop systems) have been awarded for eliminating major bottlenecks. In a pilot study, production in an innovative decoupled aquaponic system was compared with a coupled system and, as a control, a conventional RAS, assessing growth parameters of fish (FCR, SGR) and plants over an experimental period of 5 months. Soluble nutrients (NO_3_^-^-N, NO_2_^-^-N, NH_4_^+^-N, PO_4_^3-^, K^+^, Ca^2+^, Mg^2+^, SO_4_^2-^, Cl^2-^ and Fe^2+^), elemental composition of plants, fish and sludge (N, P, K, Ca, Mg, Na, C), abiotic factors (temperature, pH, oxygen, and conductivity), fertilizer and water consumption were determined. Fruit yield was 36% higher in decoupled aquaponics and pH and fertilizer management was more effective, whereas fish production was comparable in both systems. The results of this pilot study clearly illustrate the main advantages of decoupled, two-loop aquaponics and demonstrate how bottlenecks commonly encountered in coupled aquaponics can be managed to promote application in aquaculture.

## Introduction

Aquaponic systems have been presented as a sustainable and resource friendly development of common recirculating aquaculture systems (RAS). Here, accumulated nutrients and water of RAS are recycled by an integrated hydroponic (soilless) plant production unit [1]. Nevertheless major drawbacks became obvious in comparison to both, professional aquaculture as well as hydroponic plant production.

Classical aquaponic systems, commonly referred to as coupled or 1-loop aquaponic systems, were described already more than 30 years ago [2, 3]. Here, the aquaculture unit and the hydroponic unit are arranged in a single loop where process water is directed from the aquaculture to the hydroponic unit and back. Inevitably, such systems provide the same water quality for both, fish and plants, which necessarily represent a compromise in the rearing conditions for each production line. Probably, the need to compromise and the lack of control on the production are the key obstacles why commercial applications are scarce and the majority of aquaponic systems are small-scale units, patronizingly called "backyard aquaponics", in schools for education purposes or in research facilities [4].

Current efforts aim at decoupled systems arranged in separate loops where process water is mainly recirculated within the respective unit, thereby allowing a better control of the species-specific requirements [5, 6]. Here, water is recirculated within the respective unit (RAS or hydroponics) and water loss due to evapotranspiration of the plants is compensated on-demand, directing process water from the fish tanks via a one-way valve into the hydroponic reservoir. Thus, water from the hydroponic unit is not redirected into the fish tanks and conditions within the hydroponic unit can be managed separately, if necessary. To further improve water efficiency, [5] described a greenhouse production equipped with an additional air conditioning system with an integrated cold trap to condensate water that is evapotranspirated by plants as well as from the RAS, redirecting the condensate (pure water) to the RAS unit.

A high diversity of fish species has been produced in aquaponics, among them catfish, carp perch, sea bass and, most prominently, tilapia [2, 7–10]. The number of established crop plants may even be higher, including strawberries, tomatoes, basil and lettuce [5, 11, 12]. Here tomatoes are considered as more difficult to grow, since nutrients, especially potassium, are required in big quantities [13]. Additionally, tomatoes are among the most important vegetables worldwide both in economical terms and in consumption [14].

In principle, the most important nutrients derived from the fish rearing and subsequently utilized by the growing plant crops are nitrogen (N), phosphorus (P) and potassium (K). Among them, dissolved nitrogen is primarily considered for balancing fish and plant production during system design. Ideally, fish provide the nitrogen to sustain the plant crop growth without the need for additional nitrogen fertilization. Most of this nitrogen originates from the protein metabolism of the fish and is excreted via the gills as ammonia [15]. Due to the high toxicity of ammonia, biofilters (moving bed, trickling filter) are integrated in the fish unit to support microbial nitrification, converting ammonia to nitrate. For optimal operation this reaction requires a pH ≥ 7 [16]. Since the process of nitrification results in the release of protons during ammonia oxidation [17], RAS operators have to counteract the decrease in pH by the addition of e.g. limestone [5]. On the other hand, during plant production, most nutrients become available at a pH of 5.5–6.5 [18]. Thus, in commercial hydroponic production, pH is controlled by the addition of acids, e.g. nitric acid [19]. Consequently, in coupled aquaponics compromises have to be taken with regard to the production parameters including a commonly reported pH 7 [9]. Obviously, this is not ideal for neither fish nor plants and species-specific adjustment by a decoupling of both units is desirable. Also, from an animal welfare perspective, addition of fertilizers in situations of nutrient imbalances is controversial due to the fact that fish are intentionally confronted with suboptimal or even negative rearing conditions. Recently, concepts for decoupled systems have been presented [1, 5]. Still, direct comparison of decoupled and coupled systems is lacking.

To our knowledge this is the first study comparing coupled and decoupled aquaponics under realistic production conditions. The results of this pilot study demonstrate the main advantages of decoupled aquaponics and highlight the bottlenecks of classical aquaponic systems. Furthermore, practical and theoretical recommendations should serve as guidance for future system design and best practices.

## Material and methods

### Aquaponic system

Experiments were conducted at the aquaponic research facility of the Leibniz-Institute of Freshwater Ecology and Inland Fisheries (Berlin, Germany). Briefly, three identical RAS with a total volume of 16.5 m^3^ each (culture volume 6.8 m^3^, four separate rearing tanks of 1.7 m^3^ each) were stocked with Nile tilapia (*Oreochromis niloticus*, weight: Ø 68 g) and purchased at a commercial supplier (Kirschauer Aquakulturen, Germany).

**Fig 1:**
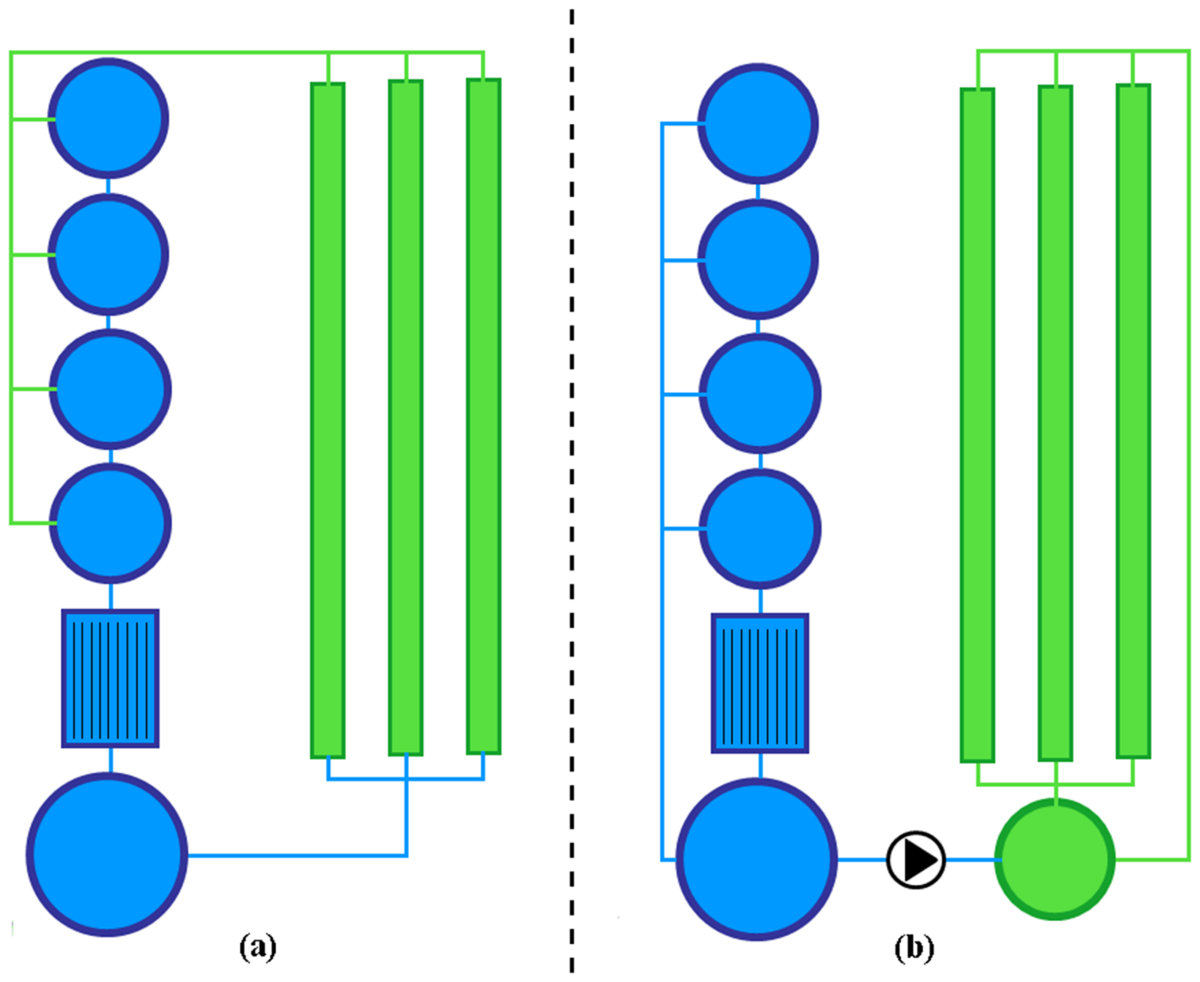
Schematic illustration of classical (coupled) and decoupled aquaponics. (a): Classical aquaponic system consisting of a RAS (blue: rearing tanks, clarifier and biofilter) directly connected to the hydroponic unit (green: NFT-trays). Water is constantly circulated from RAS to hydroponic and back to RAS. (b): Decoupled aquaponic system consisting of a RAS connected to the hydroponic unit (with additional reservoir) via one-way-valve. Water is separately recirculated in each system and water is just supplied on-demand from RAS to the hydroponic unit, but not back.

The study was carried out in compliance with the German legislation as authorized by the Regional Office for Health and Social Affairs Berlin (permit #: ZH 114). For biofiltration (nitrification) each RAS was equipped with a moving bed filter (2 m^3^) providing a substrate surface of approximately 1350 m^2^. In the first RAS (A) a drum filter (mesh size: 100μm) was used to remove suspended solids, representing the most frequently used technology used in commercial RAS. Here, no hydroponic unit was integrated and this system was used as control (conventional aquaculture reference). In the two remaining, coupled (RAS C) and decoupled (RAS D) systems (Fig 1), suspended solid removal was achieved with a clarifier (1.5 m^3^), which is often used in aquaponic applications due to the energy and water efficiency.

Here, five NFT-trays (l45 cm * 30 cm, h: 28 cm each) were arranged as hydroponic unit, integrated to the RAS (C, D). RAS D was connected to the hydroponic units via one-way-valve, providing a decoupled, two-loop aquaponic system [5]. As a consequence, water from RAS D was only directed on demand to the respective hydroponic unit, but not redirected to the RAS. RAS C was operated as a single-loop aquaponic system (coupled, classical approach) where five hydroponic units were connected to the RAS with a by-pass using a pump (10L/min) installed in the pump sump. To prevent clogging and fouling of the plant roots by suspended solids originating from the RAS, a small filter (Eheim, Germany) was interposed and cleaned on a regular basis. Over the experimental period, fish were fed a commercial food (Aller Float 37/10 2 mm and 3 mm, Emsland-Aller Aqua, Germany). Temperature, pH and oxygen were determined daily (HQ40d multi, Hach Lange GmbH, Germany); pH was regulated with Ca(OH)_2_. Selected nutrients (NO_3_^-^-N, NO_2_^-^-N, TAN, PO_4_^3-^, K^+^, Mg^2+^, Ca^2+^, SO_4_^2-^, Cl^-^ and Fe^2+^) in the water were determined spectrophotometrically (DR3900 Hach Lange, Berlin, Germany) with the respective kit.

### Tomato plants

Tomato plants (*Solanum lycopersicum*, variety: Pannovy) originated from a company specialized on hydroponic vegetables (Schwanteland GmbH, Germany). They were grown in rock wool cubes (10 cm * 10 cm) and had a mean height of 42.1 cm (± 4.3 cm). Per RAS, 15 tomato plants were randomly distributed to the trays of the respective hydroponic unit. Water consumption and fertilizer supply was according to Table 1. The fertilizers had the following composition: Krista K Plus (Yara, Germany): 13.7% total N (13.7% NO_3_-N) and 46.3% K_2_O; CalciNit (Yara, Germany): 15.5% total N (14.4% NO_3_-N and 1.1% NH_4_-N) and 26.3% calcium oxide (CaO). Manna Lin M Spezial is a NPK fertilizer with 18% total N (11% NO_3_-N and 7% NH_4_-N), 12% P_2_O_5_, 18% K_2_O, 2% MgO and trace elements including Fe, Mn, Zn, B, Cu, and Mo. Partly KHCO_3_ was also used to increase the potassium concentration.

**Table 1:** Plant growth (fresh weight of fruit, leave, root, stem), fertilizer supplementation and water consumption in the hydroponic unit of the coupled (Hydro C) and decoupled (Hydro D) aquaponic system after 30, 63, 94, 122 and 154 d. Water consumption is only indicated for Hydro D, since Hydro C is coupled to the RAS C and is only given for the entire system (Table 2). Roots and stems were only sampled at the end of the experiments and fresh weight therefore not determined (n.d.) earlier.

Hydroponic	sampling intervals	days [d]	harvest [kg]				fertilizer [g]				water consumption [L]
			fruit	leave	root	stem	Krista K +	Calcinit	Manna Lin M Spezial	KHCO_3_	
**C**	07.04.-06.05.15	30	0.24	11.1	n.d.	n.d.	325	130	60	0	bypass
	07.05.-08.06.15	63	25.90	12.4	n.d.	n.d.	179	140	65	0	bypass
	09.06.-09.07.15	94	13.67	12.7	n.d.	n.d.	160	0	50	300	bypass
	10.07.-06.08.15	122	11.41	6.4	n.d.	n.d.	30	0	0	0	bypass
	07.08.-07.09.15	154	39.66	21.1	5.8	25.7	0	0	0	0	bypass
	**total**	154	**90.9**	**63.7**	**5.8**	**25.7**	**694**	**270**	**175**	**300**	bypass
**D**	07.04.-06.05.15	30	1.6	11.7	n.d.	n.d.	325	130	60	0	634
	07.05.-08.06.15	63	41.2	11.2	n.d.	n.d.	179	140	65	0	990
	09.06.-09.07.15	94	27.2	7.4	n.d.	n.d.	160	0	50	300	964
	10.07.-06.08.15	122	18.6	6.0	n.d.	n.d.	30	0	0	0	983
	07.08.-07.09.15	154	34.9	11.7	2.3	17.1	0	0	0	0	670
	**total**	**154**	**123.5**	**48.0**	**2.3**	**17.1**	**694**	**270**	**175**	**300**	**4961**

### Elemental analysis

Over the five month experimental period, samples of leaves and tomatoes were sampled at four time points (in May, July, August and September). Plants were chosen randomly, per sampling point and system five replicates of two leaves were taken (always the fifth fully developed leave) as well as five replicates of two fully ripe tomatoes. Samples were freeze dried prior to elemental analysis. Total phosphorus (TP), magnesium (Mg), calcium (Ca), potassium (K), sodium (Na) were determined by ICP-OES (inductively coupled plasma optical emission spectrometry; iCAB 6000, Thermo Fisher Scientific Inc., USA) after wet digestion (HCl 37%, HNO_3_ 65%, volumetric ratio 1:3) in a high pressure microwave oven (Gigatherm, Switzerland). C/N analysis of plants and fish were performed using freeze dried (to a constant weight), weighed samples and analyzed in a Vario EL system (Elementar Analysensysteme GmbH, Germany). Composition of sludge (n = 4) and fish (n = 4) was determined accordingly.

### Determination of total solids (TS) and total suspended solids (TSS) in the RAS

For the evaluation of the weekly loss of TS due to cleaning of the clarifier (RAS C and D), water-sludge mixture from the clarifier (1.5 m^3^) was collected three times within the experimental period in a 2 m^3^ tank and homogenized with a pump. Per sampling five subsamples were taken in 10 L containers each. Aliquots of fresh sludge (n = 15) were freeze dried to determine the dry weight: wet weight ratio.

For TSS, water samples (100 ml) were taken in triplicate at the inflow of a fish tank at the beginning of the experiment, after 3 months and at the end of the experimental period. Briefly, samples were filtered through pre-weighted 0.45 μm CA membrane filters (GE Healthcare, United Kingdom), freeze dried to a constant weight and weighed.

### Estimated fate of nitrogen

The schematic illustration of the fate of nitrogen was developed according to the results of the present study and literature values. Literature values considered were those for % N of proteins [20, 21], the excretion of N [22–24], nitrification [16], uncontrolled denitrification [25] and nitrate uptake of tomatoes [26, 27].

## Results

### Plant growth, fertilizer supplementation and water consumption

Plant growth, fertilizer supplemented and water consumption in the hydroponic units of the coupled and decoupled aquaponic system (Hydro C, Hydro D) are presented in Table 1. Over the entire experimental period of 154 d, more tomatoes were harvested from Hydro D (123.5 kg) than from Hydro C (90.9 kg), corresponding to a 36% higher tomato yield in the decoupled system. In contrast, 31% more leaves (63.7 kg), 60% more roots (5.8 kg) and 50% more stem biomass (5.8 kg) were harvested from the coupled system. At the same time, fertilizer supplementation was identical in both systems (Table 1). Water consumption was lowest in the beginning and at the end of the experiment with 1.4 L per plant per day in Hydro D. Between the 07.05 and the 06.08.2015, water consumption was highest and ranged between 2.0 and 2.4 L per plant per day.

### Fish growth and RAS performance

Fish growth, feed conversion ratios (FCR) and specific growth rates (SGR) are presented in Table 2 and were in the same range among all three RAS (A, C, D) over the entire experimental period. The average FCR in each system ranged between 1.2 and 1.3, increasing over time from 1.0 to 1.6, identifying an increased feed conversion in larger fish. In each system, the average SGR was 1.0 whereas a continuous decrease down to 0.5 (A and D) and 0.6 (C) was observed towards the end of the experiment. Water consumption was also comparable between the aquaponic systems. Still, in the aquaculture control RAS (A) the water consumption was higher at 5–6% RAS d^-1^. Also, in both aquaponic systems, addition of limestone was similar and increased from 0.5 kg to 6.1 kg within the experimental period. Approximately 22% less limestone was used in the aquaculture control RAS A to regulate the pH to comparable levels. Initial, final weight and subsequently overall weight gain revealed no difference (<2%) between fish units. Over the entire period mortalities (< 1%) were very low in all systems.

**Table 2:** Details on the stocking, amount of feed fed, specific growth rate (SGR), food conversion ratio (FCR), mortalities, water consumption and limestone added to control pH in the fish units of the coupled (RAS C) and decoupled aquaponic system (RAS D) compared to the control (RAS A) after 30, 63, 94, 122 and 154 d.

RAS	sampling intervals	days [d]	stocked tanks [n]	tank volume [m^3^]	RAS stocking start [kg]	RAS stocking end [kg]	fish growth [kg month^-1^]	feed [kg]	FCR	SGR	mortalities [%] of stocking end	water consumption [m³]	water consumption [%RAS d^-1^]	limestone addition [kg]
**A**	07.04.-06.05.15	30	1	1.7	66.9	104.3	37.4	37.2	1.0	1.5	0.1	14.92	3.0	0.9
	07.05.-08.06.15	63	2	1.7	104.3	153.1	48.8	58.2	1.2	1.2	0.2	29.33	5.4	1.3
	09.06.-09.07.15	94	2	1.7	153.1	220.5	67.4	75.6	1.1	1.2	0.3	29.33	5.7	2.4
	10.07.-06.08.15	122	3	1.7	220.5	273.4	52.9	71.0	1.3	0.8	0.1	28.51	6.2	2.4
	07.08.-07.09.15	154	3	1.7	273.4	324.6	51.3	83.7	1.6	0.5	0.4	32.02	6.1	5.1
	**total / *average***	**154**					**257.7**	**325.6**	***1*.*3***	***1*.*0***	**0.8**	**134.12**	***5*.*3***	**12.1**
**C**	07.04.-06.05.15	30	1	1.7	66.8	101.0	34.2	37.2	1.1	1.4	0.0	15.24	3.1	0.7
	07.05.-08.06.15	63	2	1.7	101.0	147.6	46.6	58.2	1.2	1.1	0.4	13.05	2.4	1.7
	09.06.-09.07.15	94	2	1.7	147.6	218.9	71.3	75.6	1.1	1.3	0.0	13.86	2.7	4.1
	10.07.-06.08.15	122	3	1.7	218.9	275.1	56.2	71.0	1.3	0.8	0.3	16.47	3.6	3.1
	07.08.-07.09.15	154	3	1.7	275.1	330.2	55.1	83.7	1.5	0.6	0.3	15.25	2.9	6.0
	**total / *average***	**154**					**263.4**	**325.6**	***1*.*2***	***1*.*0***	**0.7**	**73.87**	***2*.*9***	**15.6**
**D**	07.04.-06.05.15	30	1	1.7	66.8	102.4	35.6	37.2	1.0	1.4	0.2	15.91	3.2	0.5
	07.05.-08.06.15	63	2	1.7	102.4	145.5	43.2	58.2	1.3	1.1	0.1	12.52	2.3	1.4
	09.06.-09.07.15	94	2	1.7	145.5	217.9	72.3	75.6	1.0	1.3	0.1	13.86	2.7	4.0
	10.07.-06.08.15	122	3	1.7	217.9	271.5	53.7	71.0	1.3	0.8	0.2	16.07	3.5	3.3
	07.08.-07.09.15	154	3	1.7	271.5	323.7	52.2	83.7	1.6	0.5	0.1	13.59	2.6	6.1
	**total / *average***	**154**					**256.9**	**325.6**	***1*.*3***	***1*.*0***	**0.4**	**71.95**	***2*.*8***	**15.3**

### Rearing conditions in the fish and the hydroponic units

Rearing conditions are presented in Table 3. The dissolved oxygen concentration was high (6.3–6.5 mg L^-1^) and within the same range between RAS A, C and D. Over the experimental period a higher average oxygen concentration was recorded in Hydro D (8.2 mg L^-1^) compared to Hydro C (6.5 mg L^-1^) and all fish units. Similarly, the pH was in the same range between fish units RAS A, RAS C / Hydro C and RAS D (pH 7.1–7.4), but substantially lower in the decoupled Hydro D (pH 6.4). The average temperature in all three RAS and Hydro C oscillated around 27°C. In Hydro D a lower average temperature (24.3°C ± 1.7) was observed. The conductivity ranged between 1.1 mS cm^-1^ and 1.5 mS cm^-1^ in the three RAS and Hydro C, but was two fold increased at 3.2 mS cm^-1^ in Hydro D compared to Hydro C (1.5 mS cm^-1^).

**Table 3:** Rearing conditions (dissolved oxygen (O_2_), pH, temperature and conductivity) in the fish (RAS) and hydroponic (Hydro) units of a conventional aquaculture reference (A), a coupled (C) and a decoupled (D) aquaponic system, assessed over the experimental period of 154 days (07.04–07.09.2015).

experimental system	experimental period	days [d]	O_2_[mgL^-1^]	pH	temperature [°C]	conductivity [mScm^-1^]
RAS A	07.04.-07.09.15	154	6.4 (± 1.0)	7.3 (± 0.3)	26.8 (± 1.5)	1.1 (± 0.1)
RAS C / Hydro C	07.04.-07.09.15	154	6.5 (± 1.1)	7.1 (± 0.3)	26.8 (± 1.0)	1.5 (± 0.3)
RAS D	07.04.-07.09.15	154	6.3 (± 1.1)	7.2 (± 0.3)	27.3 (± 1.2)	1.5 (± 0.3)
Hydro D	07.04.-07.09.15	154	8.2 (± 0.4)	6.4 (± 0.7)	24.3 (± 1.5)	3.2 (± 1.0)

### Dissolved nutrients in RAS and hydroponics

Dissolved nutrients in RAS and hydroponics were determined weekly and are presented in Table 4. In all three RAS, a constant accumulation of nitrate was observed over the 154 d experimental period, increasing from 15.7–19.8 mg L^-1^ during the first sampling interval up to 65.9–100.8 mg L^-1^ at the end of the experimental period. In Hydro D, nitrate concentration increased from 98.8 mg L^-1^ NO_3_^-^-N to more than 170 mg L^-1^ from the third month on. During the entire experimental period, nitrite in all fish and hydroponic units was very low (≤ 0.1 mgL^-1^ NO_2_^-^-N). Ammonium revealed concentrations ≤ 0.4 mgL^-1^ NH_4_^+^-N in the RAS units and Hydro C. Only in Hydro D a maximum of 6.4 mg L^-1^ NH_4_^+^-N was observed at the beginning of the experimental period, which constantly decreased to low levels comparable the other systems. In all fish and hydroponic units, the phosphate concentration decreased to 5.6–9.6 mg L^-1^ towards the end of the experimental period. Still, during the first two months, phosphate concentrations were more than 2-fold higher in Hydro D than in Hydro C. Potassium concentrations in both aquaponic systems were generally higher than in the RAS A, but levels in all units ranged between 17 and 50 mg L^-1^. Exceptionally low potassium concentrations < 5 mg L^-1^ were only observed during the last month in Hydro D. Also, no substantial differences were observed with respect to the chloride concentrations in the fish units and Hydro C, ranging between 29–46.5 mg L^-1^ Cl^-^. Only in Hydro D an accumulation of chloride from 46 mg L^-1^ to 89.7 mg L^-1^ Cl^-^ was observed. Sulfate ranged between 157.5 and 195 mg L^-1^, only in Hydro D substantially elevated concentrations (295–660 mg L^-1^) were observed. Similarly, calcium was 3-fold increased in Hydro D (362.8–558.5 mg L^-1^) compared to Hydro C (119.9–148.5 mg L^-1^). Iron and magnesium were within the same range between all RAS and Hydro C; only Hydro D revealed higher concentrations.

**Table 4:** Dissolved nutrients in the fish (RAS A, C, D) and hydroponic units (Hydro C, Hydro D) of a conventional aquaculture reference (A), a coupled (C) and a decoupled (D) aquaponic system, assessed over the experimental period of 154 days (07.04–07.09.2015). Nutrients in RAS C correspond to the nutrients in Hydro C, since both are arranged as coupled aquaponic system.

RAS / Hydro	sampling intervals	NO_3_^-^-N[mgL^-1^]	NO_2_^-^-N[mgL^-1^]	NH_4_^+^-N[mgL^-1^]	PO_4_^3-^[mgL^-1^]	K^+^[mgL^-1^]	Ca^2+^[mgL^-1^]	Mg^2+^[mgL^-1^]	SO_4_^2-^[mgL^-1^]	Cl^-^ [mgL^-1^]	Fe^2+^[mgL^-1^]
**A**	07.04.-06.05.15	15.7 ± (4.7)	0.09 ± (0.08)	0.12 ± (0.06)	14.8 ± (0.9)	22.0 ± (1.4)	123.4 ± (0.8)	14.1 ± (0.0)	165 ± (35.4)	36.5 ± (7.1)	0.01 ± (0.00)
	07.05.-08.06.15	27.6 ± (3.6)	0.07 ± (0.03)	0.09 ± (0.04)	10.4 ± (2.2)	21.6 ± (1.8)	130.6 ± (6.0)	21 ± (7.7)	178.8 ± (2.5)	30.3 ± (5.9)	0.01 ± (0.01)
	09.06.-09.07.15	30.4 ± (10.9)	0.06 ± (0.02)	0.16 ± (0.16)	6.7 ± (1.1)	19.3 ± (4.8)	134.1 ± (5.8)	17.7 ± (3.0)	161.3 ± (6.3)	29.0 ± (2.3)	0.01 ± (0.01)
	10.07.-06.08.15	52.3 ± (4.8)	0.04 ± (0.01)	0.08 ± (0.01)	6.4 ± (0.9)	18.7 ± (2.1)	136.8 ± (5.4)	16.3 ± (0.9)	168.8 ± (2.5)	30.6 ± (1.8)	0.01 ± (0.01)
	07.08.-07.09.15	65.9 ± (6.1)	0.04 ± (0.01)	0.07 ± (0.04)	5.6 ± (0.8)	17.0 ± (1.4)	141.1 ± (10.8)	16.2 ± (1.2)	160 ± (5.0)	38.2 ± (5.8)	0.01 ± (0.01)
**C Hydro C**	07.04.-06.05.15	19.8 ± (6.2)	0.05 ± (0.00)	0.06 ± (0.01)	17.1 ± (0.4)	27.8 ± (3.9)	119.8 ± (0.8)	13.8 ± (0.7)	175 ± (35.4)	39.8 ± (7.4)	0.01 ± (0.01)
	07.05.-08.06.15	36.2 ± (14.9)	0.08 ± (0.03)	0.04 ± (0.01)	12.8 ± (2.0)	28.0 ± (2.6)	138.5 ± (11.6)	21.9 ± (7.6)	197.5 ± (2.9)	31.0 ± (6.1)	0.01 ± (0.01)
	09.06.-09.07.15	59.2 ± (14.0)	0.07 ± (0.02)	0.15 ± (0.12)	9.8 ± (0.7)	40.8 ± (10.6)	148.5 ± (5.3)	19.4 ± (1.8)	191.3 ± (8.5)	34.6 ± (3.6)	0.01 ± (0.01)
	10.07.-06.08.15	65.3 ± (11.5)	0.05 ± (0.01)	0.06 ± (0.01)	8.3 ± (1.6)	38.7 ± (19.4)	144.8 ± (7.3)	19.3 ± (1.8)	195 ± (7.1)	39.1 ± (2.3)	0.02 ± (0.01)
	07.08.-07.09.15	72.8 ± (19.9)	0.05 ± (0.02)	0.06 ± (0.02)	6.3 ± (0.9)	27.3 ± (4.0)	149.2 ± (2.8)	20.4 ± (1.3)	190 ± (18.0)	46.5 ± (7.5)	0.02 ± (0.02)
**D**	07.04.-06.05.15	17.5 ± (7.4)	0.02 ± (0.01)	0.03 ± (0.01)	16.9 ± (3.8)	22.0 ± (3.5)	125.2 ± (1.1)	14.9 ± (0.5)	157.5 ± (10.6)	38 ± (10.6)	0.01 ± (0.00)
	07.05.-08.06.15	27.1 ± (7.0)	0.06 ± (0.01)	0.05 ± (0.02)	14.2 ± (0.9)	25.4 ± (2.9)	140.2 ± (8.6)	22.9 ± (8.2)	173.8 ± (6.3)	29.7 ± (6.5)	0.01 ± (0.00)
	09.06.-09.07.15	50.4 ± (9.6)	0.06 ± (0.02)	0.12 ± (0.19)	13.4 ± (0.9)	40.0 ± (10.1)	152.0 ± (4.4)	19.7 ± (1.2)	186.3 ± (9.5)	33.8 ± (3.0)	0.01 ± (0.01)
	10.07.-06.08.15	77.6 ± (6.5)	0.05 ± (0.02)	0.06 ± (0.01)	11.9 ± (1.1)	41.7 ± (20.8)	149.0 ± (2.5)	23.4 ± (7.3)	193.8 ± (8.5)	38.5 ± (1.3)	0.01 ± (0.01)
	07.08.-07.09.15	100.8 ± (10.8)	0.05 ± (0.02)	0.05 ± (0.02)	9.6 ± (1.2)	29.8 ± (2.9)	149.3 ± (6.3)	19.7 ± (0.9)	183.3 ± (10.4)	33.7 ± (8.9)	0.01 ± (0.01)
Hydro D	07.04.-06.05.15	98.8 ± (23.7)	0.07 ± (0.08)	3.60 ± (0.28)	29.1 ± (6.8)	207.5 ± (3.5)	556.0 ± (90.5)	49.5 ± (10.6)	295 ± (49.5)	46.0 ± (9.9)	0.01 ± (0.00)
	07.05.-08.06.15	136.9 ± (58.4)	0.02 ± (0.02)	2.25 ± (3.03)	26.1 ± (8.5)	41.8 ± (30.5)	362.8 ± (61.9)	36.4 ± (9.9)	515 ± (256.8)	36.5 ± (9.3)	0.11 ± (0.14)
	09.06.-09.07.15	175.0 ± (38.7)	0.01 ± (0.01)	0.64 ± (0.67)	12.9 ± (1.9)	50.0 ± (41.4)	558.5 ± (137.4)	57.3 ± (27.2)	660 ± (468.5)	76.6 ± (43.3)	0.05 ± (0.03)
	10.07.-06.08.15	207.5 ± (70.1)	0.01 ± (0.00)	0.08 ± (0.05)	7.2 ± (2.9)	24.0 ± (25.6)	442.8 ± (43.4)	56.3 ± (21.9)	470 ± (194.9)	69.1 ± (15.1)	0.12 ± (0.08)
	07.08.-07.09.15	174.5 ± (38.5)	0.00 ± (0.02)	0.02 ± (0.01)	6.7 ± (1.7)	4.2 ± (4.4)	482.0 ± (147.8)	50.1 ± (16.9)	373.3 ± (50.3)	89.7 ± (23.8)	0.10 ± (0.04)

**Fig 2:**
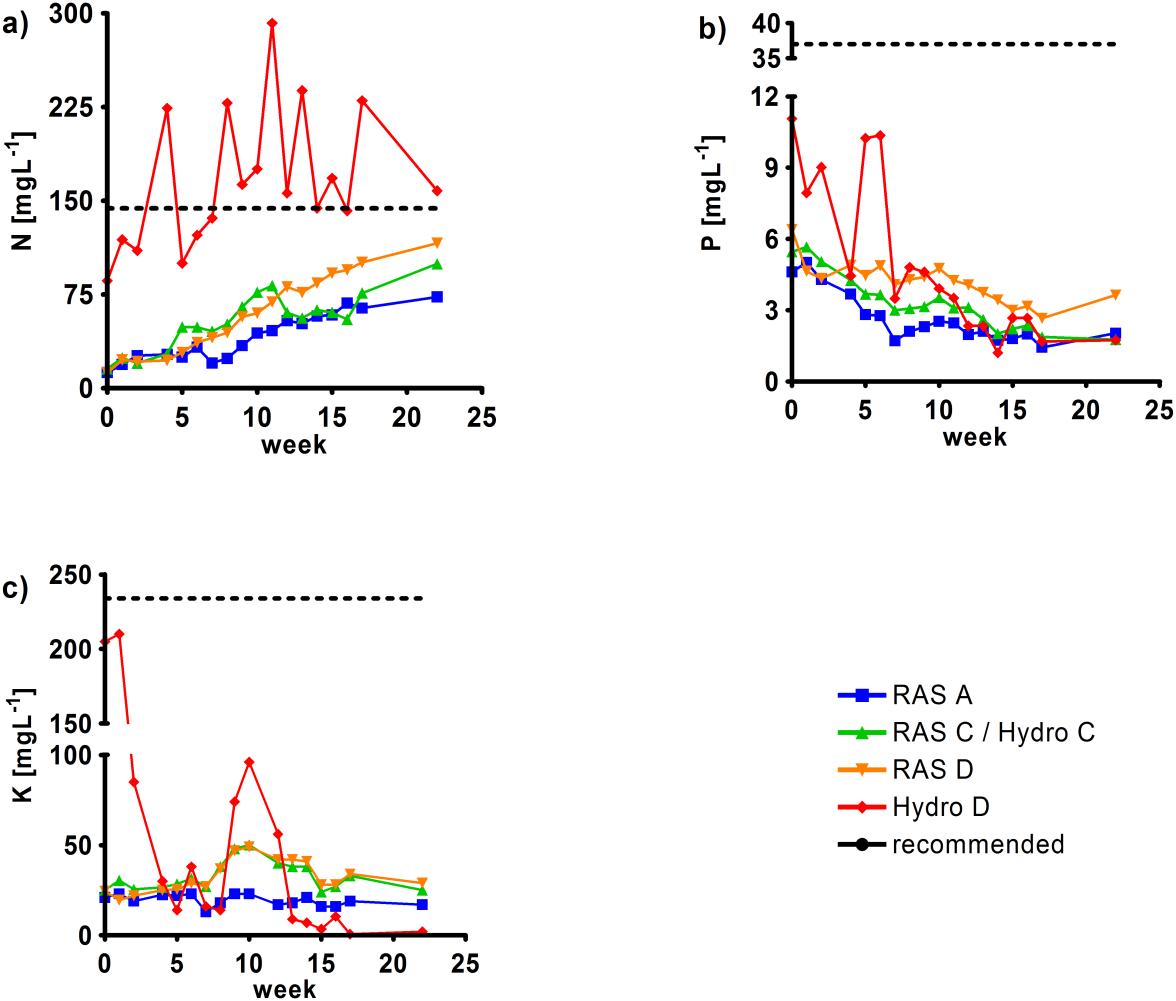
Development of the key nutrients (N, P, K) for plant production in the fish (RAS) and hydroponic (Hydro) units of the coupled (RAS C/Hydro C) and decoupled (RAS D, Hydro D) aquaponic system compared to the control (RAS A) over 22 weeks. Nutrients in RAS C correspond to the nutrients in Hydro C since both are arranged as coupled aquaponic system. Recommended nutrient requirements for tomato production are indicated (dashed line).

In Fig 2 the development of key nutrients (N, P, K) is presented over the experimental period with respect to recommended concentrations for tomato production. In all RAS systems there was a general accumulation of N without reaching the recommended threshold (dashed line). A constant decrease of P and a more or less stable concentration of K with a peak in the middle of the experimental period was observed. Again recommended concentrations were not reached and in the case of K stayed far beyond the recommended threshold. In all cases RAS A showed the lowest concentrations of key nutrients and highest observed concentrations occurred in Hydro D. Here, recommended levels of N were often reached or even exceeded. The K concentration was just close to optimum conditions towards the start of experiments but lowered considerably towards the end of the experimental period. Also, during the first third of the experimental period, the P concentration was frequently higher than in all other systems but showed the same decreasing trend towards the end.

### Elemental composition of plants, fish and sludge

In general, composition of plant leaves and tomatoes revealed no major differences of the respective plant parts between Hydro C and Hydro D, neither in ICP-OES analysis nor C/N ratio (Table 5). Only the phosphate contents of tomatoes and leaves were lower in Hydro C compared to Hydro D. In addition, sodium concentrations in the fruit were slightly higher in Hydro D compared to Hydro C. Mean elemental composition of fish and sludge were also determined and are provided to complete the picture of the overall aquaponic system.

**Table 5:** Elemental analysis (ICP-OES and C/N) of plant leaves and tomatoes harvested from the hydroponic unit of the coupled (Hydro C) and the decoupled (Hydro D) aquaponic system after 30 d, 63 d, 94 d, 122 d and 154 d. Additionally data for fish and sludge are presented.

system	Experimentalperiod / date	sample	Ca[g kg^-1^]	K[g kg^-1^]	Mg[g kg^-1^]	Na[g kg^-1^]	P[g kg^-1^]	N[%]	C[%]	C/N
**Hydro C**	07.05.-08.06.15	leaf	30.4 ± (1.9)	45.4 ± (1.3)	4.4 ± (0.2)	0.3 ± (0.0)	5.1 ± (0.2)	3.4 (± 0.1)	36.6 (± 0.1)	10.9 (± 0.5)
	09.06.-09.07.15	leaf	32.4 ± (3.0)	40.3 ± (7.3)	4.8 ± (0.5)	0.3 ± (0.0)	4.4 ± (0.3)	3.0 (± 0.2)	37.5 (± 0.4)	12.3 (± 0.8)
	10.07.-06.08.15	leaf	26.0 ± (2.3)	35.3 ± (2.2)	3.9 ± (0.2)	0.3 ± (0.0)	4.7 ± (0.3)	3.2 (± 0.2)	38.1 (± 0.3)	11.9 (± 0.8)
	07.08.-07.09.15	leaf	34.0 ± (3.6)	33.2 ± (3.2)	3.8 ± (0.4)	0.4 ± (0.0)	4.3 ± (0.5)	2.6 (± 0.3)	37.1 (± 0.3)	14.2 (± 1.3)
	07.05.-08.06.15	tomato	2.2 ± (1.0)	47.5 ± (0.2)	1.3 ± (0.1)	0.3 ± (0.0)	4.6 ± (0.2)	2.0 (± 0.1)	38.8 (± 0.4)	19.1 (± 1.3)
	09.06.-09.07.15	tomato	2.1 ± (0.3)	41.6 ± (2.5)	1.4 ± (0.1)	0.2 ± (0.0)	4.3 ± (0.2)	1.7 (± 0.2)	39.9 (± 0.2)	24.3 (± 3.2)
	10.07.-06.08.15	tomato	1.3 ± (0.3)	41.0 ± (1.6)	1.5 ± (0.0)	0.3 ± (0.0)	4.0 ± (0.5)	2.0 (± 0.2)	39.3 (± 0.3)	19.8 (± 1.5)
	07.08.-07.09.15	tomato	1.1 ± (0.1)	42.0 ± (4.4)	1.5 ± (0.3)	0.3 ± (0.2)	4.4 ± (0.3)	2.0 (± 0.5)	39.7 (± 0.7)	20.3 (± 4.5)
**Hydro D**	07.05.-08.06.15	leaf	26.7 ± (4.3)	39.9 ± (2.4)	3.9 ± (0.2)	1.1 ± (0.1)	2.7 ± (0.1)	3.9 (± 0.1)	38.7 (± 0.7)	10.1 (± 0.4)
	09.06.-09.07.15	leaf	23.1 ± (3.3)	46.0 ± (0.9)	3.2 ± (0.3)	1.3 ± (0.1)	2.6 ± (0.4)	3.2 (± 0.1)	39.1 (± 0.5)	12.3 (± 0.4)
	10.07.-06.08.15	leaf	25.5 ± (2.8)	36.0 ± (1.6)	4.0 ± (0.2)	0.9 ± (0.1)	2.9 ± (0.2)	3.8 (± 0.1)	39.1 (± 0.6)	10.4 (± 0.2)
	07.08.-07.09.15	leaf	26.7 ± (11.1)	32.8 ± (7.5)	3.2 ± (0.9)	0.7 ± (0.2)	2.6 ± (0.5)	3.2 (± 0.4)	38.9 (± 1.0)	12.2 (± 2.1)
	07.05.-08.06.15	tomato	1.7 ± (0.2)	45.6 ± (5.2)	1.2 ± (0.2)	0.4 ± (0.0)	3.7 ± (0.6)	2.1 (± 0.4)	39.5 (± 0.4)	19.6 (± 4.3)
	09.06.-09.07.15	tomato	1.3 ± (0.1)	36.1 ± (3.9)	1.3 ± (0.1)	0.5 ± (0.0)	3.1 ± (0.6)	2.0 (± 0.2)	39.4 (± 0.4)	20.1 (± 1.9)
	10.07.-06.08.15	tomato	1.1 ± (0.4)	40.5 ± (2.9)	1.3 ± (0.1)	0.4 ± (0.1)	3.0 ± (0.8)	2.0 (± 0.3)	39.6 (± 0.1)	20.2 (± 2.9)
	07.08.-07.09.15	tomato	1.2 ± (0.5)	41.5 ± (2.8)	1.4 ± (0.1)	0.4 ± (0.2)	3.4 ± (0.6)	2.1 (± 0.4)	39.3 (± 0.1)	19.0 (± 3.3)
**RAS A-C-D**	09.09.2015	fish	31.7 (± 1.0)	1.5 (± 0.1)	2.1 (± 0.1)	0.7 (± 0.0)	17.7 (± 0.5)	7.4 (± 0.2)	56.5 (± 3.3)	7.6 (± 0.5)
**RAS C-D**	09.09.2015	sludge	11.9 (± 5.8)	8.3 (± 0.1)	0.6 (± 0.1)	3.5 (± 0.1)	8.9 (± 2.8)	4.1 (± 0.2)	36.6 (± 1.0)	9.0 (± 0.6)

### TSS and loss of solids in RAS

**Fig 3:**
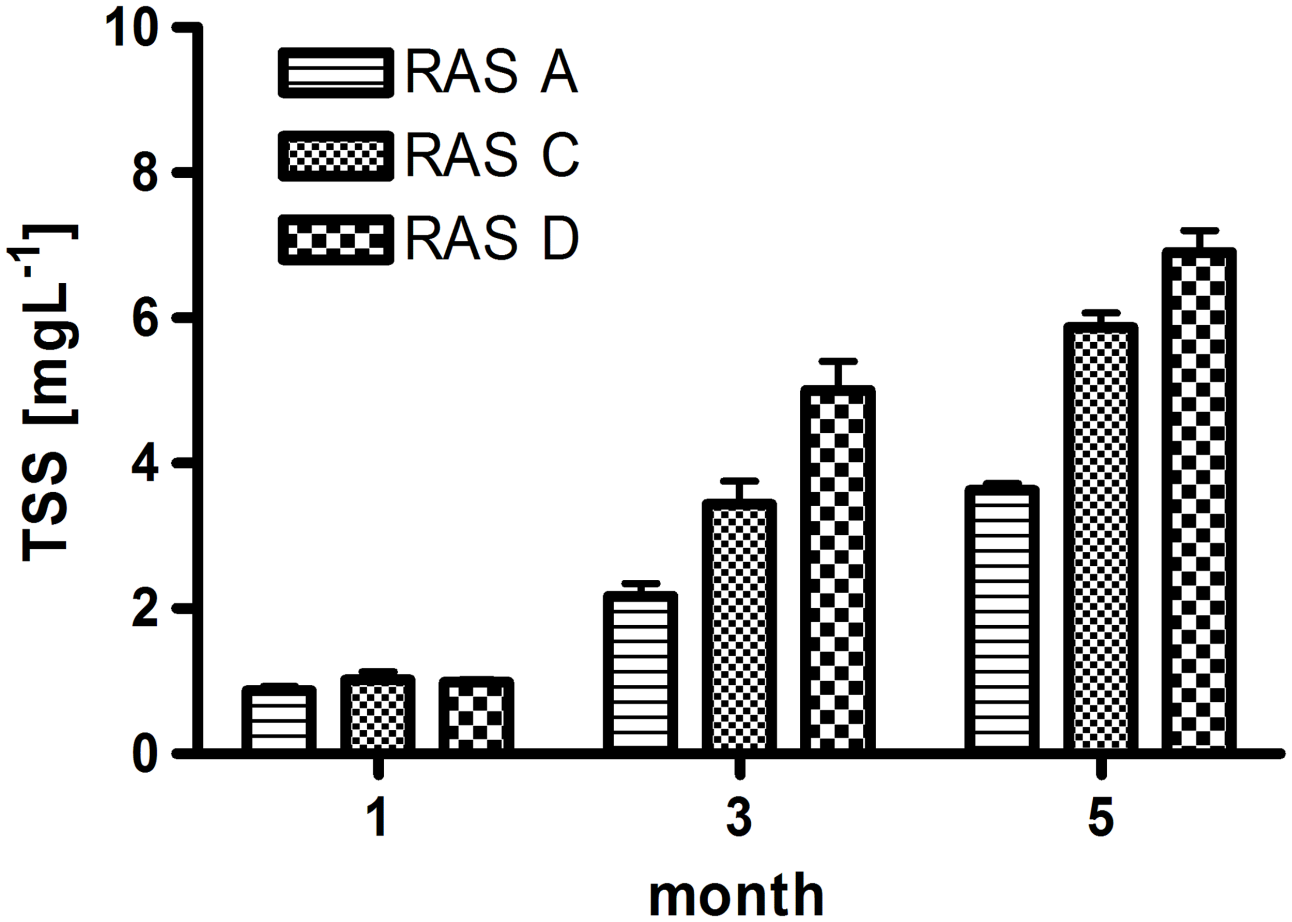
Total suspended solids (TSS, g dry weight/L rearing water) in the fish unit of the aquaculture reference (RAS A), the coupled (RAS C) and decoupled (RAS D) aquaponic system after 30 d (1 month), 94 d (3 month) and 154 d (5 month). Presented are the means (± SD, n = 3).

TSS was determined three times in triplicate (n = 3) over the experimental period for each RAS (Fig 3). During the first sampling interval, all three RAS had a comparable low TSS of about 0.75–1.15 mg L^-1^. Thereafter, a constant increase of TSS was observed in all RAS over the experimental period, revealing highest removal in the RAS A equipped with a drum filter. Towards the last month of the experimental period TSS was highest in RAS D (6.9 (± 0.5)) and lowest in RAS A (3.6 (± 0.2)). TSS in the RAS arranged as coupled system (RAS C) was slightly lower compared to the decoupled aquaponic system (RAS D).

The removal of solids in the clarifier (Table 6) due to the weekly cleaning was within the same range between the two fish units RAS C and RAS D and ranged around 1.8–2.0 g dry weight * L^-1^. For the clarifiers used (V = 1.5 m^3^) a weekly loss of 2.7–3 kg of organic matter (dry weight) was thus calculated here.

**Table 6:** Solid removal (g dry weight * L^-1^) in the fish unit of the coupled (RAS C) and the decoupled (RAS D) aquaponic system due to weekly cleaning of the clarifier (V = 1.5 m^3^) over three consecutive weeks. Presented are the means (± SD, n = 5).

sampling [week]	RAS C[g L^-1^]	RAS D [g L^-1^]
1	1.9 (± 0.18)	2.0 (± 0.17)
2	1.8 (± 0.11)	2.0 (± 0.04)
3	1.8 (± 0.06)	1.9 (± 0.04)
mean	1.8 (± 0.07)	2.0 (± 0.09)

### Estimated fate of nitrogen in RAS and aquaponics

**Fig 4:**
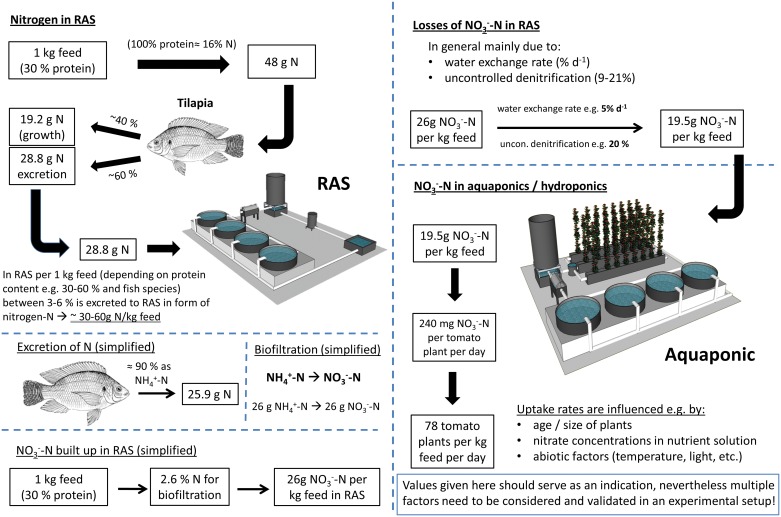
Estimated fate of nitrogen in RAS and potential nitrate supply to the crop plants (tomatoes) in aquaponics.

For a better estimation of nitrate accumulation in RAS and potential nitrate supply of crop plants (e.g. tomatoes) in aquaponics per kg feed fed to the fish, a simplified schematic illustration of the fate of nitrogen (mainly nitrate) was developed here (Fig 4).

## Discussion

Here, a new approach for aquaponics is presented, comparing an innovative decoupled (2-loop system) and a coupled (1-loop system) medium scale aquaponic system experimentally in a pilot study. There are some obvious reasons why a decoupling of RAS and hydroponics in a commercial aquaponic facility is favorable compared to a classical coupled approach. The most important ones should be discussed in the following section based on the results of this pilot study and supplemented by some theoretical considerations.

In our pilot study fish were stocked at around 40 kg/m^3^ providing the nutrients for plant growth in the hydroponics according to Table 4. The amount of fertilizer was continuously reduced with increasing biomass in the systems. Thereby, tomato harvest in the aquaponic systems differed substantially (Table 1). In the decoupled system 123.5 kg of tomatoes were obtained compared to 90.9 kg in the coupled system, corresponding to 36% higher tomato yield in the decoupled system. Equal amounts of fertilizers (Table 2) were added in both systems, allowing a substantially improved nutrient supply in the decoupled but not in the coupled system due to the increased water volume of the coupled system. Thereby, more leave, root and stem biomass was produced in the coupled system. This has been reported before and is often related to suboptimal nutrient supply [28]. Here, the increase of root surface and the subsequent change of shoot to root ratio boost the nutrient uptake and have been frequently observed [28, 29]. Suboptimal plant growth in RAS C had probably two main reasons. In a coupled aquaponic system the pH is generally not optimal for plant growth [9] and thus not all nutrients are equally available. At the same time, fertilizers added are diluted within coupled systems due to the higher water volume encompassing the fish rearing unit, compared to decoupled systems, which allows exclusive supplementation in the hydroponic unit. Of course fertilizer applications could be increased in the coupled system, but this is neither economical nor a good solution in the context of animal welfare. Supplementation of substantial amounts of nutrients to the fish culture bares the risks of acute or chronic toxicity [30, 31]. Further, intentionally reducing water quality for the fish irrespective the degree of adverse effects is hardly acceptable with regard to the code of best practice and will also threaten the acceptance of the public as well as the envisioned potential for a sustainability label. Nevertheless, tilapia has been shown to be relatively robust in terms of nitrate and no adverse effects below 500 mg L^-1^ NO_3_^-^-N have been observed [32]. For other ions this is mostly unclear and, if at all recommendable, optimal fertilizer formulations have to be evaluated for each fish species cultured.

In a previous study, [5] tested for the first time a prototype decoupled aquaponic systems reporting a yield of 8.89 kg plant^-1^ within 9 month. In the present study we observed a comparable tomato production of 8.2 kg plant^-1^ (Hydro D) compared to 6.1 kg plant^-1^ (Hydro C) in the coupled system within only 6 month. High greenhouse temperatures > 35°C in June and July probably contributed to a reduced development of flowers and thus fruits in that period (Table 1). The relationship of high temperatures and decreased flower development was already reported by [33, 34]. Here, no cooling was applied, but obviously, decoupling allows such a better temperature control, which could compromise the growth of tilapia in coupled systems. A lack of pollination could be another reason for reduced flowering [35], but this was done manually at least twice a week.

In addition to harvest yield and fruit composition, composition of the leaves was determined on a regular basis (Table 5) to monitor the nutrient status as suggested for fertilizer programs [18]. Results revealed that the N, P and K content of all leaves were within the normal range (N: 2–5% of dry weight, P: 0.25–0.6%, K: 2.8–4%). Also, concentrations of Ca (1–5%) and Mg (0.2–0.8%) indicated no obvious deficiencies.

In contrast to the tomato production, harvest of fish revealed no differences in growth performance and feed conversion, neither in coupled (RAS C), decoupled (RAS D) nor classical aquaculture (RAS A) (Table 2). Here, the average FCR ranged between 1.2–1.3 and is representative for commercial aquaculture [36–38]. The SGR was moderate with an average of 1.0 and lowered with increasing fish size as described elsewhere [37, 39, 40].

A higher water consumption of 5–6% per day was reported in the state-of-the-art aquaculture system (RAS A) compared to the aquaponic systems ranging between 2–3.6%. This is mainly a consequence of the backwash in the automatic drum filter compared to the clarifiers in the aquaponic units. Nevertheless, an average water consumption of 5.3% of RAS volume per day for RAS A (Table 2) is within the range for conventional RAS as reported elsewhere [41, 42]. Also, water quality was similar between the three RAS units and within the optimal range for tilapia. Here, both ammonia (≤ 0.15 mg L^-1^ TAN) as well as nitrite (≤ 0.1 mgL^-1^ NO_2_^-^-N) were far below levels generally considered critical in fish.

In RAS, nitrification is one of the key processes, converting ammonia and providing nitrate for the plants (Fig 4). For optimal conversion, pH should be kept around 7 or higher [16]. The control of pH in RAS is mainly achieved by the addition of limestone to compensate drops in pH as a consequence of nitrification itself and CO_2_ accumulation from respiration. In contrast, a pH of 7 is not optimal for nutrient supply of plants since availability of most nutrients is best at pH 5.5–6.5 [18]. Vice versa, pH < 6.5 in the RAS affects nitrification efficiency with subsequently accumulation of ammonium and nitrite. At pH ≤ 6 nitrification finally ceases [17] and ammonium would accumulate in the process water of RAS. High ammonium concentrations in RAS bare the risk of ammonia toxicity for fish [43] even though this is mainly problematic when the pH is high (> 8) [44]. But the processes within a classical aquaponic system are interconnected and more complex than in a single RAS. Ammonium toxicity for plants can occur already at concentrations as low as 1.8–9 mg L^-1^ NH_4_^+^ and tomatoes are among the more sensitive plants [45]. Additionally at high ammonia concentrations, ammonia uptake by the plants may further decrease the pH (<5), especially in summer, due to the excretion of protons by the roots [46].

The main advantage of decoupled systems is, that no compromises have to be made in terms of optimal production parameters for both, fish and plants. Only here, nutrient solution (e.g. addition of fertilizer in hydroponics, pH regulation, temperature adjustment) as well as temperature can be adjusted for each production unit. As discussed above, addition of fertilizers challenges animal welfare concerns. Also, economic feasibility may require discontinuous production, particularly with regard to the plant crop. As a consequence, nutrient requirements for plants can vary and nutrient supply by the fish needs to be adapted dynamically. In the coupled system, at fish densities between 39 (start) and 65 kg/m^3^ (end) nitrate peaked at 99.5 mg L^-1^ NO_3_^-^-N and was thus below the recommended nutrient requirements of tomato plants of >140 mg L^-1^ NO_3_^-^-N [13]. Similarly, P and K did not meet minimal requirements, illustrating the need for nutrient supplementation or alternatively, compromising on the production. Still, better nutrient supply can be achieved at higher densities and tilapia can be grown up to 120 kg/m^3^, if oxygenation is applied [36].

As illustrated in Figs 2 and 4, nitrogen, mainly in the form of nitrate, is the predominant macronutrient recycled from the fish unit in aquaponics. P and K are often scarce in RAS water and need to be supplemented to support the plant crop [9]. This was also observed in the present study and, again, decoupling allowed for specific supplementation using commercial fertilizers. Nevertheless, P and K can be recycled from the fish sludge, increasing the overall sustainability of the system [30]. Here, aerobic mineralization processes may be regarded superior since significant N losses have been reported for anaerobic reactors due to denitrification.

Further, irrespective the system used, pathogen treatment or health concerns may require immediate decoupling. So far, disease transmission between fish and plant units has not been evaluated sufficiently, but needs to be addressed in the near future. Decoupling allows more managerial flexibility, including UV or ultrasound disinfection [47] and disease therapy or specific countermeasures for fish [48] or plant treatment [49].

Overall both, decoupled and classical aquaponics, have their pro and cons. For small scale production or the production of plants with low nutrient requirements like lettuce or herbs, classical systems are probably easier to handle involving fewer factors to be monitored. For large scale professional production (as well as complex, high nutrient requirements) a decoupled system is recommended, but the complexity of the system in terms of management (e.g. automation) and labor needs to be considered.

## Conclusions

In this pilot study, comparing the performance of decoupled aquaponic systems and coupled aquaponics, considerably higher plant production was observed in the decoupled approach, whereas fish production in all systems (including a state-of-the-art aquaculture unit) revealed comparable growth performance and feed conversion. The main reasons for better performance of decoupled systems were attributed to the independent regulation of the pH and dynamic adaptation of nutrient concentrations. At moderate densities assessed here (40–65 kg/m^3^) optimal nutrient supply most probably requires supplementation and thus advocates decoupling. In terms of professionalization and improvement of production performance decoupled systems are more likely to meet the demand of producers, since optimal conditions can be controlled for both, fish and plants, separately and imbalances can be managed adequately. Based on the results a decoupling of RAS and hydroponics for an optimized production is recommended, safeguarding in particular the animal welfare in the fish unit.

## Supporting information

S1 Table Rearing conditions (dissolved oxygen (O_2_), pH, temperature and conductivity) in the fish (RAS) and hydroponic (Hydro) units of a conventional aquaculture reference (A), a coupled (C) and a decoupled (D) aquaponic system, assessed over the experimental period of 154 days (07.04–07.09.2015). (DOCX)

S2 Table Dissolved nutrients in the fish (RAS A, C, D) and hydroponic units (Hydro C, Hydro D) of a conventional aquaculture reference (A), a coupled (C) and a decoupled (D) aquaponic system, assessed over the experimental period of 154 days (07.04–07.09.2015). Nutrients in RAS C correspond to the nutrients in Hydro C, since both are arranged as coupled aquaponic system.
(DOCX)

S3 Table Elemental analysis (ICP-OES) of plant leaves and tomatoes harvested from the hydroponic unit of the coupled (Hydro C) and the decoupled (Hydro D) aquaponic system after 30 d, 63 d, 94 d, 122 d and 154 d. Additionally data for fish and sludge are presented.
(DOCX)

S4 Table Elemental analysis (C/N) of plant leaves and tomatoes harvested from the hydroponic unit of the coupled (Hydro C) and the decoupled (Hydro D) aquaponic system after 30 d, 63 d, 94 d, 122 d and 154 d. Additionally, data for fish and sludge are presented.
(DOCX)

S5 Table Solid removal (g dry weight * L^-1^) in the fish unit of the coupled (RAS C) and the decoupled (RAS D) aquaponic system due to weekly cleaning of the clarifier (V = 1.5 m^3^) over three consecutive weeks. (DOCX)

S6 Table Total suspended solids (TSS, g dry weight/L rearing water) in the fish unit of the aquaculture reference (RAS A), the coupled (RAS C) and decoupled (RAS D) aquaponic system after 30 d (1 month), 94 d (3 month) and 154 d (5 month). SD–standard deviation.
(DOC)
