# Supplementary material for: Decoupled systems on trial: Eliminating bottlenecks to improve aquaponic processes
Source: PLoS One. 2017 Sep 28;12(9):e0183056. doi: 10.1371/journal.pone.0183056 (PMC5619720; doi:10.1371/journal.pone.0183056)
Supplement: S1 Table — (DOCX) [file pone.0183056.s001.docx]

S1 Table: Rearing conditions (dissolved oxygen (O_2_), pH, temperature and conductivity) in the fish (RAS) and hydroponic (Hydro) units of a conventional aquaculture reference (A), a coupled (C) and a decoupled (D) aquaponic system, assessed over the experimental period of 154 days (07.04 - 07.09.2015).

| **Date** | **RAS / Hydro** | **system** | **O_2_ [mgL^-1^]** | **pH** | **temperature [°C]** | **conductivity [mScm^-1^]** |
| --- | --- | --- | --- | --- | --- | --- |
|  |  |  |  |  |  |  |
| 07.04.2015 | Hydro | D | 9.1 | 6.91 | 20.2 | 3.9 |
| 07.04.2015 | RAS | A | 8.1 | 6.54 | 26.1 | 1 |
| 07.04.2015 | RAS | A | 8 | 6.54 | 26.1 | 1 |
| 07.04.2015 | RAS | A | 9.2 | 6.54 | 26.1 | 1 |
| 07.04.2015 | RAS | A | 8.3 | 6.54 | 26.1 | 1 |
| 07.04.2015 | RAS | C | 9 | 6.91 | 26.2 | 1.1 |
| 07.04.2015 | RAS | C | 8.7 | 6.91 | 26.2 | 1.1 |
| 07.04.2015 | RAS | C | 8.2 | 6.91 | 26.2 | 1.1 |
| 07.04.2015 | RAS | C |  | 6.91 | 26.2 | 1.1 |
| 07.04.2015 | RAS | D | 8.9 | 7.37 | 26.4 | 1.1 |
| 07.04.2015 | RAS | D | 9 | 7.37 | 26.4 | 1.1 |
| 07.04.2015 | RAS | D | 7.1 | 7.37 | 26.4 | 1.1 |
| 07.04.2015 | RAS | D | 7.7 | 7.37 | 26.4 | 1.1 |
| 08.04.2015 | Hydro | D | 8.78 | 7.21 | 22.1 | 3.14 |
| 08.04.2015 | RAS | A | 6.2 | 7.29 | 25.8 | 1 |
| 08.04.2015 | RAS | A | 6.2 | 7.29 | 25.8 | 1 |
| 08.04.2015 | RAS | A | 7.2 | 7.29 | 25.8 | 1 |
| 08.04.2015 | RAS | A | 5.9 | 7.29 | 25.8 | 1 |
| 08.04.2015 | RAS | C | 6.5 | 7.3 | 26.4 | 1.1 |
| 08.04.2015 | RAS | C | 6.7 | 7.3 | 26.4 | 1.1 |
| 08.04.2015 | RAS | C | 7.6 | 7.3 | 26.4 | 1.1 |
| 08.04.2015 | RAS | C | 7 | 7.3 | 26.4 | 1.1 |
| 08.04.2015 | RAS | D | 7.7 | 7.49 | 26.5 | 1.1 |
| 08.04.2015 | RAS | D | 6.5 | 7.49 | 26.5 | 1.1 |
| 08.04.2015 | RAS | D | 5.8 | 7.49 | 26.5 | 1.1 |
| 08.04.2015 | RAS | D | 7.4 | 7.49 | 26.5 | 1.1 |
| 09.04.2015 | Hydro | D | 8.91 | 7.32 | 22.3 | 3.21 |
| 09.04.2015 | RAS | A | 5.5 | 7.13 | 25.8 | 1 |
| 09.04.2015 | RAS | A | 5.7 | 7.13 | 25.8 | 1 |
| 09.04.2015 | RAS | A | 7.1 | 7.13 | 25.8 | 1 |
| 09.04.2015 | RAS | A | 4.9 | 7.13 | 25.8 | 1 |
| 09.04.2015 | RAS | C | 5.5 | 7.02 | 26.7 | 1.1 |
| 09.04.2015 | RAS | C | 5.7 | 7.02 | 26.7 | 1.1 |
| 09.04.2015 | RAS | C | 7.4 | 7.02 | 26.7 | 1.1 |
| 09.04.2015 | RAS | C | 4.9 | 7.02 | 26.7 | 1.1 |
| 09.04.2015 | RAS | D | 6.3 | 7.32 | 26.4 | 1.1 |
| 09.04.2015 | RAS | D | 4.3 | 7.32 | 26.4 | 1.1 |
| 09.04.2015 | RAS | D | 4.4 | 7.32 | 26.4 | 1.1 |
| 09.04.2015 | RAS | D | 7.4 | 7.32 | 26.4 | 1.1 |
| 10.04.2015 | Hydro | D | 8.53 | 7.39 | 22.8 | 3.6 |
| 10.04.2015 | RAS | A | 6.2 | 7.2 | 26.2 | 1.1 |
| 10.04.2015 | RAS | A | 3.9 | 7.2 | 26.2 | 1.1 |
| 10.04.2015 | RAS | A | 7.5 | 7.2 | 26.2 | 1.1 |
| 10.04.2015 | RAS | A | 5.1 | 7.2 | 26.2 | 1.1 |
| 10.04.2015 | RAS | C | 7.6 | 6.89 | 26.5 | 1.1 |
| 10.04.2015 | RAS | C | 6.5 | 6.89 | 26.5 | 1.1 |
| 10.04.2015 | RAS | C | 7.8 | 6.89 | 26.5 | 1.1 |
| 10.04.2015 | RAS | C | 4.5 | 6.89 | 26.5 | 1.1 |
| 10.04.2015 | RAS | D | 4.4 | 7.41 | 26.5 | 1.1 |
| 10.04.2015 | RAS | D |  | 7.41 | 26.5 | 1.1 |
| 10.04.2015 | RAS | D | 4.8 | 7.41 | 26.5 | 1.1 |
| 10.04.2015 | RAS | D | 7.7 | 7.41 | 26.5 | 1.1 |
| 11.04.2015 | Hydro | D | 8.01 | 7.07 | 22.5 |  |
| 11.04.2015 | RAS | A | 5.4 | 6.99 | 26.9 |  |
| 11.04.2015 | RAS | A |  | 6.99 | 26.9 |  |
| 11.04.2015 | RAS | A | 7.2 | 6.99 | 26.9 |  |
| 11.04.2015 | RAS | A | 4.06 | 6.99 | 26.9 |  |
| 11.04.2015 | RAS | C |  | 6.79 | 26.6 | 1.1 |
| 11.04.2015 | RAS | C | 5.5 | 6.79 | 26.6 | 1.1 |
| 11.04.2015 | RAS | C | 7.9 | 6.79 | 26.6 | 1.1 |
| 11.04.2015 | RAS | C | 4.65 | 6.79 | 26.6 | 1.1 |
| 11.04.2015 | RAS | D |  | 7.39 | 27 | 1.1 |
| 11.04.2015 | RAS | D |  | 7.39 | 27 | 1.1 |
| 11.04.2015 | RAS | D | 3.93 | 7.39 | 27 | 1.1 |
| 11.04.2015 | RAS | D | 7.5 | 7.39 | 27 | 1.1 |
| 13.04.2015 | Hydro | D | 8.99 | 7.33 | 20.8 | 1.41 |
| 13.04.2015 | RAS | A | 6.5 | 7.04 | 26.6 |  |
| 13.04.2015 | RAS | A | 6 | 7.04 | 26.6 |  |
| 13.04.2015 | RAS | A | 7.1 | 7.04 | 26.6 |  |
| 13.04.2015 | RAS | A | 5.2 | 7.04 | 26.6 |  |
| 13.04.2015 | RAS | C |  | 6.71 | 26.3 | 1.1 |
| 13.04.2015 | RAS | C | 6.8 | 6.71 | 26.3 | 1.1 |
| 13.04.2015 | RAS | C | 8.1 | 6.71 | 26.3 | 1.1 |
| 13.04.2015 | RAS | C | 4.6 | 6.71 | 26.3 | 1.1 |
| 13.04.2015 | RAS | D | 7.2 | 7.37 | 26.7 | 1.1 |
| 13.04.2015 | RAS | D |  | 7.37 | 26.7 | 1.1 |
| 13.04.2015 | RAS | D | 4.9 | 7.37 | 26.7 | 1.1 |
| 13.04.2015 | RAS | D | 7.6 | 7.37 | 26.7 | 1.1 |
| 14.04.2015 | Hydro | D | 8.58 | 7.42 | 21.5 | 1.44 |
| 14.04.2015 | RAS | A | 6.4 | 6.82 | 26.1 | 0.9 |
| 14.04.2015 | RAS | A | 6 | 6.82 | 26.1 | 0.9 |
| 14.04.2015 | RAS | A | 7 | 6.82 | 26.1 | 0.9 |
| 14.04.2015 | RAS | A | 4.8 | 6.82 | 26.1 | 0.9 |
| 14.04.2015 | RAS | C | 6.7 | 7.05 | 25.9 | 1.1 |
| 14.04.2015 | RAS | C | 6.6 | 7.05 | 25.9 | 1.1 |
| 14.04.2015 | RAS | C | 8.2 | 7.05 | 25.9 | 1.1 |
| 14.04.2015 | RAS | C |  | 7.05 | 25.9 | 1.1 |
| 14.04.2015 | RAS | D |  | 7.41 | 26.1 | 1.1 |
| 14.04.2015 | RAS | D |  | 7.41 | 26.1 | 1.1 |
| 14.04.2015 | RAS | D | 9 | 7.41 | 26.1 | 1.1 |
| 14.04.2015 | RAS | D | 7.7 | 7.41 | 26.1 | 1.1 |
| 15.04.2015 | Hydro | D | 8.51 | 7.21 | 22.8 | 1.92 |
| 15.04.2015 | RAS | A | 5.2 | 6.67 | 26.2 | 1.2 |
| 15.04.2015 | RAS | A | 5.6 | 6.67 | 26.2 | 1.2 |
| 15.04.2015 | RAS | A | 6.8 | 6.67 | 26.2 | 1.2 |
| 15.04.2015 | RAS | A |  | 6.67 | 26.2 | 1.2 |
| 15.04.2015 | RAS | C | 5.9 | 6.63 | 26.6 | 1.1 |
| 15.04.2015 | RAS | C | 5.8 | 6.63 | 26.6 | 1.1 |
| 15.04.2015 | RAS | C | 8 | 6.63 | 26.6 | 1.1 |
| 15.04.2015 | RAS | C |  | 6.63 | 26.6 | 1.1 |
| 15.04.2015 | RAS | D |  | 7.22 | 26.6 | 1.1 |
| 15.04.2015 | RAS | D |  | 7.22 | 26.6 | 1.1 |
| 15.04.2015 | RAS | D | 5.8 | 7.22 | 26.6 | 1.1 |
| 15.04.2015 | RAS | D | 7.6 | 7.22 | 26.6 | 1.1 |
| 16.04.2015 | Hydro | D | 8.44 | 7.26 | 23.3 | 2.49 |
| 16.04.2015 | RAS | A | 6.2 | 7.24 | 26.6 | 1.1 |
| 16.04.2015 | RAS | A | 6.1 | 7.24 | 26.6 | 1.1 |
| 16.04.2015 | RAS | A | 7.4 | 7.24 | 26.6 | 1.1 |
| 16.04.2015 | RAS | A | 4.8 | 7.24 | 26.6 | 1.1 |
| 16.04.2015 | RAS | C | 7.1 | 7.75 | 25.1 | 1 |
| 16.04.2015 | RAS | C | 6.8 | 7.75 | 25.1 | 1 |
| 16.04.2015 | RAS | C | 8.5 | 7.75 | 25.1 | 1 |
| 16.04.2015 | RAS | C | 7.6 | 7.75 | 25.1 | 1 |
| 16.04.2015 | RAS | D |  | 7.11 | 26.9 | 1.1 |
| 16.04.2015 | RAS | D |  | 7.11 | 26.9 | 1.1 |
| 16.04.2015 | RAS | D | 7.7 | 7.11 | 26.9 | 1.1 |
| 16.04.2015 | RAS | D | 7.6 | 7.11 | 26.9 | 1.1 |
| 17.04.2015 | Hydro | D | 8.55 | 5.52 | 21.9 | 3.85 |
| 17.04.2015 | RAS | A | 6.5 | 7.23 | 25.8 | 1.1 |
| 17.04.2015 | RAS | A | 6.3 | 7.23 | 25.8 | 1.1 |
| 17.04.2015 | RAS | A | 7.4 | 7.23 | 25.8 | 1.1 |
| 17.04.2015 | RAS | A | 5.7 | 7.23 | 25.8 | 1.1 |
| 17.04.2015 | RAS | C | 6.8 | 7.84 | 26 | 1 |
| 17.04.2015 | RAS | C | 6.6 | 7.84 | 26 | 1 |
| 17.04.2015 | RAS | C | 8.4 | 7.84 | 26 | 1 |
| 17.04.2015 | RAS | C | 6.2 | 7.84 | 26 | 1 |
| 17.04.2015 | RAS | D |  | 6.93 | 26 | 1.1 |
| 17.04.2015 | RAS | D | 8.7 | 6.93 | 26 | 1.1 |
| 17.04.2015 | RAS | D | 8.8 | 6.93 | 26 | 1.1 |
| 17.04.2015 | RAS | D | 7.7 | 6.93 | 26 | 1.1 |
| 18.04.2015 | Hydro | D | 8.54 | 5.28 | 22.3 | 2.83 |
| 18.04.2015 | RAS | A | 5.4 | 7.06 | 25.8 | 0.8 |
| 18.04.2015 | RAS | A | 6.1 | 7.06 | 25.8 | 0.8 |
| 18.04.2015 | RAS | A | 7.3 | 7.06 | 25.8 | 0.8 |
| 18.04.2015 | RAS | A | 9 | 7.06 | 25.8 | 0.8 |
| 18.04.2015 | RAS | C | 6.4 | 7.74 | 26.3 | 1 |
| 18.04.2015 | RAS | C | 5.8 | 7.74 | 26.3 | 1 |
| 18.04.2015 | RAS | C | 8.3 | 7.74 | 26.3 | 1 |
| 18.04.2015 | RAS | C |  | 7.74 | 26.3 | 1 |
| 18.04.2015 | RAS | D |  | 7.11 | 26.2 | 1.1 |
| 18.04.2015 | RAS | D |  | 7.11 | 26.2 | 1.1 |
| 18.04.2015 | RAS | D | 7.1 | 7.11 | 26.2 | 1.1 |
| 18.04.2015 | RAS | D |  | 7.11 | 26.2 | 1.1 |
| 19.04.2015 | Hydro | D | 8.79 | 5.92 | 21.4 | 3.1 |
| 19.04.2015 | RAS | A |  | 6.87 | 25.8 | 0.9 |
| 19.04.2015 | RAS | A |  | 6.87 | 25.8 | 0.9 |
| 19.04.2015 | RAS | A |  | 6.87 | 25.8 | 0.9 |
| 19.04.2015 | RAS | A |  | 6.87 | 25.8 | 0.9 |
| 19.04.2015 | RAS | C |  | 7.59 | 26 | 1 |
| 19.04.2015 | RAS | C |  | 7.59 | 26 | 1 |
| 19.04.2015 | RAS | C |  | 7.59 | 26 | 1 |
| 19.04.2015 | RAS | C |  | 7.59 | 26 | 1 |
| 19.04.2015 | RAS | D |  | 6.83 | 26.1 | 1.1 |
| 19.04.2015 | RAS | D |  | 6.83 | 26.1 | 1.1 |
| 19.04.2015 | RAS | D | 5.7 | 6.83 | 26.1 | 1.1 |
| 19.04.2015 | RAS | D |  | 6.83 | 26.1 | 1.1 |
| 20.04.2015 | Hydro | D | 8.78 | 6.3 | 21.8 | 3.3 |
| 20.04.2015 | RAS | A | 4.8 | 6.63 | 25.1 | 0.9 |
| 20.04.2015 | RAS | A | 4.2 | 6.63 | 25.1 | 0.9 |
| 20.04.2015 | RAS | A | 7 | 6.63 | 25.1 | 0.9 |
| 20.04.2015 | RAS | A | 4.7 | 6.63 | 25.1 | 0.9 |
| 20.04.2015 | RAS | C | 6.4 | 7.58 | 25.8 | 1 |
| 20.04.2015 | RAS | C | 4.5 | 7.58 | 25.8 | 1 |
| 20.04.2015 | RAS | C | 8.5 | 7.58 | 25.8 | 1 |
| 20.04.2015 | RAS | C | 5.3 | 7.58 | 25.8 | 1 |
| 20.04.2015 | RAS | D |  | 6.51 | 26.2 | 1.2 |
| 20.04.2015 | RAS | D |  | 6.51 | 26.2 | 1.2 |
| 20.04.2015 | RAS | D | 5.4 | 6.51 | 26.2 | 1.2 |
| 20.04.2015 | RAS | D | 7.7 | 6.51 | 26.2 | 1.2 |
| 21.04.2015 | Hydro | D | 8.6 | 6 | 22.2 | 4.4 |
| 21.04.2015 | RAS | A | 6 | 7.2 | 25.8 | 1 |
| 21.04.2015 | RAS | A | 5.7 | 7.2 | 25.8 | 1 |
| 21.04.2015 | RAS | A | 7.2 | 7.2 | 25.8 | 1 |
| 21.04.2015 | RAS | A | 4.5 | 7.2 | 25.8 | 1 |
| 21.04.2015 | RAS | C | 7.4 | 7.5 | 26.1 | 1 |
| 21.04.2015 | RAS | C | 6 | 7.5 | 26.1 | 1 |
| 21.04.2015 | RAS | C | 8.5 | 7.5 | 26.1 | 1 |
| 21.04.2015 | RAS | C | 7.2 | 7.5 | 26.1 | 1 |
| 21.04.2015 | RAS | D |  | 6.8 | 26.1 | 1.1 |
| 21.04.2015 | RAS | D |  | 6.8 | 26.1 | 1.1 |
| 21.04.2015 | RAS | D | 7.3 | 6.8 | 26.1 | 1.1 |
| 21.04.2015 | RAS | D | 7.7 | 6.8 | 26.1 | 1.1 |
| 22.04.2015 | Hydro | D | 8.2 | 4.66 | 23.5 | 3.6 |
| 22.04.2015 | RAS | A | 5.8 | 7.05 | 26.3 | 1 |
| 22.04.2015 | RAS | A | 5.7 | 7.05 | 26.3 | 1 |
| 22.04.2015 | RAS | A | 7.3 | 7.05 | 26.3 | 1 |
| 22.04.2015 | RAS | A | 4 | 7.05 | 26.3 | 1 |
| 22.04.2015 | RAS | C | 6.8 | 7.45 | 25.9 | 1.1 |
| 22.04.2015 | RAS | C |  | 7.45 | 25.9 | 1.1 |
| 22.04.2015 | RAS | C | 8.7 | 7.45 | 25.9 | 1.1 |
| 22.04.2015 | RAS | C | 6.4 | 7.45 | 25.9 | 1.1 |
| 22.04.2015 | RAS | D |  | 7.11 | 26.7 | 1.2 |
| 22.04.2015 | RAS | D |  | 7.11 | 26.7 | 1.2 |
| 22.04.2015 | RAS | D | 7 | 7.11 | 26.7 | 1.2 |
| 22.04.2015 | RAS | D | 7.7 | 7.11 | 26.7 | 1.2 |
| 23.04.2015 | Hydro | D | 6.24 | 6.24 | 23.4 | 4.97 |
| 23.04.2015 | RAS | A | 5.7 | 6.77 | 26.1 | 0.8 |
| 23.04.2015 | RAS | A | 5.5 | 6.77 | 26.1 | 0.8 |
| 23.04.2015 | RAS | A | 7.2 | 6.77 | 26.1 | 0.8 |
| 23.04.2015 | RAS | A | 5.08 | 6.77 | 26.1 | 0.8 |
| 23.04.2015 | RAS | C | 6.1 | 7.34 | 26.3 | 1.1 |
| 23.04.2015 | RAS | C |  | 7.34 | 26.3 | 1.1 |
| 23.04.2015 | RAS | C | 8.9 | 7.34 | 26.3 | 1.1 |
| 23.04.2015 | RAS | C | 6 | 7.34 | 26.3 | 1.1 |
| 23.04.2015 | RAS | D |  | 6.87 | 26.5 | 1.2 |
| 23.04.2015 | RAS | D |  | 6.87 | 26.5 | 1.2 |
| 23.04.2015 | RAS | D | 6.9 | 6.87 | 26.5 | 1.2 |
| 23.04.2015 | RAS | D | 7.6 | 6.87 | 26.5 | 1.2 |
| 24.04.2015 | Hydro | D | 7.79 | 6.11 | 24 | 2.44 |
| 24.04.2015 | RAS | A | 7.7 | 6.45 | 26.5 | 1.1 |
| 24.04.2015 | RAS | A | 4.5 | 6.45 | 26.5 | 1.1 |
| 24.04.2015 | RAS | A | 7 | 6.45 | 26.5 | 1.1 |
| 24.04.2015 | RAS | A | 5.9 | 6.45 | 26.5 | 1.1 |
| 24.04.2015 | RAS | C | 5.7 | 7.03 | 26.5 | 1.1 |
| 24.04.2015 | RAS | C | 4.9 | 7.03 | 26.5 | 1.1 |
| 24.04.2015 | RAS | C | 8.5 | 7.03 | 26.5 | 1.1 |
| 24.04.2015 | RAS | C | 6.1 | 7.03 | 26.5 | 1.1 |
| 24.04.2015 | RAS | D | 8 | 6.31 | 26.6 | 1.3 |
| 24.04.2015 | RAS | D |  | 6.31 | 26.6 | 1.3 |
| 24.04.2015 | RAS | D | 5.1 | 6.31 | 26.6 | 1.3 |
| 24.04.2015 | RAS | D | 7.5 | 6.31 | 26.6 | 1.3 |
| 25.04.2015 | Hydro | D | 7.72 | 5.92 | 24.7 | 2.23 |
| 25.04.2015 | RAS | A | 7.4 | 7.19 | 26.6 | 1.1 |
| 25.04.2015 | RAS | A | 5 | 7.19 | 26.6 | 1.1 |
| 25.04.2015 | RAS | A | 7 | 7.19 | 26.6 | 1.1 |
| 25.04.2015 | RAS | A | 5 | 7.19 | 26.6 | 1.1 |
| 25.04.2015 | RAS | C | 6 | 6.98 | 26.7 | 1.1 |
| 25.04.2015 | RAS | C | 4.8 | 6.98 | 26.7 | 1.1 |
| 25.04.2015 | RAS | C | 8.5 | 6.98 | 26.7 | 1.1 |
| 25.04.2015 | RAS | C | 4.7 | 6.98 | 26.7 | 1.1 |
| 25.04.2015 | RAS | D | 8.6 | 7.24 | 26.8 | 1.3 |
| 25.04.2015 | RAS | D |  | 7.24 | 26.8 | 1.3 |
| 25.04.2015 | RAS | D | 5.4 | 7.24 | 26.8 | 1.3 |
| 25.04.2015 | RAS | D | 7.5 | 7.24 | 26.8 | 1.3 |
| 26.04.2015 | Hydro | D | 8.1 | 6.33 | 23.5 | 1.78 |
| 26.04.2015 | RAS | A | 7.6 | 7.82 | 26.5 |  |
| 26.04.2015 | RAS | A | 4.9 | 7.82 | 26.5 |  |
| 26.04.2015 | RAS | A | 7 | 7.82 | 26.5 |  |
| 26.04.2015 | RAS | A |  | 7.82 | 26.5 |  |
| 26.04.2015 | RAS | C | 6.5 | 6.37 | 26.3 | 1.1 |
| 26.04.2015 | RAS | C | 5.1 | 6.37 | 26.3 | 1.1 |
| 26.04.2015 | RAS | C | 8.5 | 6.37 | 26.3 | 1.1 |
| 26.04.2015 | RAS | C | 4.9 | 6.37 | 26.3 | 1.1 |
| 26.04.2015 | RAS | D | 7.6 | 6.82 | 28.2 | 1.3 |
| 26.04.2015 | RAS | D |  | 6.82 | 28.2 | 1.3 |
| 26.04.2015 | RAS | D | 5.9 | 6.82 | 28.2 | 1.3 |
| 26.04.2015 | RAS | D | 7.4 | 6.82 | 28.2 | 1.3 |
| 27.04.2015 | Hydro | D | 8.31 | 6.2 | 23.1 | 1.89 |
| 27.04.2015 | RAS | A | 7.6 | 6.98 | 26.6 |  |
| 27.04.2015 | RAS | A | 4.9 | 6.98 | 26.6 |  |
| 27.04.2015 | RAS | A | 6.9 | 6.98 | 26.6 |  |
| 27.04.2015 | RAS | A |  | 6.98 | 26.6 |  |
| 27.04.2015 | RAS | C | 6.5 | 5.84 | 25.9 | 1.1 |
| 27.04.2015 | RAS | C | 4.8 | 5.84 | 25.9 | 1.1 |
| 27.04.2015 | RAS | C | 8.7 | 5.84 | 25.9 | 1.1 |
| 27.04.2015 | RAS | C | 5.5 | 5.84 | 25.9 | 1.1 |
| 27.04.2015 | RAS | D | 7.7 | 6.56 | 27.1 | 1.3 |
| 27.04.2015 | RAS | D |  | 6.56 | 27.1 | 1.3 |
| 27.04.2015 | RAS | D | 7.3 | 6.56 | 27.1 | 1.3 |
| 27.04.2015 | RAS | D | 7.3 | 6.56 | 27.1 | 1.3 |
| 28.04.2015 | Hydro | D | 8.58 | 6.33 | 22.4 | 2.07 |
| 28.04.2015 | RAS | A | 7.7 | 7.29 | 26 | 0.8 |
| 28.04.2015 | RAS | A | 5.3 | 7.29 | 26 | 0.8 |
| 28.04.2015 | RAS | A | 7.1 | 7.29 | 26 | 0.8 |
| 28.04.2015 | RAS | A | 5 | 7.29 | 26 | 0.8 |
| 28.04.2015 | RAS | C | 6.6 | 7.23 | 26.2 | 1.1 |
| 28.04.2015 | RAS | C | 6.4 | 7.23 | 26.2 | 1.1 |
| 28.04.2015 | RAS | C | 8.4 | 7.23 | 26.2 | 1.1 |
| 28.04.2015 | RAS | C | 5.6 | 7.23 | 26.2 | 1.1 |
| 28.04.2015 | RAS | D | 8 | 6.87 | 26 | 1.3 |
| 28.04.2015 | RAS | D |  | 6.87 | 26 | 1.3 |
| 28.04.2015 | RAS | D | 4.52 | 6.87 | 26 | 1.3 |
| 28.04.2015 | RAS | D | 7.5 | 6.87 | 26 | 1.3 |
| 29.04.2015 | Hydro | D | 8.42 | 6.5 | 23.7 | 2.56 |
| 29.04.2015 | RAS | A | 7.7 | 7.13 | 26.1 | 0.8 |
| 29.04.2015 | RAS | A | 4.9 | 7.13 | 26.1 | 0.8 |
| 29.04.2015 | RAS | A | 7.2 | 7.13 | 26.1 | 0.8 |
| 29.04.2015 | RAS | A | 5.1 | 7.13 | 26.1 | 0.8 |
| 29.04.2015 | RAS | C | 5.9 | 6.8 | 26.4 | 1.2 |
| 29.04.2015 | RAS | C | 6 | 6.8 | 26.4 | 1.2 |
| 29.04.2015 | RAS | C | 7.5 | 6.8 | 26.4 | 1.2 |
| 29.04.2015 | RAS | C |  | 6.8 | 26.4 | 1.2 |
| 29.04.2015 | RAS | D | 7.5 | 7.15 | 26.4 | 1.3 |
| 29.04.2015 | RAS | D | 6.9 | 7.15 | 26.4 | 1.3 |
| 29.04.2015 | RAS | D | 5.25 | 7.15 | 26.4 | 1.3 |
| 29.04.2015 | RAS | D | 7.5 | 7.15 | 26.4 | 1.3 |
| 30.04.2015 | Hydro | D |  |  |  |  |
| 30.04.2015 | RAS | A | 7.7 | 7.32 | 25.8 | 0.8 |
| 30.04.2015 | RAS | A | 6.1 | 7.32 | 25.8 | 0.8 |
| 30.04.2015 | RAS | A | 7.6 | 7.32 | 25.8 | 0.8 |
| 30.04.2015 | RAS | A |  | 7.32 | 25.8 | 0.8 |
| 30.04.2015 | RAS | C | 7.9 | 7.09 | 26 | 1.2 |
| 30.04.2015 | RAS | C | 4 | 7.09 | 26 | 1.2 |
| 30.04.2015 | RAS | C | 7.7 | 7.09 | 26 | 1.2 |
| 30.04.2015 | RAS | C | 4.6 | 7.09 | 26 | 1.2 |
| 30.04.2015 | RAS | D |  | 7.83 | 24.6 | 1.1 |
| 30.04.2015 | RAS | D | 4.8 | 7.83 | 24.6 | 1.1 |
| 30.04.2015 | RAS | D | 6.6 | 7.83 | 24.6 | 1.1 |
| 30.04.2015 | RAS | D | 7.7 | 7.83 | 24.6 | 1.1 |
| 01.05.2015 | Hydro | D | 7.77 | 5.98 | 23.3 | 1.9 |
| 01.05.2015 | RAS | A |  | 7.34 | 26.2 |  |
| 01.05.2015 | RAS | A |  | 7.34 | 26.2 |  |
| 01.05.2015 | RAS | A | 7.4 | 7.34 | 26.2 |  |
| 01.05.2015 | RAS | A | 4.3 | 7.34 | 26.2 |  |
| 01.05.2015 | RAS | C |  | 7.24 | 26.3 | 1.2 |
| 01.05.2015 | RAS | C |  | 7.24 | 26.3 | 1.2 |
| 01.05.2015 | RAS | C | 7.6 | 7.24 | 26.3 | 1.2 |
| 01.05.2015 | RAS | C | 3.9 | 7.24 | 26.3 | 1.2 |
| 01.05.2015 | RAS | D |  | 7.73 | 26.1 | 1.2 |
| 01.05.2015 | RAS | D |  | 7.73 | 26.1 | 1.2 |
| 01.05.2015 | RAS | D | 4.4 | 7.73 | 26.1 | 1.2 |
| 01.05.2015 | RAS | D | 7.4 | 7.73 | 26.1 | 1.2 |
| 02.05.2015 | Hydro | D | 7.9 | 6.23 | 23.9 | 2.3 |
| 02.05.2015 | RAS | A | 7.7 | 7.33 | 26.4 |  |
| 02.05.2015 | RAS | A | 5.8 | 7.33 | 26.4 |  |
| 02.05.2015 | RAS | A | 7.3 | 7.33 | 26.4 |  |
| 02.05.2015 | RAS | A | 5.49 | 7.33 | 26.4 |  |
| 02.05.2015 | RAS | C | 7.5 | 7.12 | 26 | 1.2 |
| 02.05.2015 | RAS | C |  | 7.12 | 26 | 1.2 |
| 02.05.2015 | RAS | C | 7.8 | 7.12 | 26 | 1.2 |
| 02.05.2015 | RAS | C | 5.28 | 7.12 | 26 | 1.2 |
| 02.05.2015 | RAS | D | 6.2 | 7.69 | 26.6 | 1.2 |
| 02.05.2015 | RAS | D | 6.6 | 7.69 | 26.6 | 1.2 |
| 02.05.2015 | RAS | D | 5.6 | 7.69 | 26.6 | 1.2 |
| 02.05.2015 | RAS | D | 7.4 | 7.69 | 26.6 | 1.2 |
| 03.05.2015 | Hydro | D | 7.8 | 6.2 | 23.7 | 2.6 |
| 03.05.2015 | RAS | A | 7.6 | 7.4 | 24.4 |  |
| 03.05.2015 | RAS | A | 7.1 | 7.4 | 24.4 |  |
| 03.05.2015 | RAS | A | 7.7 | 7.4 | 24.4 |  |
| 03.05.2015 | RAS | A |  | 7.4 | 24.4 |  |
| 03.05.2015 | RAS | A | 7.4 | 6.93 | 27.3 | 0.9 |
| 03.05.2015 | RAS | C | 7.4 | 7.3 | 26.2 | 1.2 |
| 03.05.2015 | RAS | C |  | 7.3 | 26.2 | 1.2 |
| 03.05.2015 | RAS | C | 7.1 | 7.3 | 26.2 | 1.2 |
| 03.05.2015 | RAS | C |  | 7.3 | 26.2 | 1.2 |
| 03.05.2015 | RAS | D | 6.4 | 7.61 | 26.5 | 1.1 |
| 03.05.2015 | RAS | D | 6.7 | 7.61 | 26.5 | 1.1 |
| 03.05.2015 | RAS | D | 5.7 | 7.61 | 26.5 | 1.1 |
| 03.05.2015 | RAS | D |  | 7.61 | 26.5 | 1.1 |
| 04.05.2015 | Hydro | D | 7.82 | 6.62 | 25.1 | 2.75 |
| 04.05.2015 | RAS | A | 5.3 | 6.93 | 27.3 | 0.9 |
| 04.05.2015 | RAS | A | 7.1 | 6.93 | 27.3 | 0.9 |
| 04.05.2015 | RAS | A | 5.2 | 6.93 | 27.3 | 0.9 |
| 04.05.2015 | RAS | C | 8 | 6.49 | 26.7 | 1.3 |
| 04.05.2015 | RAS | C | 6.7 | 6.49 | 26.7 | 1.3 |
| 04.05.2015 | RAS | C | 7.8 | 6.49 | 26.7 | 1.3 |
| 04.05.2015 | RAS | C | 5.36 | 6.49 | 26.7 | 1.3 |
| 04.05.2015 | RAS | D | 5.6 | 7.43 | 27.6 | 1.2 |
| 04.05.2015 | RAS | D | 5.6 | 7.43 | 27.6 | 1.2 |
| 04.05.2015 | RAS | D | 5.5 | 7.43 | 27.6 | 1.2 |
| 04.05.2015 | RAS | D | 7.1 | 7.43 | 27.6 | 1.2 |
| 05.05.2015 | Hydro | D | 8.03 | 6.83 | 24.2 | 2.3 |
| 05.05.2015 | RAS | A | 7.4 | 7.03 | 27.7 |  |
| 05.05.2015 | RAS | A | 4.1 | 7.03 | 27.7 |  |
| 05.05.2015 | RAS | A | 6.9 | 7.03 | 27.7 |  |
| 05.05.2015 | RAS | A | 5.09 | 7.03 | 27.7 |  |
| 05.05.2015 | RAS | C | 7.7 | 6.67 | 26.2 | 1.2 |
| 05.05.2015 | RAS | C | 6.4 | 6.67 | 26.2 | 1.2 |
| 05.05.2015 | RAS | C | 8 | 6.67 | 26.2 | 1.2 |
| 05.05.2015 | RAS | C | 5.12 | 6.67 | 26.2 | 1.2 |
| 05.05.2015 | RAS | D | 4.5 | 7.27 | 27.3 | 1.1 |
| 05.05.2015 | RAS | D | 4.8 | 7.27 | 27.3 | 1.1 |
| 05.05.2015 | RAS | D | 5.22 | 7.27 | 27.3 | 1.1 |
| 05.05.2015 | RAS | D | 7.1 | 7.27 | 27.3 | 1.1 |
| 06.05.2015 | Hydro | D | 7.83 | 6.87 | 25.3 | 4.31 |
| 06.05.2015 | RAS | A | 7.7 | 7.45 | 25.1 | 1.1 |
| 06.05.2015 | RAS | A |  | 7.45 | 25.1 | 1.1 |
| 06.05.2015 | RAS | A | 5.9 | 7.45 | 25.1 | 1.1 |
| 06.05.2015 | RAS | A | 6.66 | 7.45 | 25.1 | 1.1 |
| 06.05.2015 | RAS | C | 8.9 | 7.35 | 25.7 | 1.2 |
| 06.05.2015 | RAS | C | 5.7 | 7.35 | 25.7 | 1.2 |
| 06.05.2015 | RAS | C | 6.9 | 7.35 | 25.7 | 1.2 |
| 06.05.2015 | RAS | C | 4.9 | 7.35 | 25.7 | 1.2 |
| 06.05.2015 | RAS | D | 4.1 | 7.41 | 26.1 | 1.1 |
| 06.05.2015 | RAS | D |  | 7.41 | 26.1 | 1.1 |
| 06.05.2015 | RAS | D | 6.04 | 7.41 | 26.1 | 1.1 |
| 06.05.2015 | RAS | D | 6 | 7.41 | 26.1 | 1.1 |
| 07.05.2015 | Hydro | D | 7.98 | 5.7 | 25.2 | 4.44 |
| 07.05.2015 | RAS | A | 7.6 | 7.43 | 26 | 1.1 |
| 07.05.2015 | RAS | A | 6 | 7.43 | 26 | 1.1 |
| 07.05.2015 | RAS | A | 5.9 | 7.43 | 26 | 1.1 |
| 07.05.2015 | RAS | A | 6.63 | 7.43 | 26 | 1.1 |
| 07.05.2015 | RAS | C | 8.7 | 7.25 | 26.4 | 1.2 |
| 07.05.2015 | RAS | C | 5.9 | 7.25 | 26.4 | 1.2 |
| 07.05.2015 | RAS | C | 6.8 | 7.25 | 26.4 | 1.2 |
| 07.05.2015 | RAS | C | 5.4 | 7.25 | 26.4 | 1.2 |
| 07.05.2015 | RAS | D |  | 7.36 | 26.5 | 1.1 |
| 07.05.2015 | RAS | D |  | 7.36 | 26.5 | 1.1 |
| 07.05.2015 | RAS | D | 5.71 | 7.36 | 26.5 | 1.1 |
| 07.05.2015 | RAS | D | 6.2 | 7.36 | 26.5 | 1.1 |
| 08.05.2015 | Hydro | D | 8.01 | 5.07 | 24 | 3.07 |
| 08.05.2015 | RAS | A | 7.6 | 7.1 | 26.3 | 1.1 |
| 08.05.2015 | RAS | A |  | 7.1 | 26.3 | 1.1 |
| 08.05.2015 | RAS | A | 5.4 | 7.1 | 26.3 | 1.1 |
| 08.05.2015 | RAS | A | 5.65 | 7.1 | 26.3 | 1.1 |
| 08.05.2015 | RAS | C | 8.5 | 6.67 | 26.1 | 1.2 |
| 08.05.2015 | RAS | C | 5.9 | 6.67 | 26.1 | 1.2 |
| 08.05.2015 | RAS | C | 6.9 | 6.67 | 26.1 | 1.2 |
| 08.05.2015 | RAS | C | 6.2 | 6.67 | 26.1 | 1.2 |
| 08.05.2015 | RAS | D |  | 6.85 | 26.8 | 1.1 |
| 08.05.2015 | RAS | D |  | 6.85 | 26.8 | 1.1 |
| 08.05.2015 | RAS | D | 5 | 6.85 | 26.8 | 1.1 |
| 08.05.2015 | RAS | D | 5.9 | 6.85 | 26.8 | 1.1 |
| 09.05.2015 | Hydro | D | 8.07 | 5.09 | 23.8 | 3.39 |
| 09.05.2015 | RAS | A | 6.3 | 7.67 | 25.9 | 1 |
| 09.05.2015 | RAS | A | 7.7 | 7.67 | 25.9 | 1 |
| 09.05.2015 | RAS | A | 5.5 | 7.67 | 25.9 | 1 |
| 09.05.2015 | RAS | A | 5.4 | 7.67 | 25.9 | 1 |
| 09.05.2015 | RAS | C | 8.4 | 7.49 | 26.2 | 1.2 |
| 09.05.2015 | RAS | C | 7.1 | 7.49 | 26.2 | 1.2 |
| 09.05.2015 | RAS | C | 6.7 | 7.49 | 26.2 | 1.2 |
| 09.05.2015 | RAS | C | 6 | 7.49 | 26.2 | 1.2 |
| 09.05.2015 | RAS | D | 6 | 7.95 | 26.4 | 1.2 |
| 09.05.2015 | RAS | D | 8.9 | 7.95 | 26.4 | 1.2 |
| 09.05.2015 | RAS | D | 7.1 | 7.95 | 26.4 | 1.2 |
| 09.05.2015 | RAS | D | 6.1 | 7.95 | 26.4 | 1.2 |
| 10.05.2015 | Hydro | D | 8.22 | 5.24 | 23.6 | 3.6 |
| 10.05.2015 | RAS | A | 6.6 | 7.4 | 26.1 |  |
| 10.05.2015 | RAS | A | 7.7 | 7.4 | 26.1 |  |
| 10.05.2015 | RAS | A | 6.1 | 7.4 | 26.1 |  |
| 10.05.2015 | RAS | A |  | 7.4 | 26.1 |  |
| 10.05.2015 | RAS | C |  | 7.46 | 26.1 | 1.2 |
| 10.05.2015 | RAS | C | 7.4 | 7.46 | 26.1 | 1.2 |
| 10.05.2015 | RAS | C | 7.3 | 7.46 | 26.1 | 1.2 |
| 10.05.2015 | RAS | C | 7 | 7.46 | 26.1 | 1.2 |
| 10.05.2015 | RAS | D | 6.6 | 7.34 | 26.6 | 1.2 |
| 10.05.2015 | RAS | D | 8.7 | 7.34 | 26.6 | 1.2 |
| 10.05.2015 | RAS | D | 6.6 | 7.34 | 26.6 | 1.2 |
| 10.05.2015 | RAS | D | 6.5 | 7.34 | 26.6 | 1.2 |
| 11.05.2015 | Hydro | D | 8.46 | 5.38 | 23.3 | 3.88 |
| 11.05.2015 | RAS | A | 7.1 | 8.17 | 25.7 | 1.018 |
| 11.05.2015 | RAS | A | 7.7 | 8.17 | 25.7 | 1.018 |
| 11.05.2015 | RAS | A | 5.7 | 8.17 | 25.7 | 1.018 |
| 11.05.2015 | RAS | A | 6.8 | 8.17 | 25.7 | 1.018 |
| 11.05.2015 | RAS | C | 8 | 7.49 | 26.2 | 1.2 |
| 11.05.2015 | RAS | C | 7.5 | 7.49 | 26.2 | 1.2 |
| 11.05.2015 | RAS | C | 7.1 | 7.49 | 26.2 | 1.2 |
| 11.05.2015 | RAS | C | 6.7 | 7.49 | 26.2 | 1.2 |
| 11.05.2015 | RAS | D | 6.3 | 8.03 | 26.5 | 1.2 |
| 11.05.2015 | RAS | D | 8.7 | 8.03 | 26.5 | 1.2 |
| 11.05.2015 | RAS | D | 4.7 | 8.03 | 26.5 | 1.2 |
| 11.05.2015 | RAS | D | 6.94 | 8.03 | 26.5 | 1.2 |
| 12.05.2015 | Hydro | D | 8.16 | 5.48 | 24.2 | 4.58 |
| 12.05.2015 | RAS | A | 7.5 | 8 | 26.5 | 1 |
| 12.05.2015 | RAS | A | 7.4 | 8 | 26.5 | 1 |
| 12.05.2015 | RAS | A | 5.3 | 8 | 26.5 | 1 |
| 12.05.2015 | RAS | A | 6.29 | 8 | 26.5 | 1 |
| 12.05.2015 | RAS | C | 7.6 | 7.44 | 25.9 | 1.2 |
| 12.05.2015 | RAS | C | 7.2 | 7.44 | 25.9 | 1.2 |
| 12.05.2015 | RAS | C | 6.6 | 7.44 | 25.9 | 1.2 |
| 12.05.2015 | RAS | C | 5.9 | 7.44 | 25.9 | 1.2 |
| 12.05.2015 | RAS | D | 6.71 | 7.9 | 26.6 | 1.2 |
| 12.05.2015 | RAS | D | 8.4 | 7.9 | 26.6 | 1.2 |
| 12.05.2015 | RAS | D | 6.25 | 7.9 | 26.6 | 1.2 |
| 12.05.2015 | RAS | D | 6.65 | 7.9 | 26.6 | 1.2 |
| 13.05.2015 | Hydro | D | 8.3 | 5.98 | 23 | 5.1 |
| 13.05.2015 | RAS | A | 7.8 | 8.46 | 25.9 |  |
| 13.05.2015 | RAS | A | 7.5 | 8.46 | 25.9 |  |
| 13.05.2015 | RAS | A | 5.3 | 8.46 | 25.9 |  |
| 13.05.2015 | RAS | A | 6.36 | 8.46 | 25.9 |  |
| 13.05.2015 | RAS | C | 7.7 | 7.41 | 26.3 | 1.2 |
| 13.05.2015 | RAS | C | 8.2 | 7.41 | 26.3 | 1.2 |
| 13.05.2015 | RAS | C | 6.6 | 7.41 | 26.3 | 1.2 |
| 13.05.2015 | RAS | C | 5.8 | 7.41 | 26.3 | 1.2 |
| 13.05.2015 | RAS | D | 5.9 | 8.08 | 26.5 | 1.2 |
| 13.05.2015 | RAS | D | 8.3 | 8.08 | 26.5 | 1.2 |
| 13.05.2015 | RAS | D | 6.3 | 8.08 | 26.5 | 1.2 |
| 13.05.2015 | RAS | D | 6.73 | 8.08 | 26.5 | 1.2 |
| 14.05.2015 | Hydro | D | 8.17 | 5.05 | 23.6 | 2.39 |
| 14.05.2015 | RAS | A | 6.2 | 7.23 | 25.7 |  |
| 14.05.2015 | RAS | A | 7.2 | 7.23 | 25.7 |  |
| 14.05.2015 | RAS | A | 5.8 | 7.23 | 25.7 |  |
| 14.05.2015 | RAS | A |  | 7.23 | 25.7 |  |
| 14.05.2015 | RAS | C |  | 6.87 | 26.2 | 1.2 |
| 14.05.2015 | RAS | C | 7.7 | 6.87 | 26.2 | 1.2 |
| 14.05.2015 | RAS | C | 6.5 | 6.87 | 26.2 | 1.2 |
| 14.05.2015 | RAS | C | 6.1 | 6.87 | 26.2 | 1.2 |
| 14.05.2015 | RAS | D | 7.8 | 7.09 | 26.4 | 1.2 |
| 14.05.2015 | RAS | D | 7.3 | 7.09 | 26.4 | 1.2 |
| 14.05.2015 | RAS | D |  | 7.09 | 26.4 | 1.2 |
| 14.05.2015 | RAS | D |  | 7.09 | 26.4 | 1.2 |
| 15.05.2015 | Hydro | D | 8.44 | 4.85 | 22.9 | 2.56 |
| 15.05.2015 | RAS | A | 6 | 7.2 | 25.5 | 1 |
| 15.05.2015 | RAS | A | 7.2 | 7.2 | 25.5 | 1 |
| 15.05.2015 | RAS | A | 5.7 | 7.2 | 25.5 | 1 |
| 15.05.2015 | RAS | A | 6.46 | 7.2 | 25.5 | 1 |
| 15.05.2015 | RAS | C |  | 6.38 | 26.1 | 1.2 |
| 15.05.2015 | RAS | C | 7.7 | 6.38 | 26.1 | 1.2 |
| 15.05.2015 | RAS | C | 6.7 | 6.38 | 26.1 | 1.2 |
| 15.05.2015 | RAS | C | 5.8 | 6.38 | 26.1 | 1.2 |
| 15.05.2015 | RAS | D | 7.3 | 7.12 | 26.3 | 1.2 |
| 15.05.2015 | RAS | D | 7.1 | 7.12 | 26.3 | 1.2 |
| 15.05.2015 | RAS | D | 6.39 | 7.12 | 26.3 | 1.2 |
| 15.05.2015 | RAS | D | 6.74 | 7.12 | 26.3 | 1.2 |
| 16.05.2015 | Hydro | D | 8.18 | 5.59 | 22.5 | 1.9 |
| 16.05.2015 | RAS | A | 6.1 | 7.14 | 25.4 |  |
| 16.05.2015 | RAS | A | 7.4 | 7.14 | 25.4 |  |
| 16.05.2015 | RAS | A | 5.5 | 7.14 | 25.4 |  |
| 16.05.2015 | RAS | A | 6.24 | 7.14 | 25.4 |  |
| 16.05.2015 | RAS | C | 7.8 | 7.32 | 26.2 | 1.2 |
| 16.05.2015 | RAS | C | 7.7 | 7.32 | 26.2 | 1.2 |
| 16.05.2015 | RAS | C | 6.7 | 7.32 | 26.2 | 1.2 |
| 16.05.2015 | RAS | C | 5.8 | 7.32 | 26.2 | 1.2 |
| 16.05.2015 | RAS | D | 7.3 | 7.01 | 26.1 | 1.2 |
| 16.05.2015 | RAS | D | 7.1 | 7.01 | 26.1 | 1.2 |
| 16.05.2015 | RAS | D | 6.39 | 7.01 | 26.1 | 1.2 |
| 16.05.2015 | RAS | D | 6.89 | 7.01 | 26.1 | 1.2 |
| 17.05.2015 | Hydro | D | 8.4 | 5.64 | 22.3 | 2 |
| 17.05.2015 | RAS | A | 7.7 | 7.13 | 25.4 |  |
| 17.05.2015 | RAS | A | 7.3 | 7.13 | 25.4 |  |
| 17.05.2015 | RAS | A | 5.6 | 7.13 | 25.4 |  |
| 17.05.2015 | RAS | A |  | 7.13 | 25.4 |  |
| 17.05.2015 | RAS | C | 7.7 | 7.16 | 26.1 | 1.2 |
| 17.05.2015 | RAS | C | 7.6 | 7.16 | 26.1 | 1.2 |
| 17.05.2015 | RAS | C | 6.9 | 7.16 | 26.1 | 1.2 |
| 17.05.2015 | RAS | C |  | 7.16 | 26.1 | 1.2 |
| 17.05.2015 | RAS | D | 7.7 | 7.04 | 26.1 | 1.2 |
| 17.05.2015 | RAS | D | 7.1 | 7.04 | 26.1 | 1.2 |
| 17.05.2015 | RAS | D |  | 7.04 | 26.1 | 1.2 |
| 17.05.2015 | RAS | D |  | 7.04 | 26.1 | 1.2 |
| 18.05.2015 | Hydro | D | 8.27 | 6.11 | 23 | 3.93 |
| 18.05.2015 | RAS | A | 7.6 | 7.18 | 25.4 | 1 |
| 18.05.2015 | RAS | A | 7.2 | 7.18 | 25.4 | 1 |
| 18.05.2015 | RAS | A | 5.5 | 7.18 | 25.4 | 1 |
| 18.05.2015 | RAS | A | 6.38 | 7.18 | 25.4 | 1 |
| 18.05.2015 | RAS | C | 7.2 | 7.04 | 26.2 | 1.2 |
| 18.05.2015 | RAS | C | 7.4 | 7.04 | 26.2 | 1.2 |
| 18.05.2015 | RAS | C | 6.7 | 7.04 | 26.2 | 1.2 |
| 18.05.2015 | RAS | C | 5.5 | 7.04 | 26.2 | 1.2 |
| 18.05.2015 | RAS | D | 7.6 | 7.26 | 26.2 | 1.2 |
| 18.05.2015 | RAS | D | 6.9 | 7.26 | 26.2 | 1.2 |
| 18.05.2015 | RAS | D | 6.33 | 7.26 | 26.2 | 1.2 |
| 18.05.2015 | RAS | D | 6.67 | 7.26 | 26.2 | 1.2 |
| 19.05.2015 | Hydro | D | 7.97 | 4.72 | 23.7 | 3.2 |
| 19.05.2015 | RAS | A | 7.4 | 7.06 | 25.8 | 1 |
| 19.05.2015 | RAS | A | 7.2 | 7.06 | 25.8 | 1 |
| 19.05.2015 | RAS | A | 5.1 | 7.06 | 25.8 | 1 |
| 19.05.2015 | RAS | A | 5.5 | 7.06 | 25.8 | 1 |
| 19.05.2015 | RAS | C | 7.2 | 7.17 | 25.9 | 1.2 |
| 19.05.2015 | RAS | C | 7.2 | 7.17 | 25.9 | 1.2 |
| 19.05.2015 | RAS | C | 6.4 | 7.17 | 25.9 | 1.2 |
| 19.05.2015 | RAS | C | 5.4 | 7.17 | 25.9 | 1.2 |
| 19.05.2015 | RAS | D | 7.4 | 7.45 | 26.2 | 1.2 |
| 19.05.2015 | RAS | D | 6.8 | 7.45 | 26.2 | 1.2 |
| 19.05.2015 | RAS | D | 5.3 | 7.45 | 26.2 | 1.2 |
| 19.05.2015 | RAS | D | 5.9 | 7.45 | 26.2 | 1.2 |
| 20.05.2015 | Hydro | D | 8.24 | 6.55 | 23.2 | 3.89 |
| 20.05.2015 | RAS | A | 7.5 | 6.72 | 26 | 1 |
| 20.05.2015 | RAS | A | 7.1 | 6.72 | 26 | 1 |
| 20.05.2015 | RAS | A | 5.5 | 6.72 | 26 | 1 |
| 20.05.2015 | RAS | A | 5.7 | 6.72 | 26 | 1 |
| 20.05.2015 | RAS | C | 6.3 | 6.77 | 26.3 | 1.2 |
| 20.05.2015 | RAS | C | 7.4 | 6.77 | 26.3 | 1.2 |
| 20.05.2015 | RAS | C | 4.4 | 6.77 | 26.3 | 1.2 |
| 20.05.2015 | RAS | C | 5.5 | 6.77 | 26.3 | 1.2 |
| 20.05.2015 | RAS | D | 6.3 | 7.17 | 26.3 | 1.2 |
| 20.05.2015 | RAS | D | 6.9 | 7.17 | 26.3 | 1.2 |
| 20.05.2015 | RAS | D | 5.5 | 7.17 | 26.3 | 1.2 |
| 20.05.2015 | RAS | D | 6.1 | 7.17 | 26.3 | 1.2 |
| 21.05.2015 | Hydro | D | 8.4 | 6.6 | 22.6 | 4.3 |
| 21.05.2015 | RAS | A | 7.6 | 7.07 | 25.7 | 1 |
| 21.05.2015 | RAS | A | 7.2 | 7.07 | 25.7 | 1 |
| 21.05.2015 | RAS | A | 6 | 7.07 | 25.7 | 1 |
| 21.05.2015 | RAS | A | 6.5 | 7.07 | 25.7 | 1 |
| 21.05.2015 | RAS | C | 7 | 7.03 | 26.2 | 1.3 |
| 21.05.2015 | RAS | C | 7.7 | 7.03 | 26.2 | 1.3 |
| 21.05.2015 | RAS | C | 6.4 | 7.03 | 26.2 | 1.3 |
| 21.05.2015 | RAS | C | 6.1 | 7.03 | 26.2 | 1.3 |
| 21.05.2015 | RAS | D | 6.9 | 7.08 | 26.1 | 1.2 |
| 21.05.2015 | RAS | D | 7 | 7.08 | 26.1 | 1.2 |
| 21.05.2015 | RAS | D | 6.7 | 7.08 | 26.1 | 1.2 |
| 21.05.2015 | RAS | D | 6.8 | 7.08 | 26.1 | 1.2 |
| 22.05.2015 | Hydro | D | 7.9 | 5.8 | 23.7 | 1.8 |
| 22.05.2015 | RAS | A | 7.9 | 7.31 | 24 | 1 |
| 22.05.2015 | RAS | A | 8.5 | 7.31 | 24 | 1 |
| 22.05.2015 | RAS | A | 5.5 | 7.31 | 24 | 1 |
| 22.05.2015 | RAS | A | 5.5 | 7.31 | 24 | 1 |
| 22.05.2015 | RAS | C | 6.2 | 6.99 | 26.3 | 1.3 |
| 22.05.2015 | RAS | C | 7.6 | 6.99 | 26.3 | 1.3 |
| 22.05.2015 | RAS | C | 5.8 | 6.99 | 26.3 | 1.3 |
| 22.05.2015 | RAS | C | 5.5 | 6.99 | 26.3 | 1.3 |
| 22.05.2015 | RAS | D | 6.3 | 7.25 | 24.7 | 1.2 |
| 22.05.2015 | RAS | D | 7.2 | 7.25 | 24.7 | 1.2 |
| 22.05.2015 | RAS | D | 5.6 | 7.25 | 24.7 | 1.2 |
| 22.05.2015 | RAS | D | 5.7 | 7.25 | 24.7 | 1.2 |
| 23.05.2015 | Hydro | D | 7.74 | 5.48 | 23.9 | 1.774 |
| 23.05.2015 | RAS | A | 7.7 | 7.49 | 25.2 |  |
| 23.05.2015 | RAS | A | 7.5 | 7.49 | 25.2 |  |
| 23.05.2015 | RAS | A | 5.9 | 7.49 | 25.2 |  |
| 23.05.2015 | RAS | A |  | 7.49 | 25.2 |  |
| 23.05.2015 | RAS | C | 6.6 | 7.09 | 26.2 | 1.3 |
| 23.05.2015 | RAS | C | 7.5 | 7.09 | 26.2 | 1.3 |
| 23.05.2015 | RAS | C | 5.3 | 7.09 | 26.2 | 1.3 |
| 23.05.2015 | RAS | C | 6 | 7.09 | 26.2 | 1.3 |
| 23.05.2015 | RAS | D | 6.5 | 7.35 | 26.3 | 1.2 |
| 23.05.2015 | RAS | D | 6.9 | 7.35 | 26.3 | 1.2 |
| 23.05.2015 | RAS | D |  | 7.35 | 26.3 | 1.2 |
| 23.05.2015 | RAS | D |  | 7.35 | 26.3 | 1.2 |
| 24.05.2015 | Hydro | D | 8.1 | 5.69 | 23.1 | 1.809 |
| 24.05.2015 | RAS | A | 7.5 | 7.44 | 25.7 |  |
| 24.05.2015 | RAS | A | 7.2 | 7.44 | 25.7 |  |
| 24.05.2015 | RAS | A | 5.6 | 7.44 | 25.7 |  |
| 24.05.2015 | RAS | A |  | 7.44 | 25.7 |  |
| 24.05.2015 | RAS | C | 6.8 | 6.77 | 26.2 | 1.3 |
| 24.05.2015 | RAS | C | 7.6 | 6.77 | 26.2 | 1.3 |
| 24.05.2015 | RAS | C | 5.8 | 6.77 | 26.2 | 1.3 |
| 24.05.2015 | RAS | C | 5.4 | 6.77 | 26.2 | 1.3 |
| 24.05.2015 | RAS | D | 6.8 | 7.27 | 26.3 | 1.2 |
| 24.05.2015 | RAS | D | 6.9 | 7.27 | 26.3 | 1.2 |
| 24.05.2015 | RAS | D |  | 7.27 | 26.3 | 1.2 |
| 24.05.2015 | RAS | D |  | 7.27 | 26.3 | 1.2 |
| 25.05.2015 | Hydro | D | 8.05 | 5.53 | 23.6 | 2.006 |
| 25.05.2015 | RAS | A | 7.4 | 7.61 | 26 |  |
| 25.05.2015 | RAS | A | 7.2 | 7.61 | 26 |  |
| 25.05.2015 | RAS | A |  | 7.61 | 26 |  |
| 25.05.2015 | RAS | A |  | 7.61 | 26 |  |
| 25.05.2015 | RAS | C | 6.4 | 7.03 | 26.2 | 1.4 |
| 25.05.2015 | RAS | C | 7.5 | 7.03 | 26.2 | 1.4 |
| 25.05.2015 | RAS | C | 5.3 | 7.03 | 26.2 | 1.4 |
| 25.05.2015 | RAS | C |  | 7.03 | 26.2 | 1.4 |
| 25.05.2015 | RAS | D | 6.9 | 7.36 | 26.7 | 1.3 |
| 25.05.2015 | RAS | D | 6.8 | 7.36 | 26.7 | 1.3 |
| 25.05.2015 | RAS | D |  | 7.36 | 26.7 | 1.3 |
| 25.05.2015 | RAS | D |  | 7.36 | 26.7 | 1.3 |
| 26.05.2015 | Hydro | D | 8.24 | 6.33 | 23.7 | 2.22 |
| 26.05.2015 | RAS | A | 7.5 | 7.55 | 26.6 | 1.1 |
| 26.05.2015 | RAS | A | 7.2 | 7.55 | 26.6 | 1.1 |
| 26.05.2015 | RAS | A | 5.3 | 7.55 | 26.6 | 1.1 |
| 26.05.2015 | RAS | A | 6.55 | 7.55 | 26.6 | 1.1 |
| 26.05.2015 | RAS | C | 6.6 | 6.67 | 26.2 | 1.4 |
| 26.05.2015 | RAS | C | 7.6 | 6.67 | 26.2 | 1.4 |
| 26.05.2015 | RAS | C | 5.9 | 6.67 | 26.2 | 1.4 |
| 26.05.2015 | RAS | C | 5 | 6.67 | 26.2 | 1.4 |
| 26.05.2015 | RAS | D | 7 | 7.23 | 27.1 | 1.3 |
| 26.05.2015 | RAS | D | 6.6 | 7.23 | 27.1 | 1.3 |
| 26.05.2015 | RAS | D | 6.1 | 7.23 | 27.1 | 1.3 |
| 26.05.2015 | RAS | D | 6.48 | 7.23 | 27.1 | 1.3 |
| 27.05.2015 | Hydro | D | 8.38 | 6.53 | 23 | 2.5 |
| 27.05.2015 | RAS | A | 7.7 | 7.35 | 25.7 | 1.1 |
| 27.05.2015 | RAS | A | 7.3 | 7.35 | 25.7 | 1.1 |
| 27.05.2015 | RAS | A | 6.86 | 7.35 | 25.7 | 1.1 |
| 27.05.2015 | RAS | A | 6.74 | 7.35 | 25.7 | 1.1 |
| 27.05.2015 | RAS | C | 6.9 | 7.26 | 26.3 | 1.4 |
| 27.05.2015 | RAS | C | 7.7 | 7.26 | 26.3 | 1.4 |
| 27.05.2015 | RAS | C | 5.5 | 7.26 | 26.3 | 1.4 |
| 27.05.2015 | RAS | C | 6.61 | 7.26 | 26.3 | 1.4 |
| 27.05.2015 | RAS | D | 6.4 | 7.13 | 26 | 1.2 |
| 27.05.2015 | RAS | D | 6.7 | 7.13 | 26 | 1.2 |
| 27.05.2015 | RAS | D | 6.35 | 7.13 | 26 | 1.2 |
| 27.05.2015 | RAS | D | 6.72 | 7.13 | 26 | 1.2 |
| 28.05.2015 | Hydro | D | 8.15 | 5.6 | 23.6 | 2.76 |
| 28.05.2015 | RAS | A | 6.7 | 7.07 | 25.5 | 1.1 |
| 28.05.2015 | RAS | A | 8 | 7.07 | 25.5 | 1.1 |
| 28.05.2015 | RAS | A | 6.67 | 7.07 | 25.5 | 1.1 |
| 28.05.2015 | RAS | A | 5.1 | 7.07 | 25.5 | 1.1 |
| 28.05.2015 | RAS | C | 6.8 | 7.27 | 26.1 | 1.4 |
| 28.05.2015 | RAS | C | 8.1 | 7.27 | 26.1 | 1.4 |
| 28.05.2015 | RAS | C | 6 | 7.27 | 26.1 | 1.4 |
| 28.05.2015 | RAS | C | 6.2 | 7.27 | 26.1 | 1.4 |
| 28.05.2015 | RAS | D | 7.4 | 7.18 | 26.1 | 1.3 |
| 28.05.2015 | RAS | D | 8.2 | 7.18 | 26.1 | 1.3 |
| 28.05.2015 | RAS | D | 8.1 | 7.18 | 26.1 | 1.3 |
| 28.05.2015 | RAS | D | 7.2 | 7.18 | 26.1 | 1.3 |
| 29.05.2015 | Hydro | D | 8.37 | 5.93 | 22.9 | 3.45 |
| 29.05.2015 | RAS | A | 6.5 | 7.28 | 26 | 1.1 |
| 29.05.2015 | RAS | A | 7.8 | 7.28 | 26 | 1.1 |
| 29.05.2015 | RAS | A | 6.56 | 7.28 | 26 | 1.1 |
| 29.05.2015 | RAS | A | 6.2 | 7.28 | 26 | 1.1 |
| 29.05.2015 | RAS | C | 7.9 | 7.05 | 25.8 | 1.4 |
| 29.05.2015 | RAS | C | 8 | 7.05 | 25.8 | 1.4 |
| 29.05.2015 | RAS | C | 6 | 7.05 | 25.8 | 1.4 |
| 29.05.2015 | RAS | C | 6.4 | 7.05 | 25.8 | 1.4 |
| 29.05.2015 | RAS | D | 6.9 | 6.94 | 26.6 | 1.3 |
| 29.05.2015 | RAS | D | 8 | 6.94 | 26.6 | 1.3 |
| 29.05.2015 | RAS | D | 6.2 | 6.94 | 26.6 | 1.3 |
| 29.05.2015 | RAS | D | 6.6 | 6.94 | 26.6 | 1.3 |
| 30.05.2015 | Hydro | D | 8.34 | 6.9 | 22.8 | 3.52 |
| 30.05.2015 | RAS | A | 6.6 | 7.27 | 26 | 1.1 |
| 30.05.2015 | RAS | A | 7.8 | 7.27 | 26 | 1.1 |
| 30.05.2015 | RAS | A | 6.6 | 7.27 | 26 | 1.1 |
| 30.05.2015 | RAS | A | 5.5 | 7.27 | 26 | 1.1 |
| 30.05.2015 | RAS | C | 7.7 | 7.02 | 26 | 1.3 |
| 30.05.2015 | RAS | C | 8 | 7.02 | 26 | 1.3 |
| 30.05.2015 | RAS | C | 6.2 | 7.02 | 26 | 1.3 |
| 30.05.2015 | RAS | C | 6.4 | 7.02 | 26 | 1.3 |
| 30.05.2015 | RAS | D | 6.5 | 7.01 | 26.7 | 1.3 |
| 30.05.2015 | RAS | D | 8.1 | 7.01 | 26.7 | 1.3 |
| 30.05.2015 | RAS | D | 6.4 | 7.01 | 26.7 | 1.3 |
| 30.05.2015 | RAS | D | 6.4 | 7.01 | 26.7 | 1.3 |
| 31.05.2015 | Hydro | D | 8.18 | 6.44 | 22.3 | 1.575 |
| 31.05.2015 | RAS | A | 6.7 | 7.09 | 25.3 | 1.1 |
| 31.05.2015 | RAS | A | 8 | 7.09 | 25.3 | 1.1 |
| 31.05.2015 | RAS | A | 7.1 | 7.09 | 25.3 | 1.1 |
| 31.05.2015 | RAS | A | 6.1 | 7.09 | 25.3 | 1.1 |
| 31.05.2015 | RAS | C |  | 7.06 | 26.1 | 1.4 |
| 31.05.2015 | RAS | C | 8 | 7.06 | 26.1 | 1.4 |
| 31.05.2015 | RAS | C | 5.9 | 7.06 | 26.1 | 1.4 |
| 31.05.2015 | RAS | C |  | 7.06 | 26.1 | 1.4 |
| 31.05.2015 | RAS | D | 6.6 | 7.02 | 26 | 1.3 |
| 31.05.2015 | RAS | D | 8 | 7.02 | 26 | 1.3 |
| 31.05.2015 | RAS | D | 6.4 | 7.02 | 26 | 1.3 |
| 31.05.2015 | RAS | D | 6.6 | 7.02 | 26 | 1.3 |
| 01.06.2015 | Hydro | D | 8.02 | 6.85 | 24.4 | 1.7 |
| 01.06.2015 | RAS | A | 7.6 | 7.13 | 26.6 | 1.1 |
| 01.06.2015 | RAS | A | 7.7 | 7.13 | 26.6 | 1.1 |
| 01.06.2015 | RAS | A | 6.15 | 7.13 | 26.6 | 1.1 |
| 01.06.2015 | RAS | A | 5.4 | 7.13 | 26.6 | 1.1 |
| 01.06.2015 | RAS | C | 6.6 | 7.17 | 26.3 | 1.5 |
| 01.06.2015 | RAS | C | 7.9 | 7.17 | 26.3 | 1.5 |
| 01.06.2015 | RAS | C | 5.2 | 7.17 | 26.3 | 1.5 |
| 01.06.2015 | RAS | C | 5.8 | 7.17 | 26.3 | 1.5 |
| 01.06.2015 | RAS | D | 6.6 | 6.9 | 26.5 | 1.4 |
| 01.06.2015 | RAS | D | 7.8 | 6.9 | 26.5 | 1.4 |
| 01.06.2015 | RAS | D | 5.5 | 6.9 | 26.5 | 1.4 |
| 01.06.2015 | RAS | D | 5.8 | 6.9 | 26.5 | 1.4 |
| 02.06.2015 | Hydro | D | 8.3 | 6.84 | 23.4 | 1.9 |
| 02.06.2015 | RAS | A | 7.8 | 7.21 | 25.8 | 1.1 |
| 02.06.2015 | RAS | A | 8.3 | 7.21 | 25.8 | 1.1 |
| 02.06.2015 | RAS | A | 7.6 | 7.21 | 25.8 | 1.1 |
| 02.06.2015 | RAS | A | 5.5 | 7.21 | 25.8 | 1.1 |
| 02.06.2015 | RAS | C | 6.8 | 7.22 | 26.3 | 1.4 |
| 02.06.2015 | RAS | C | 8.1 | 7.22 | 26.3 | 1.4 |
| 02.06.2015 | RAS | C | 5.4 | 7.22 | 26.3 | 1.4 |
| 02.06.2015 | RAS | C | 5.9 | 7.22 | 26.3 | 1.4 |
| 02.06.2015 | RAS | D | 6.4 | 7.85 | 26.1 | 1.3 |
| 02.06.2015 | RAS | D | 8.2 | 7.85 | 26.1 | 1.3 |
| 02.06.2015 | RAS | D | 5.7 | 7.85 | 26.1 | 1.3 |
| 02.06.2015 | RAS | D | 5.9 | 7.85 | 26.1 | 1.3 |
| 03.06.2015 | Hydro | D | 7.8 | 7.1 | 25.7 | 2.4 |
| 03.06.2015 | RAS | A | 7.7 | 7.33 | 26.6 | 1.1 |
| 03.06.2015 | RAS | A | 7.7 | 7.33 | 26.6 | 1.1 |
| 03.06.2015 | RAS | A | 6.1 | 7.33 | 26.6 | 1.1 |
| 03.06.2015 | RAS | A | 5.1 | 7.33 | 26.6 | 1.1 |
| 03.06.2015 | RAS | C | 6.4 | 6.91 | 26.7 | 1.4 |
| 03.06.2015 | RAS | C | 7.9 | 6.91 | 26.7 | 1.4 |
| 03.06.2015 | RAS | C |  | 6.91 | 26.7 | 1.4 |
| 03.06.2015 | RAS | C | 5.5 | 6.91 | 26.7 | 1.4 |
| 03.06.2015 | RAS | D | 6.3 | 6.92 | 27.1 | 1.4 |
| 03.06.2015 | RAS | D | 7.9 | 6.92 | 27.1 | 1.4 |
| 03.06.2015 | RAS | D | 5.6 | 6.92 | 27.1 | 1.4 |
| 03.06.2015 | RAS | D | 5.6 | 6.92 | 27.1 | 1.4 |
| 04.06.2015 | Hydro | D | 7.86 | 6.62 | 26.5 | 1.7 |
| 04.06.2015 | RAS | A | 7.6 | 6.98 | 26.5 | 1.1 |
| 04.06.2015 | RAS | A | 7.7 | 6.98 | 26.5 | 1.1 |
| 04.06.2015 | RAS | A |  | 6.98 | 26.5 | 1.1 |
| 04.06.2015 | RAS | A | 5.4 | 6.98 | 26.5 | 1.1 |
| 04.06.2015 | RAS | C | 6.4 | 7.05 | 26.3 | 1.5 |
| 04.06.2015 | RAS | C | 8.6 | 7.05 | 26.3 | 1.5 |
| 04.06.2015 | RAS | C | 5.1 | 7.05 | 26.3 | 1.5 |
| 04.06.2015 | RAS | C | 5.8 | 7.05 | 26.3 | 1.5 |
| 04.06.2015 | RAS | D | 6.5 | 7.03 | 26.9 | 1.4 |
| 04.06.2015 | RAS | D | 7.7 | 7.03 | 26.9 | 1.4 |
| 04.06.2015 | RAS | D | 5.4 | 7.03 | 26.9 | 1.4 |
| 04.06.2015 | RAS | D | 5.6 | 7.03 | 26.9 | 1.4 |
| 05.06.2015 | Hydro | D | 7.7 | 4.7 | 25 | 1.97 |
| 05.06.2015 | RAS | A | 6.2 | 7.42 | 26.4 | 1.1 |
| 05.06.2015 | RAS | A | 7.8 | 7.42 | 26.4 | 1.1 |
| 05.06.2015 | RAS | A |  | 7.42 | 26.4 | 1.1 |
| 05.06.2015 | RAS | A | 5.3 | 7.42 | 26.4 | 1.1 |
| 05.06.2015 | RAS | C | 5.7 | 7.02 | 26.5 | 1.5 |
| 05.06.2015 | RAS | C | 8 | 7.02 | 26.5 | 1.5 |
| 05.06.2015 | RAS | C | 4.3 | 7.02 | 26.5 | 1.5 |
| 05.06.2015 | RAS | C | 5.8 | 7.02 | 26.5 | 1.5 |
| 05.06.2015 | RAS | D | 6.3 | 7.08 | 27.1 | 1.4 |
| 05.06.2015 | RAS | D | 8 | 7.08 | 27.1 | 1.4 |
| 05.06.2015 | RAS | D | 5.6 | 7.08 | 27.1 | 1.4 |
| 05.06.2015 | RAS | D | 5.2 | 7.08 | 27.1 | 1.4 |
| 06.06.2015 | Hydro | D | 7.22 | 6.28 | 27.3 | 1.9 |
| 06.06.2015 | RAS | A | 7.2 | 7.18 | 27.6 | 1.12 |
| 06.06.2015 | RAS | A | 7.2 | 7.18 | 27.6 | 1.12 |
| 06.06.2015 | RAS | A | 7.2 | 7.18 | 27.6 | 1.12 |
| 06.06.2015 | RAS | A | 7.2 | 7.18 | 27.6 | 1.12 |
| 06.06.2015 | RAS | C | 7.3 | 7.07 | 27.7 | 1.5 |
| 06.06.2015 | RAS | C | 7.3 | 7.07 | 27.7 | 1.5 |
| 06.06.2015 | RAS | C | 7.3 | 7.07 | 27.7 | 1.5 |
| 06.06.2015 | RAS | C | 7.3 | 7.07 | 27.7 | 1.5 |
| 06.06.2015 | RAS | D | 7.68 | 7.16 | 28.4 | 1.4 |
| 06.06.2015 | RAS | D | 7.68 | 7.16 | 28.4 | 1.4 |
| 06.06.2015 | RAS | D | 7.68 | 7.16 | 28.4 | 1.4 |
| 06.06.2015 | RAS | D | 7.68 | 7.16 | 28.4 | 1.4 |
| 07.06.2015 | Hydro | D | 7.87 | 6.76 | 25.5 | 2.72 |
| 07.06.2015 | RAS | A | 7.7 | 7.74 | 26 |  |
| 07.06.2015 | RAS | A | 7.7 | 7.74 | 26 |  |
| 07.06.2015 | RAS | A | 7.7 | 7.74 | 26 |  |
| 07.06.2015 | RAS | A | 7.7 | 7.74 | 26 |  |
| 07.06.2015 | RAS | C | 7 | 6.71 | 27.8 | 1.5 |
| 07.06.2015 | RAS | C | 7 | 6.71 | 27.8 | 1.5 |
| 07.06.2015 | RAS | C | 7 | 6.71 | 27.8 | 1.5 |
| 07.06.2015 | RAS | C | 7 | 6.71 | 27.8 | 1.5 |
| 07.06.2015 | RAS | D | 4.6 | 6.91 | 28.3 | 1.4 |
| 07.06.2015 | RAS | D | 4.6 | 6.91 | 28.3 | 1.4 |
| 07.06.2015 | RAS | D | 4.6 | 6.91 | 28.3 | 1.4 |
| 07.06.2015 | RAS | D | 4.6 | 6.91 | 28.3 | 1.4 |
| 08.06.2015 | Hydro | D | 8.14 | 7.02 | 24.1 | 3.22 |
| 08.06.2015 | RAS | A | 6.7 | 8.05 | 25 | 0.945 |
| 08.06.2015 | RAS | A | 8.1 | 8.05 | 25 | 0.945 |
| 08.06.2015 | RAS | A | 6.85 | 8.05 | 25 | 0.945 |
| 08.06.2015 | RAS | A | 6 | 8.05 | 25 | 0.945 |
| 08.06.2015 | RAS | C | 6.2 | 6.89 | 26.9 | 1.6 |
| 08.06.2015 | RAS | C | 7.8 | 6.89 | 26.9 | 1.6 |
| 08.06.2015 | RAS | C | 7.9 | 6.89 | 26.9 | 1.6 |
| 08.06.2015 | RAS | C | 8.5 | 6.89 | 26.9 | 1.6 |
| 08.06.2015 | RAS | D | 6 | 6.91 | 27.8 | 1.4 |
| 08.06.2015 | RAS | D | 7.7 | 6.91 | 27.8 | 1.4 |
| 08.06.2015 | RAS | D | 6.5 | 6.91 | 27.8 | 1.4 |
| 08.06.2015 | RAS | D | 6.21 | 6.91 | 27.8 | 1.4 |
| 09.06.2015 | Hydro | D | 8.25 | 5.61 | 23.2 | 3.91 |
| 09.06.2015 | RAS | A | 6.6 | 8.06 | 26.3 | 0.914 |
| 09.06.2015 | RAS | A | 6.1 | 8.06 | 26.3 | 0.914 |
| 09.06.2015 | RAS | A | 6.82 | 8.06 | 26.3 | 0.914 |
| 09.06.2015 | RAS | A | 7.1 | 8.06 | 26.3 | 0.914 |
| 09.06.2015 | RAS | C | 7.3 | 7.59 | 25.1 | 1.4 |
| 09.06.2015 | RAS | C | 7.7 | 7.59 | 25.1 | 1.4 |
| 09.06.2015 | RAS | C |  | 7.59 | 25.1 | 1.4 |
| 09.06.2015 | RAS | C |  | 7.59 | 25.1 | 1.4 |
| 09.06.2015 | RAS | D | 6.8 | 7.61 | 26.2 | 1.3 |
| 09.06.2015 | RAS | D | 6.6 | 7.61 | 26.2 | 1.3 |
| 09.06.2015 | RAS | D | 7.1 | 7.61 | 26.2 | 1.3 |
| 09.06.2015 | RAS | D | 8.1 | 7.61 | 26.2 | 1.3 |
| 10.06.2015 | Hydro | D | 8.24 | 6.16 | 23.5 | 3.14 |
| 10.06.2015 | RAS | A | 6.2 | 7.94 | 26.3 | 0.919 |
| 10.06.2015 | RAS | A | 5.7 | 7.94 | 26.3 | 0.919 |
| 10.06.2015 | RAS | A | 6.72 | 7.94 | 26.3 | 0.919 |
| 10.06.2015 | RAS | A | 7 | 7.94 | 26.3 | 0.919 |
| 10.06.2015 | RAS | C | 6.1 | 7.38 | 26.2 | 1.4 |
| 10.06.2015 | RAS | C | 7 | 7.38 | 26.2 | 1.4 |
| 10.06.2015 | RAS | C | 8.5 | 7.38 | 26.2 | 1.4 |
| 10.06.2015 | RAS | C |  | 7.38 | 26.2 | 1.4 |
| 10.06.2015 | RAS | D | 6 | 7.43 | 26.4 | 1.3 |
| 10.06.2015 | RAS | D | 6.1 | 7.43 | 26.4 | 1.3 |
| 10.06.2015 | RAS | D | 6.4 | 7.43 | 26.4 | 1.3 |
| 10.06.2015 | RAS | D | 8.21 | 7.43 | 26.4 | 1.3 |
| 11.06.2015 | Hydro | D | 8.13 | 7.03 | 23.4 | 3.6 |
| 11.06.2015 | RAS | A | 6.6 | 7.71 | 26 | 0.927 |
| 11.06.2015 | RAS | A | 5.4 | 7.71 | 26 | 0.927 |
| 11.06.2015 | RAS | A | 6.6 | 7.71 | 26 | 0.927 |
| 11.06.2015 | RAS | A | 7.6 | 7.71 | 26 | 0.927 |
| 11.06.2015 | RAS | C | 5.9 | 7.1 | 26.2 | 1.4 |
| 11.06.2015 | RAS | C | 6.1 | 7.1 | 26.2 | 1.4 |
| 11.06.2015 | RAS | C | 5.7 | 7.1 | 26.2 | 1.4 |
| 11.06.2015 | RAS | C | 7.6 | 7.1 | 26.2 | 1.4 |
| 11.06.2015 | RAS | D | 6 | 7.16 | 26 | 1.4 |
| 11.06.2015 | RAS | D | 5.6 | 7.16 | 26 | 1.4 |
| 11.06.2015 | RAS | D | 5.3 | 7.16 | 26 | 1.4 |
| 11.06.2015 | RAS | D | 5.1 | 7.16 | 26 | 1.4 |
| 12.06.2015 | Hydro | D | 8.19 | 5.72 | 23.5 | 4.58 |
| 12.06.2015 | RAS | A | 5.8 | 7.54 | 26.9 | 0.94 |
| 12.06.2015 | RAS | A | 6.08 | 7.54 | 26.9 | 0.94 |
| 12.06.2015 | RAS | A | 6.2 | 7.54 | 26.9 | 0.94 |
| 12.06.2015 | RAS | A | 7.3 | 7.54 | 26.9 | 0.94 |
| 12.06.2015 | RAS | C | 5.2 | 6.67 | 26.5 | 1.4 |
| 12.06.2015 | RAS | C | 5.8 | 6.67 | 26.5 | 1.4 |
| 12.06.2015 | RAS | C | 4.9 | 6.67 | 26.5 | 1.4 |
| 12.06.2015 | RAS | C | 7.4 | 6.67 | 26.5 | 1.4 |
| 12.06.2015 | RAS | D | 5.2 | 6.77 | 27 | 1.4 |
| 12.06.2015 | RAS | D | 5.48 | 6.77 | 27 | 1.4 |
| 12.06.2015 | RAS | D | 5.94 | 6.77 | 27 | 1.4 |
| 12.06.2015 | RAS | D | 4.9 | 6.77 | 27 | 1.4 |
| 13.06.2015 | Hydro | D | 7.26 | 5.7 | 25.7 | 2.57 |
| 13.06.2015 | RAS | A | 7 | 7.54 | 27.4 | 0.9 |
| 13.06.2015 | RAS | A | 7 | 7.54 | 27.4 | 0.9 |
| 13.06.2015 | RAS | A | 7 | 7.54 | 27.4 | 0.9 |
| 13.06.2015 | RAS | A | 7 | 7.54 | 27.4 | 0.9 |
| 13.06.2015 | RAS | C | 7.1 | 7.24 | 27 | 1.5 |
| 13.06.2015 | RAS | C | 7.1 | 7.24 | 27 | 1.5 |
| 13.06.2015 | RAS | C | 7.1 | 7.24 | 27 | 1.5 |
| 13.06.2015 | RAS | C | 7.1 | 7.24 | 27 | 1.5 |
| 13.06.2015 | RAS | D |  | 7.23 | 27.8 | 1.4 |
| 13.06.2015 | RAS | D |  | 7.23 | 27.8 | 1.4 |
| 13.06.2015 | RAS | D |  | 7.23 | 27.8 | 1.4 |
| 13.06.2015 | RAS | D |  | 7.23 | 27.8 | 1.4 |
| 14.06.2015 | Hydro | D | 7.76 | 5.95 | 25.6 | 2.8 |
| 14.06.2015 | RAS | A | 6.8 | 7.37 | 28.1 | 0.953 |
| 14.06.2015 | RAS | A | 6.8 | 7.37 | 28.1 | 0.953 |
| 14.06.2015 | RAS | A | 6.8 | 7.37 | 28.1 | 0.953 |
| 14.06.2015 | RAS | A | 6.8 | 7.37 | 28.1 | 0.953 |
| 14.06.2015 | RAS | C | 6.9 | 6.86 | 27.7 | 1.5 |
| 14.06.2015 | RAS | C | 6.9 | 6.86 | 27.7 | 1.5 |
| 14.06.2015 | RAS | C | 6.9 | 6.86 | 27.7 | 1.5 |
| 14.06.2015 | RAS | C | 6.9 | 6.86 | 27.7 | 1.5 |
| 14.06.2015 | RAS | D | 8.1 | 6.87 | 28.5 | 1.4 |
| 14.06.2015 | RAS | D | 8.1 | 6.87 | 28.5 | 1.4 |
| 14.06.2015 | RAS | D | 8.1 | 6.87 | 28.5 | 1.4 |
| 14.06.2015 | RAS | D | 8.1 | 6.87 | 28.5 | 1.4 |
| 15.06.2015 | Hydro | D | 8.2 | 5.8 | 23.8 | 3.17 |
| 15.06.2015 | RAS | A | 4.1 | 7.53 | 27.6 | 0.984 |
| 15.06.2015 | RAS | A | 6.01 | 7.53 | 27.6 | 0.984 |
| 15.06.2015 | RAS | A | 6.6 | 7.53 | 27.6 | 0.984 |
| 15.06.2015 | RAS | A | 7.4 | 7.53 | 27.6 | 0.984 |
| 15.06.2015 | RAS | C | 4.4 | 7.07 | 27.1 | 1.5 |
| 15.06.2015 | RAS | C | 5.7 | 7.07 | 27.1 | 1.5 |
| 15.06.2015 | RAS | C | 5.3 | 7.07 | 27.1 | 1.5 |
| 15.06.2015 | RAS | C | 7.5 | 7.07 | 27.1 | 1.5 |
| 15.06.2015 | RAS | D | 4.7 | 7.05 | 28.1 | 1.5 |
| 15.06.2015 | RAS | D | 5.66 | 7.05 | 28.1 | 1.5 |
| 15.06.2015 | RAS | D | 5.77 | 7.05 | 28.1 | 1.5 |
| 15.06.2015 | RAS | D | 4.8 | 7.05 | 28.1 | 1.5 |
| 16.06.2015 | Hydro | D | 8.26 | 6.36 | 23.5 | 3.86 |
| 16.06.2015 | RAS | A | 6.1 | 7.28 | 26.3 | 0.981 |
| 16.06.2015 | RAS | A | 6.52 | 7.28 | 26.3 | 0.981 |
| 16.06.2015 | RAS | A | 7.1 | 7.28 | 26.3 | 0.981 |
| 16.06.2015 | RAS | A | 7.7 | 7.28 | 26.3 | 0.981 |
| 16.06.2015 | RAS | C | 5.2 | 6.86 | 26.2 | 1.4 |
| 16.06.2015 | RAS | C | 6.4 | 6.86 | 26.2 | 1.4 |
| 16.06.2015 | RAS | C | 5.1 | 6.86 | 26.2 | 1.4 |
| 16.06.2015 | RAS | C | 7.6 | 6.86 | 26.2 | 1.4 |
| 16.06.2015 | RAS | D | 5.3 | 6.91 | 26.4 | 1.4 |
| 16.06.2015 | RAS | D | 6.04 | 6.91 | 26.4 | 1.4 |
| 16.06.2015 | RAS | D | 6.215 | 6.91 | 26.4 | 1.4 |
| 16.06.2015 | RAS | D | 5 | 6.91 | 26.4 | 1.4 |
| 17.06.2015 | Hydro | D | 8.34 | 6.82 | 23.1 | 4.57 |
| 17.06.2015 | RAS | A | 6.1 | 7.48 | 26.2 | 0.961 |
| 17.06.2015 | RAS | A | 6.36 | 7.48 | 26.2 | 0.961 |
| 17.06.2015 | RAS | A | 7.3 | 7.48 | 26.2 | 0.961 |
| 17.06.2015 | RAS | A | 7.7 | 7.48 | 26.2 | 0.961 |
| 17.06.2015 | RAS | C | 5.1 | 7.08 | 26.2 | 1.4 |
| 17.06.2015 | RAS | C | 5.7 | 7.08 | 26.2 | 1.4 |
| 17.06.2015 | RAS | C | 5.2 | 7.08 | 26.2 | 1.4 |
| 17.06.2015 | RAS | C | 7.7 | 7.08 | 26.2 | 1.4 |
| 17.06.2015 | RAS | D | 5.1 | 7.14 | 25.9 | 1.5 |
| 17.06.2015 | RAS | D | 5.1 | 7.14 | 25.9 | 1.5 |
| 17.06.2015 | RAS | D | 5.92 | 7.14 | 25.9 | 1.5 |
| 17.06.2015 | RAS | D | 5 | 7.14 | 25.9 | 1.5 |
| 18.06.2015 | Hydro | D | 8.13 | 7.12 | 23.8 | 5.63 |
| 18.06.2015 | RAS | A | 5.8 | 7.15 | 26.2 | 1.197 |
| 18.06.2015 | RAS | A | 6.47 | 7.15 | 26.2 | 1.197 |
| 18.06.2015 | RAS | A | 7.5 | 7.15 | 26.2 | 1.197 |
| 18.06.2015 | RAS | A | 7.6 | 7.15 | 26.2 | 1.197 |
| 18.06.2015 | RAS | C | 4.7 | 6.95 | 26 | 1.5 |
| 18.06.2015 | RAS | C | 5.7 | 6.95 | 26 | 1.5 |
| 18.06.2015 | RAS | C | 5.3 | 6.95 | 26 | 1.5 |
| 18.06.2015 | RAS | C | 7.6 | 6.95 | 26 | 1.5 |
| 18.06.2015 | RAS | D | 4.6 | 7.04 | 26.5 | 1.5 |
| 18.06.2015 | RAS | D | 5.72 | 7.04 | 26.5 | 1.5 |
| 18.06.2015 | RAS | D | 6.26 | 7.04 | 26.5 | 1.5 |
| 18.06.2015 | RAS | D | 4.9 | 7.04 | 26.5 | 1.5 |
| 19.06.2015 | Hydro | D | 7.83 | 5.12 | 25.4 | 2.2 |
| 19.06.2015 | RAS | A | 4.2 | 7.11 | 26.2 | 1.003 |
| 19.06.2015 | RAS | A | 6.18 | 7.11 | 26.2 | 1.003 |
| 19.06.2015 | RAS | A | 7.5 | 7.11 | 26.2 | 1.003 |
| 19.06.2015 | RAS | A | 7.6 | 7.11 | 26.2 | 1.003 |
| 19.06.2015 | RAS | C | 4.8 | 6.86 | 26.1 | 1.5 |
| 19.06.2015 | RAS | C | 5.6 | 6.86 | 26.1 | 1.5 |
| 19.06.2015 | RAS | C | 5.39 | 6.86 | 26.1 | 1.5 |
| 19.06.2015 | RAS | C | 7.5 | 6.86 | 26.1 | 1.5 |
| 19.06.2015 | RAS | D | 4.9 | 7 | 26.7 | 1.6 |
| 19.06.2015 | RAS | D | 5.46 | 7 | 26.7 | 1.6 |
| 19.06.2015 | RAS | D | 5.98 | 7 | 26.7 | 1.6 |
| 19.06.2015 | RAS | D | 4.8 | 7 | 26.7 | 1.6 |
| 20.06.2015 | Hydro | D | 8.18 | 5.36 | 23.7 | 2.28 |
| 20.06.2015 | RAS | A | 7.1 | 7.4 | 26 |  |
| 20.06.2015 | RAS | A | 7.1 | 7.4 | 26 |  |
| 20.06.2015 | RAS | A | 7.1 | 7.4 | 26 |  |
| 20.06.2015 | RAS | A | 7.1 | 7.4 | 26 |  |
| 20.06.2015 | RAS | C | 7.2 | 7.1 | 26.2 | 1.6 |
| 20.06.2015 | RAS | C | 7.2 | 7.1 | 26.2 | 1.6 |
| 20.06.2015 | RAS | C | 7.2 | 7.1 | 26.2 | 1.6 |
| 20.06.2015 | RAS | C | 7.2 | 7.1 | 26.2 | 1.6 |
| 20.06.2015 | RAS | D | 5.5 | 7.2 | 26.5 | 1.6 |
| 20.06.2015 | RAS | D | 5.5 | 7.2 | 26.5 | 1.6 |
| 20.06.2015 | RAS | D | 5.5 | 7.2 | 26.5 | 1.6 |
| 20.06.2015 | RAS | D | 5.5 | 7.2 | 26.5 | 1.6 |
| 21.06.2015 | Hydro | D | 8.26 | 5.01 | 23.4 | 2.39 |
| 21.06.2015 | RAS | A | 7.1 | 7.27 | 26.1 | 1.1 |
| 21.06.2015 | RAS | A | 7.1 | 7.27 | 26.1 | 1.1 |
| 21.06.2015 | RAS | A | 7.1 | 7.27 | 26.1 | 1.1 |
| 21.06.2015 | RAS | A | 7.1 | 7.27 | 26.1 | 1.1 |
| 21.06.2015 | RAS | C | 7.2 | 6.86 | 26.2 | 1.6 |
| 21.06.2015 | RAS | C | 7.2 | 6.86 | 26.2 | 1.6 |
| 21.06.2015 | RAS | C | 7.2 | 6.86 | 26.2 | 1.6 |
| 21.06.2015 | RAS | C | 7.2 | 6.86 | 26.2 | 1.6 |
| 21.06.2015 | RAS | D | 5.8 | 7.07 | 26.7 | 1.6 |
| 21.06.2015 | RAS | D | 5.8 | 7.07 | 26.7 | 1.6 |
| 21.06.2015 | RAS | D | 5.8 | 7.07 | 26.7 | 1.6 |
| 21.06.2015 | RAS | D | 5.8 | 7.07 | 26.7 | 1.6 |
| 22.06.2015 | Hydro | D | 8.23 | 5.49 | 23.4 | 2.55 |
| 22.06.2015 | RAS | A | 4.3 | 7.21 | 26.1 | 1.074 |
| 22.06.2015 | RAS | A | 6.21 | 7.21 | 26.1 | 1.074 |
| 22.06.2015 | RAS | A | 7.4 | 7.21 | 26.1 | 1.074 |
| 22.06.2015 | RAS | A | 7.6 | 7.21 | 26.1 | 1.074 |
| 22.06.2015 | RAS | C | 4.3 | 7.16 | 25.9 | 1.7 |
| 22.06.2015 | RAS | C | 5.5 | 7.16 | 25.9 | 1.7 |
| 22.06.2015 | RAS | C | 5.52 | 7.16 | 25.9 | 1.7 |
| 22.06.2015 | RAS | C | 7.6 | 7.16 | 25.9 | 1.7 |
| 22.06.2015 | RAS | D |  | 7.2 | 26.7 | 1.7 |
| 22.06.2015 | RAS | D | 5.06 | 7.2 | 26.7 | 1.7 |
| 22.06.2015 | RAS | D | 6.06 | 7.2 | 26.7 | 1.7 |
| 22.06.2015 | RAS | D | 4.7 | 7.2 | 26.7 | 1.7 |
| 23.06.2015 | Hydro | D | 8.34 | 6.09 | 22.5 | 2.74 |
| 23.06.2015 | RAS | A | 5.9 | 6.88 | 26 | 1.086 |
| 23.06.2015 | RAS | A | 6.45 | 6.88 | 26 | 1.086 |
| 23.06.2015 | RAS | A | 8 | 6.88 | 26 | 1.086 |
| 23.06.2015 | RAS | A | 8.1 | 6.88 | 26 | 1.086 |
| 23.06.2015 | RAS | C | 4.9 | 7.08 | 26.2 | 1.6 |
| 23.06.2015 | RAS | C | 5.8 | 7.08 | 26.2 | 1.6 |
| 23.06.2015 | RAS | C | 5.1 | 7.08 | 26.2 | 1.6 |
| 23.06.2015 | RAS | C | 7.4 | 7.08 | 26.2 | 1.6 |
| 23.06.2015 | RAS | D | 4.9 | 7.17 | 26 | 1.6 |
| 23.06.2015 | RAS | D | 5.48 | 7.17 | 26 | 1.6 |
| 23.06.2015 | RAS | D | 6.36 | 7.17 | 26 | 1.6 |
| 23.06.2015 | RAS | D | 4.6 | 7.17 | 26 | 1.6 |
| 24.06.2015 | Hydro | D | 8.44 | 6.18 | 22.7 | 2.89 |
| 24.06.2015 | RAS | A | 5.1 | 7.19 | 26.2 | 1.116 |
| 24.06.2015 | RAS | A | 6.37 | 7.19 | 26.2 | 1.116 |
| 24.06.2015 | RAS | A | 7.7 | 7.19 | 26.2 | 1.116 |
| 24.06.2015 | RAS | A | 8 | 7.19 | 26.2 | 1.116 |
| 24.06.2015 | RAS | C | 4.9 | 7.09 | 26 | 1.7 |
| 24.06.2015 | RAS | C | 5.6 | 7.09 | 26 | 1.7 |
| 24.06.2015 | RAS | C | 5.78 | 7.09 | 26 | 1.7 |
| 24.06.2015 | RAS | C | 7.6 | 7.09 | 26 | 1.7 |
| 24.06.2015 | RAS | D | 4.3 | 7.08 | 25.9 | 1.6 |
| 24.06.2015 | RAS | D | 5.44 | 7.08 | 25.9 | 1.6 |
| 24.06.2015 | RAS | D | 6.44 | 7.08 | 25.9 | 1.6 |
| 24.06.2015 | RAS | D | 4.7 | 7.08 | 25.9 | 1.6 |
| 25.06.2015 | Hydro | D | 8.41 | 5.71 | 23.3 | 3.15 |
| 25.06.2015 | RAS | A | 4.9 | 7.42 | 25.9 | 1.129 |
| 25.06.2015 | RAS | A | 6.09 | 7.42 | 25.9 | 1.129 |
| 25.06.2015 | RAS | A | 8.1 | 7.42 | 25.9 | 1.129 |
| 25.06.2015 | RAS | A | 7.8 | 7.42 | 25.9 | 1.129 |
| 25.06.2015 | RAS | C | 4.8 | 7.21 | 26.2 | 1.7 |
| 25.06.2015 | RAS | C | 5.4 | 7.21 | 26.2 | 1.7 |
| 25.06.2015 | RAS | C | 5.15 | 7.21 | 26.2 | 1.7 |
| 25.06.2015 | RAS | C | 7.6 | 7.21 | 26.2 | 1.7 |
| 25.06.2015 | RAS | D |  | 7.19 | 26.3 | 1.7 |
| 25.06.2015 | RAS | D | 5.14 | 7.19 | 26.3 | 1.7 |
| 25.06.2015 | RAS | D | 6.04 | 7.19 | 26.3 | 1.7 |
| 25.06.2015 | RAS | D | 4.7 | 7.19 | 26.3 | 1.7 |
| 26.06.2015 | Hydro | D | 8.06 | 5.4 | 23.8 | 2.68 |
| 26.06.2015 | RAS | A | 5.9 | 7.45 | 26.3 | 1.135 |
| 26.06.2015 | RAS | A | 5.69 | 7.45 | 26.3 | 1.135 |
| 26.06.2015 | RAS | A | 6.5 | 7.45 | 26.3 | 1.135 |
| 26.06.2015 | RAS | A | 7.8 | 7.45 | 26.3 | 1.135 |
| 26.06.2015 | RAS | C | 5.9 | 7.01 | 26.4 | 1.7 |
| 26.06.2015 | RAS | C | 6.1 | 7.01 | 26.4 | 1.7 |
| 26.06.2015 | RAS | C | 5.02 | 7.01 | 26.4 | 1.7 |
| 26.06.2015 | RAS | C | 7.7 | 7.01 | 26.4 | 1.7 |
| 26.06.2015 | RAS | D | 9.1 | 7.1 | 26.9 | 1.7 |
| 26.06.2015 | RAS | D | 4.6 | 7.1 | 26.9 | 1.7 |
| 26.06.2015 | RAS | D | 5.9 | 7.1 | 26.9 | 1.7 |
| 26.06.2015 | RAS | D | 7.5 | 7.1 | 26.9 | 1.7 |
| 27.06.2015 | Hydro | D | 7.99 | 5.48 | 24.5 | 0.962 |
| 27.06.2015 | RAS | A | 7.3 | 7.3 | 26.7 | 1.036 |
| 27.06.2015 | RAS | A | 7.3 | 7.3 | 26.7 | 1.036 |
| 27.06.2015 | RAS | A | 7.3 | 7.3 | 26.7 | 1.036 |
| 27.06.2015 | RAS | A | 7.3 | 7.3 | 26.7 | 1.036 |
| 27.06.2015 | RAS | C | 7.2 | 7.11 | 26.5 | 1.033 |
| 27.06.2015 | RAS | C | 7.2 | 7.11 | 26.5 | 1.033 |
| 27.06.2015 | RAS | C | 7.2 | 7.11 | 26.5 | 1.033 |
| 27.06.2015 | RAS | C | 7.2 | 7.11 | 26.5 | 1.033 |
| 27.06.2015 | RAS | D | 6.8 | 7.17 | 27.3 | 1.046 |
| 27.06.2015 | RAS | D | 6.8 | 7.17 | 27.3 | 1.046 |
| 27.06.2015 | RAS | D | 6.8 | 7.17 | 27.3 | 1.046 |
| 27.06.2015 | RAS | D | 6.8 | 7.17 | 27.3 | 1.046 |
| 28.06.2015 | Hydro | D | 8.17 | 5.57 | 24.3 | 2.89 |
| 28.06.2015 | RAS | A | 7.3 | 7.19 | 26.8 | 1.249 |
| 28.06.2015 | RAS | A | 7.3 | 7.19 | 26.8 | 1.249 |
| 28.06.2015 | RAS | A | 7.3 | 7.19 | 26.8 | 1.249 |
| 28.06.2015 | RAS | A | 7.3 | 7.19 | 26.8 | 1.249 |
| 28.06.2015 | RAS | C | 7.2 | 6.79 | 26.5 | 1.8 |
| 28.06.2015 | RAS | C | 7.2 | 6.79 | 26.5 | 1.8 |
| 28.06.2015 | RAS | C | 7.2 | 6.79 | 26.5 | 1.8 |
| 28.06.2015 | RAS | C | 7.2 | 6.79 | 26.5 | 1.8 |
| 28.06.2015 | RAS | D | 6.9 | 6.97 | 27.5 | 1.7 |
| 28.06.2015 | RAS | D | 6.9 | 6.97 | 27.5 | 1.7 |
| 28.06.2015 | RAS | D | 6.9 | 6.97 | 27.5 | 1.7 |
| 28.06.2015 | RAS | D | 6.9 | 6.97 | 27.5 | 1.7 |
| 29.06.2015 | Hydro | D | 8.16 | 5.87 | 24.8 | 3.2 |
| 29.06.2015 | RAS | A | 5.2 | 7.35 | 26.8 | 1.141 |
| 29.06.2015 | RAS | A | 6.02 | 7.35 | 26.8 | 1.141 |
| 29.06.2015 | RAS | A | 5 | 7.35 | 26.8 | 1.141 |
| 29.06.2015 | RAS | A | 7.6 | 7.35 | 26.8 | 1.141 |
| 29.06.2015 | RAS | C | 6.2 | 6.9 | 26.6 | 1.8 |
| 29.06.2015 | RAS | C | 6.83 | 6.9 | 26.6 | 1.8 |
| 29.06.2015 | RAS | C | 5.3 | 6.9 | 26.6 | 1.8 |
| 29.06.2015 | RAS | C | 7.6 | 6.9 | 26.6 | 1.8 |
| 29.06.2015 | RAS | D | 6.1 | 7.06 | 27.5 | 1.8 |
| 29.06.2015 | RAS | D | 5.74 | 7.06 | 27.5 | 1.8 |
| 29.06.2015 | RAS | D | 6.3 | 7.06 | 27.5 | 1.8 |
| 29.06.2015 | RAS | D | 7.5 | 7.06 | 27.5 | 1.8 |
| 30.06.2015 | Hydro | D | 8.19 | 5.78 | 24.5 | 3.96 |
| 30.06.2015 | RAS | A | 5.7 | 7.27 | 27.4 | 1.162 |
| 30.06.2015 | RAS | A | 6.13 | 7.27 | 27.4 | 1.162 |
| 30.06.2015 | RAS | A | 6.4 | 7.27 | 27.4 | 1.162 |
| 30.06.2015 | RAS | A | 7.5 | 7.27 | 27.4 | 1.162 |
| 30.06.2015 | RAS | C | 5.3 | 7.11 | 26.6 | 1.8 |
| 30.06.2015 | RAS | C | 6.62 | 7.11 | 26.6 | 1.8 |
| 30.06.2015 | RAS | C | 5.73 | 7.11 | 26.6 | 1.8 |
| 30.06.2015 | RAS | C | 7.6 | 7.11 | 26.6 | 1.8 |
| 30.06.2015 | RAS | D | 5.6 | 7.14 | 27.4 | 1.7 |
| 30.06.2015 | RAS | D | 5.3 | 7.14 | 27.4 | 1.7 |
| 30.06.2015 | RAS | D | 6.03 | 7.14 | 27.4 | 1.7 |
| 30.06.2015 | RAS | D | 7.5 | 7.14 | 27.4 | 1.7 |
| 01.07.2015 | Hydro | D | 8.16 | 6.01 | 24.4 | 5.61 |
| 01.07.2015 | RAS | A | 4.3 | 7.5 | 27.6 | 1.189 |
| 01.07.2015 | RAS | A | 5.39 | 7.5 | 27.6 | 1.189 |
| 01.07.2015 | RAS | A | 6.2 | 7.5 | 27.6 | 1.189 |
| 01.07.2015 | RAS | A | 7.4 | 7.5 | 27.6 | 1.189 |
| 01.07.2015 | RAS | C | 5.1 | 7.11 | 27.2 | 1.8 |
| 01.07.2015 | RAS | C | 6.26 | 7.11 | 27.2 | 1.8 |
| 01.07.2015 | RAS | C | 5.19 | 7.11 | 27.2 | 1.8 |
| 01.07.2015 | RAS | C | 7.4 | 7.11 | 27.2 | 1.8 |
| 01.07.2015 | RAS | D | 5.3 | 7.16 | 28 | 1.8 |
| 01.07.2015 | RAS | D | 4.97 | 7.16 | 28 | 1.8 |
| 01.07.2015 | RAS | D | 6.13 | 7.16 | 28 | 1.8 |
| 01.07.2015 | RAS | D | 7.4 | 7.16 | 28 | 1.8 |
| 02.07.2015 | Hydro | D | 7.63 | 6.84 | 25.9 | 2.59 |
| 02.07.2015 | RAS | A |  | 7.31 | 27.8 | 1.167 |
| 02.07.2015 | RAS | A | 5.35 | 7.31 | 27.8 | 1.167 |
| 02.07.2015 | RAS | A | 6.1 | 7.31 | 27.8 | 1.167 |
| 02.07.2015 | RAS | A | 7.3 | 7.31 | 27.8 | 1.167 |
| 02.07.2015 | RAS | C | 5 | 6.95 | 27.8 | 1.8 |
| 02.07.2015 | RAS | C | 6.27 | 6.95 | 27.8 | 1.8 |
| 02.07.2015 | RAS | C | 4.6 | 6.95 | 27.8 | 1.8 |
| 02.07.2015 | RAS | C | 7.2 | 6.95 | 27.8 | 1.8 |
| 02.07.2015 | RAS | D | 4.6 | 7.03 | 28.4 | 1.8 |
| 02.07.2015 | RAS | D | 4.55 | 7.03 | 28.4 | 1.8 |
| 02.07.2015 | RAS | D | 5.61 | 7.03 | 28.4 | 1.8 |
| 02.07.2015 | RAS | D | 7.3 | 7.03 | 28.4 | 1.8 |
| 03.07.2015 | Hydro | D | 7.63 | 6.77 | 26.6 | 2.87 |
| 03.07.2015 | RAS | A |  | 7.3 | 28.6 | 1.187 |
| 03.07.2015 | RAS | A | 4.99 | 7.3 | 28.6 | 1.187 |
| 03.07.2015 | RAS | A | 5.95 | 7.3 | 28.6 | 1.187 |
| 03.07.2015 | RAS | A | 7.2 | 7.3 | 28.6 | 1.187 |
| 03.07.2015 | RAS | C | 5.1 | 7.11 | 28.7 | 1.9 |
| 03.07.2015 | RAS | C | 6.03 | 7.11 | 28.7 | 1.9 |
| 03.07.2015 | RAS | C | 4.87 | 7.11 | 28.7 | 1.9 |
| 03.07.2015 | RAS | C | 7.2 | 7.11 | 28.7 | 1.9 |
| 03.07.2015 | RAS | D | 5.2 | 7.16 | 29.3 | 1.8 |
| 03.07.2015 | RAS | D | 4.51 | 7.16 | 29.3 | 1.8 |
| 03.07.2015 | RAS | D | 5.38 | 7.16 | 29.3 | 1.8 |
| 03.07.2015 | RAS | D | 7.2 | 7.16 | 29.3 | 1.8 |
| 04.07.2015 | Hydro | D | 7.36 | 7.17 | 28.5 | 3.5 |
| 04.07.2015 | RAS | A | 7.26 | 7.28 | 29.4 |  |
| 04.07.2015 | RAS | A | 7.26 | 7.28 | 29.4 |  |
| 04.07.2015 | RAS | A | 7.26 | 7.28 | 29.4 |  |
| 04.07.2015 | RAS | A | 7.26 | 7.28 | 29.4 |  |
| 04.07.2015 | RAS | C | 6.4 | 7.31 | 29.6 | 1.9 |
| 04.07.2015 | RAS | C | 6.4 | 7.31 | 29.6 | 1.9 |
| 04.07.2015 | RAS | C | 6.4 | 7.31 | 29.6 | 1.9 |
| 04.07.2015 | RAS | C | 6.4 | 7.31 | 29.6 | 1.9 |
| 04.07.2015 | RAS | D | 5 | 7.2 | 30.3 | 1.9 |
| 04.07.2015 | RAS | D | 5 | 7.2 | 30.3 | 1.9 |
| 04.07.2015 | RAS | D | 5 | 7.2 | 30.3 | 1.9 |
| 04.07.2015 | RAS | D | 5 | 7.2 | 30.3 | 1.9 |
| 05.07.2015 | Hydro | D | 7.32 | 7.18 |  | 3.9 |
| 05.07.2015 | RAS | A | 6.8 | 7.33 | 30.1 | 1.349 |
| 05.07.2015 | RAS | A | 6.8 | 7.33 | 30.1 | 1.349 |
| 05.07.2015 | RAS | A | 6.8 | 7.33 | 30.1 | 1.349 |
| 05.07.2015 | RAS | A | 6.8 | 7.33 | 30.1 | 1.349 |
| 05.07.2015 | RAS | C | 6.5 | 7.37 | 29.7 | 1.977 |
| 05.07.2015 | RAS | C | 6.5 | 7.37 | 29.7 | 1.977 |
| 05.07.2015 | RAS | C | 6.5 | 7.37 | 29.7 | 1.977 |
| 05.07.2015 | RAS | C | 6.5 | 7.37 | 29.7 | 1.977 |
| 05.07.2015 | RAS | D | 4.8 | 7.17 | 31 | 1.955 |
| 05.07.2015 | RAS | D | 4.8 | 7.17 | 31 | 1.955 |
| 05.07.2015 | RAS | D | 4.8 | 7.17 | 31 | 1.955 |
| 05.07.2015 | RAS | D | 4.8 | 7.17 | 31 | 1.955 |
| 06.07.2015 | Hydro | D |  | 5.99 | 26.3 | 5.74 |
| 06.07.2015 | RAS | A | 4.8 | 7.32 | 30.1 | 1.238 |
| 06.07.2015 | RAS | A | 5.98 | 7.32 | 30.1 | 1.238 |
| 06.07.2015 | RAS | A | 6.32 | 7.32 | 30.1 | 1.238 |
| 06.07.2015 | RAS | A | 6.9 | 7.32 | 30.1 | 1.238 |
| 06.07.2015 | RAS | C | 4.2 | 7.39 | 29.4 | 1.9 |
| 06.07.2015 | RAS | C |  | 7.39 | 29.4 | 1.9 |
| 06.07.2015 | RAS | C |  | 7.39 | 29.4 | 1.9 |
| 06.07.2015 | RAS | C | 7.1 | 7.39 | 29.4 | 1.9 |
| 06.07.2015 | RAS | D |  | 7.48 | 30.9 | 2 |
| 06.07.2015 | RAS | D | 4.15 | 7.48 | 30.9 | 2 |
| 06.07.2015 | RAS | D | 4.78 | 7.48 | 30.9 | 2 |
| 06.07.2015 | RAS | D | 4.6 | 7.48 | 30.9 | 2 |
| 07.07.2015 | Hydro | D | 8.06 | 6.64 | 25.2 | 3.54 |
| 07.07.2015 | RAS | A | 4.8 | 7.15 | 28.2 | 1.222 |
| 07.07.2015 | RAS | A |  | 7.15 | 28.2 | 1.222 |
| 07.07.2015 | RAS | A |  | 7.15 | 28.2 | 1.222 |
| 07.07.2015 | RAS | A | 7.2 | 7.15 | 28.2 | 1.222 |
| 07.07.2015 | RAS | C | 4.7 | 7.23 | 27.3 | 1.8 |
| 07.07.2015 | RAS | C |  | 7.23 | 27.3 | 1.8 |
| 07.07.2015 | RAS | C | 4 | 7.23 | 27.3 | 1.8 |
| 07.07.2015 | RAS | C | 7.4 | 7.23 | 27.3 | 1.8 |
| 07.07.2015 | RAS | D | 4 | 7.4 | 28.5 | 1.9 |
| 07.07.2015 | RAS | D |  | 7.4 | 28.5 | 1.9 |
| 07.07.2015 | RAS | D |  | 7.4 | 28.5 | 1.9 |
| 07.07.2015 | RAS | D | 6.5 | 7.4 | 28.5 | 1.9 |
| 08.07.2015 | Hydro | D | 7.2 | 6.72 | 25.8 | 4.23 |
| 08.07.2015 | RAS | A |  | 7.39 | 28.2 |  |
| 08.07.2015 | RAS | A | 4.6 | 7.39 | 28.2 |  |
| 08.07.2015 | RAS | A |  | 7.39 | 28.2 |  |
| 08.07.2015 | RAS | A | 7.2 | 7.39 | 28.2 |  |
| 08.07.2015 | RAS | C | 4.2 | 7.31 | 28 | 1.9 |
| 08.07.2015 | RAS | C |  | 7.31 | 28 | 1.9 |
| 08.07.2015 | RAS | C |  | 7.31 | 28 | 1.9 |
| 08.07.2015 | RAS | C | 7.2 | 7.31 | 28 | 1.9 |
| 08.07.2015 | RAS | D | 3.9 | 7.18 | 29 | 1.9 |
| 08.07.2015 | RAS | D |  | 7.18 | 29 | 1.9 |
| 08.07.2015 | RAS | D |  | 7.18 | 29 | 1.9 |
| 08.07.2015 | RAS | D | 6.5 | 7.18 | 29 | 1.9 |
| 09.07.2015 | Hydro | D |  |  |  |  |
| 09.07.2015 | RAS | A | 4.6 | 7.06 | 27.6 |  |
| 09.07.2015 | RAS | A | 4.5 | 7.06 | 27.6 |  |
| 09.07.2015 | RAS | A |  | 7.06 | 27.6 |  |
| 09.07.2015 | RAS | A | 7.4 | 7.06 | 27.6 |  |
| 09.07.2015 | RAS | C | 5 | 6.92 | 27.4 | 1.9 |
| 09.07.2015 | RAS | C |  | 6.92 | 27.4 | 1.9 |
| 09.07.2015 | RAS | C | 4.4 | 6.92 | 27.4 | 1.9 |
| 09.07.2015 | RAS | C | 7.4 | 6.92 | 27.4 | 1.9 |
| 09.07.2015 | RAS | D | 5 | 7.2 | 28.3 | 1.9 |
| 09.07.2015 | RAS | D | 3.9 | 7.2 | 28.3 | 1.9 |
| 09.07.2015 | RAS | D |  | 7.2 | 28.3 | 1.9 |
| 09.07.2015 | RAS | D | 6.7 | 7.2 | 28.3 | 1.9 |
| 10.07.2015 | Hydro | D | 8.38 | 7.5 | 24.3 | 4.05 |
| 10.07.2015 | RAS | A | 4.6 | 7.63 | 25.9 | 1.137 |
| 10.07.2015 | RAS | A | 5 | 7.63 | 25.9 | 1.137 |
| 10.07.2015 | RAS | A | 7.14 | 7.63 | 25.9 | 1.137 |
| 10.07.2015 | RAS | A | 6 | 7.63 | 25.9 | 1.137 |
| 10.07.2015 | RAS | C | 4.5 | 7.52 | 26.2 | 1.7 |
| 10.07.2015 | RAS | C | 6.61 | 7.52 | 26.2 | 1.7 |
| 10.07.2015 | RAS | C | 5.97 | 7.52 | 26.2 | 1.7 |
| 10.07.2015 | RAS | C | 5.4 | 7.52 | 26.2 | 1.7 |
| 10.07.2015 | RAS | D | 4.7 | 7.65 | 26.1 | 1.7 |
| 10.07.2015 | RAS | D | 5.82 | 7.65 | 26.1 | 1.7 |
| 10.07.2015 | RAS | D | 5.97 | 7.65 | 26.1 | 1.7 |
| 10.07.2015 | RAS | D | 6 | 7.65 | 26.1 | 1.7 |
| 11.07.2015 | Hydro | D | 7.97 | 7.84 | 25.4 | 2.44 |
| 11.07.2015 | RAS | A | 7.4 | 7.43 | 26.2 | 1.09 |
| 11.07.2015 | RAS | A | 7.4 | 7.43 | 26.2 | 1.09 |
| 11.07.2015 | RAS | A | 7.4 | 7.43 | 26.2 | 1.09 |
| 11.07.2015 | RAS | A | 7.4 | 7.43 | 26.2 | 1.09 |
| 11.07.2015 | RAS | C | 7.2 | 7.43 | 26.9 | 1.6 |
| 11.07.2015 | RAS | C | 7.2 | 7.43 | 26.9 | 1.6 |
| 11.07.2015 | RAS | C | 7.2 | 7.43 | 26.9 | 1.6 |
| 11.07.2015 | RAS | C | 7.2 | 7.43 | 26.9 | 1.6 |
| 11.07.2015 | RAS | D | 7.1 | 7.45 | 26.5 | 1.6 |
| 11.07.2015 | RAS | D | 7.1 | 7.45 | 26.5 | 1.6 |
| 11.07.2015 | RAS | D | 7.1 | 7.45 | 26.5 | 1.6 |
| 11.07.2015 | RAS | D | 7.1 | 7.45 | 26.5 | 1.6 |
| 12.07.2015 | Hydro | D | 8.07 | 7.8 | 24.5 | 2.62 |
| 12.07.2015 | RAS | A | 7.2 | 7.55 | 26.5 | 1.11 |
| 12.07.2015 | RAS | A | 7.2 | 7.55 | 26.5 | 1.11 |
| 12.07.2015 | RAS | A | 7.2 | 7.55 | 26.5 | 1.11 |
| 12.07.2015 | RAS | A | 7.2 | 7.55 | 26.5 | 1.11 |
| 12.07.2015 | RAS | C | 7.3 | 7.44 | 26.5 | 1.7 |
| 12.07.2015 | RAS | C | 7.3 | 7.44 | 26.5 | 1.7 |
| 12.07.2015 | RAS | C | 7.3 | 7.44 | 26.5 | 1.7 |
| 12.07.2015 | RAS | C | 7.3 | 7.44 | 26.5 | 1.7 |
| 12.07.2015 | RAS | D | 7.1 | 7.44 | 26.9 | 1.6 |
| 12.07.2015 | RAS | D | 7.1 | 7.44 | 26.9 | 1.6 |
| 12.07.2015 | RAS | D | 7.1 | 7.44 | 26.9 | 1.6 |
| 12.07.2015 | RAS | D | 7.1 | 7.44 | 26.9 | 1.6 |
| 13.07.2015 | Hydro | D | 8.13 | 7.628 | 24.7 | 2.91 |
| 13.07.2015 | RAS | A | 4.4 | 7.5 | 17 | 1.13 |
| 13.07.2015 | RAS | A | 5.79 | 7.5 | 17 | 1.13 |
| 13.07.2015 | RAS | A | 6.27 | 7.5 | 17 | 1.13 |
| 13.07.2015 | RAS | A | 4.9 | 7.5 | 17 | 1.13 |
| 13.07.2015 | RAS | C | 4.4 | 7.29 | 26.7 | 1.7 |
| 13.07.2015 | RAS | C | 6.66 | 7.29 | 26.7 | 1.7 |
| 13.07.2015 | RAS | C | 5.41 | 7.29 | 26.7 | 1.7 |
| 13.07.2015 | RAS | C | 4.5 | 7.29 | 26.7 | 1.7 |
| 13.07.2015 | RAS | D |  | 7.33 | 27.4 | 1.6 |
| 13.07.2015 | RAS | D | 5.1 | 7.33 | 27.4 | 1.6 |
| 13.07.2015 | RAS | D | 5.6 | 7.33 | 27.4 | 1.6 |
| 13.07.2015 | RAS | D | 4.9 | 7.33 | 27.4 | 1.6 |
| 14.07.2015 | Hydro | D | 8.06 | 6.6 | 25 | 3.25 |
| 14.07.2015 | RAS | A | 7.3 | 7.37 | 27.2 | 1.141 |
| 14.07.2015 | RAS | A | 4.5 | 7.37 | 27.2 | 1.141 |
| 14.07.2015 | RAS | A | 6.26 | 7.37 | 27.2 | 1.141 |
| 14.07.2015 | RAS | A | 4.7 | 7.37 | 27.2 | 1.141 |
| 14.07.2015 | RAS | C | 4.7 | 7.25 | 26.2 | 1.6 |
| 14.07.2015 | RAS | C | 6.53 | 7.25 | 26.2 | 1.6 |
| 14.07.2015 | RAS | C | 5.54 | 7.25 | 26.2 | 1.6 |
| 14.07.2015 | RAS | C | 4.3 | 7.25 | 26.2 | 1.6 |
| 14.07.2015 | RAS | D | 7.6 | 7.31 | 27.1 | 1.6 |
| 14.07.2015 | RAS | D | 5.12 | 7.31 | 27.1 | 1.6 |
| 14.07.2015 | RAS | D | 5.88 | 7.31 | 27.1 | 1.6 |
| 14.07.2015 | RAS | D | 5 | 7.31 | 27.1 | 1.6 |
| 15.07.2015 | Hydro | D | 8.32 | 6.59 | 24.3 | 2.184 |
| 15.07.2015 | RAS | A | 7.7 | 7.75 | 25.1 | 1.143 |
| 15.07.2015 | RAS | A | 5.4 | 7.75 | 25.1 | 1.143 |
| 15.07.2015 | RAS | A | 7.14 | 7.75 | 25.1 | 1.143 |
| 15.07.2015 | RAS | A | 5.4 | 7.75 | 25.1 | 1.143 |
| 15.07.2015 | RAS | C | 4.3 | 7.23 | 26.4 | 1.7 |
| 15.07.2015 | RAS | C | 6.54 | 7.23 | 26.4 | 1.7 |
| 15.07.2015 | RAS | C | 5.55 | 7.23 | 26.4 | 1.7 |
| 15.07.2015 | RAS | C | 5.82 | 7.23 | 26.4 | 1.7 |
| 15.07.2015 | RAS | D | 8.2 | 7.27 | 27.3 | 1.7 |
| 15.07.2015 | RAS | D | 5.39 | 7.27 | 27.3 | 1.7 |
| 15.07.2015 | RAS | D | 5.6 | 7.27 | 27.3 | 1.7 |
| 15.07.2015 | RAS | D | 4.8 | 7.27 | 27.3 | 1.7 |
| 16.07.2015 | Hydro | D | 8.18 | 6.5 | 24.9 | 2.3 |
| 16.07.2015 | RAS | A | 7.1 | 7.73 | 26.7 |  |
| 16.07.2015 | RAS | A | 4.5 | 7.73 | 26.7 |  |
| 16.07.2015 | RAS | A | 6.29 | 7.73 | 26.7 |  |
| 16.07.2015 | RAS | A | 5.2 | 7.73 | 26.7 |  |
| 16.07.2015 | RAS | C | 8.7 | 7.01 | 26.6 | 1.7 |
| 16.07.2015 | RAS | C |  | 7.01 | 26.6 | 1.7 |
| 16.07.2015 | RAS | C | 4.7 | 7.01 | 26.6 | 1.7 |
| 16.07.2015 | RAS | C | 5 | 7.01 | 26.6 | 1.7 |
| 16.07.2015 | RAS | D | 7.1 | 7.07 | 27.4 | 1.7 |
| 16.07.2015 | RAS | D | 4.1 | 7.07 | 27.4 | 1.7 |
| 16.07.2015 | RAS | D | 4.8 | 7.07 | 27.4 | 1.7 |
| 16.07.2015 | RAS | D | 4.8 | 7.07 | 27.4 | 1.7 |
| 17.07.2015 | Hydro | D | 7.92 | 5.8 | 25.4 | 2.52 |
| 17.07.2015 | RAS | A | 6.5 | 7.72 | 27.3 | 1.028 |
| 17.07.2015 | RAS | A | 5.3 | 7.72 | 27.3 | 1.028 |
| 17.07.2015 | RAS | A | 6.05 | 7.72 | 27.3 | 1.028 |
| 17.07.2015 | RAS | A | 5.2 | 7.72 | 27.3 | 1.028 |
| 17.07.2015 | RAS | C | 8.2 | 7.02 | 27.1 | 1.7 |
| 17.07.2015 | RAS | C | 5.95 | 7.02 | 27.1 | 1.7 |
| 17.07.2015 | RAS | C | 4.5 | 7.02 | 27.1 | 1.7 |
| 17.07.2015 | RAS | C | 4.7 | 7.02 | 27.1 | 1.7 |
| 17.07.2015 | RAS | D | 6.9 | 7.09 | 27.9 | 1.7 |
| 17.07.2015 | RAS | D | 4.2 | 7.09 | 27.9 | 1.7 |
| 17.07.2015 | RAS | D | 5.3 | 7.09 | 27.9 | 1.7 |
| 17.07.2015 | RAS | D | 4.6 | 7.09 | 27.9 | 1.7 |
| 18.07.2015 | Hydro | D | 8.01 |  | 25.9 | 2.82 |
| 18.07.2015 | RAS | A | 4.8 | 7.57 | 28.4 |  |
| 18.07.2015 | RAS | A | 4.8 | 7.57 | 28.4 |  |
| 18.07.2015 | RAS | A | 4.8 | 7.57 | 28.4 |  |
| 18.07.2015 | RAS | A | 4.8 | 7.57 | 28.4 |  |
| 18.07.2015 | RAS | C | 6.8 | 7.17 | 27.9 | 1.7 |
| 18.07.2015 | RAS | C | 6.8 | 7.17 | 27.9 | 1.7 |
| 18.07.2015 | RAS | C | 6.8 | 7.17 | 27.9 | 1.7 |
| 18.07.2015 | RAS | C | 6.8 | 7.17 | 27.9 | 1.7 |
| 18.07.2015 | RAS | D | 6.3 | 7.05 | 28.7 | 1.7 |
| 18.07.2015 | RAS | D | 6.3 | 7.05 | 28.7 | 1.7 |
| 18.07.2015 | RAS | D | 6.3 | 7.05 | 28.7 | 1.7 |
| 18.07.2015 | RAS | D | 6.3 | 7.05 | 28.7 | 1.7 |
| 19.07.2015 | Hydro | D |  | 6.85 |  | 3.32 |
| 19.07.2015 | RAS | A | 5.9 | 7.56 | 28.6 |  |
| 19.07.2015 | RAS | A | 5.9 | 7.56 | 28.6 |  |
| 19.07.2015 | RAS | A | 5.9 | 7.56 | 28.6 |  |
| 19.07.2015 | RAS | A | 5.9 | 7.56 | 28.6 |  |
| 19.07.2015 | RAS | C | 6.8 | 7.11 | 28.2 | 1.7 |
| 19.07.2015 | RAS | C | 6.8 | 7.11 | 28.2 | 1.7 |
| 19.07.2015 | RAS | C | 6.8 | 7.11 | 28.2 | 1.7 |
| 19.07.2015 | RAS | C | 6.8 | 7.11 | 28.2 | 1.7 |
| 19.07.2015 | RAS | D | 6.2 | 7.04 | 29 | 1.8 |
| 19.07.2015 | RAS | D | 6.2 | 7.04 | 29 | 1.8 |
| 19.07.2015 | RAS | D | 6.2 | 7.04 | 29 | 1.8 |
| 19.07.2015 | RAS | D | 6.2 | 7.04 | 29 | 1.8 |
| 20.07.2015 | Hydro | D | 8.23 | 5.1 | 24.5 | 3.79 |
| 20.07.2015 | RAS | A | 6.5 | 7.49 | 28.1 | 1.065 |
| 20.07.2015 | RAS | A | 5.1 | 7.49 | 28.1 | 1.065 |
| 20.07.2015 | RAS | A | 6.28 | 7.49 | 28.1 | 1.065 |
| 20.07.2015 | RAS | A | 4.9 | 7.49 | 28.1 | 1.065 |
| 20.07.2015 | RAS | C | 8 | 6.9 | 27.4 | 1.7 |
| 20.07.2015 | RAS | C | 6.43 | 6.9 | 27.4 | 1.7 |
| 20.07.2015 | RAS | C | 4.4 | 6.9 | 27.4 | 1.7 |
| 20.07.2015 | RAS | C | 4.9 | 6.9 | 27.4 | 1.7 |
| 20.07.2015 | RAS | D | 6.8 | 7.05 | 28.4 | 1.8 |
| 20.07.2015 | RAS | D | 4 | 7.05 | 28.4 | 1.8 |
| 20.07.2015 | RAS | D | 5.3 | 7.05 | 28.4 | 1.8 |
| 20.07.2015 | RAS | D | 5.51 | 7.05 | 28.4 | 1.8 |
| 21.07.2015 | Hydro | D | 7.84 | 6.18 | 25.9 | 4.73 |
| 21.07.2015 | RAS | A | 6.4 | 7.26 | 28.2 |  |
| 21.07.2015 | RAS | A | 4.6 | 7.26 | 28.2 |  |
| 21.07.2015 | RAS | A | 6.34 | 7.26 | 28.2 |  |
| 21.07.2015 | RAS | A | 4.7 | 7.26 | 28.2 |  |
| 21.07.2015 | RAS | C | 8 | 7.17 | 27.1 | 1.7 |
| 21.07.2015 | RAS | C | 6.31 | 7.17 | 27.1 | 1.7 |
| 21.07.2015 | RAS | C | 4.6 | 7.17 | 27.1 | 1.7 |
| 21.07.2015 | RAS | C | 4.8 | 7.17 | 27.1 | 1.7 |
| 21.07.2015 | RAS | D | 6.7 | 7.18 | 27.9 | 1.7 |
| 21.07.2015 | RAS | D | 4.3 | 7.18 | 27.9 | 1.7 |
| 21.07.2015 | RAS | D | 4.9 | 7.18 | 27.9 | 1.7 |
| 21.07.2015 | RAS | D | 5.72 | 7.18 | 27.9 | 1.7 |
| 22.07.2015 | Hydro | D | 8 | 6.91 | 25.5 | 3.49 |
| 22.07.2015 | RAS | A | 6.5 | 7.31 | 28.4 | 1.089 |
| 22.07.2015 | RAS | A | 4.7 | 7.31 | 28.4 | 1.089 |
| 22.07.2015 | RAS | A | 6.27 | 7.31 | 28.4 | 1.089 |
| 22.07.2015 | RAS | A | 4.5 | 7.31 | 28.4 | 1.089 |
| 22.07.2015 | RAS | C | 7.9 | 7.3 | 27.5 | 1.8 |
| 22.07.2015 | RAS | C | 6.39 | 7.3 | 27.5 | 1.8 |
| 22.07.2015 | RAS | C | 4.2 | 7.3 | 27.5 | 1.8 |
| 22.07.2015 | RAS | C | 4.5 | 7.3 | 27.5 | 1.8 |
| 22.07.2015 | RAS | D | 6.7 | 7.18 | 28.3 | 1.8 |
| 22.07.2015 | RAS | D | 5.43 | 7.18 | 28.3 | 1.8 |
| 22.07.2015 | RAS | D | 4.8 | 7.18 | 28.3 | 1.8 |
| 22.07.2015 | RAS | D | 5.37 | 7.18 | 28.3 | 1.8 |
| 23.07.2015 | Hydro | D | 8.16 | 7.26 | 25.2 | 4.11 |
| 23.07.2015 | RAS | A | 6.4 | 7.46 | 28.7 | 1.1 |
| 23.07.2015 | RAS | A | 4.6 | 7.46 | 28.7 | 1.1 |
| 23.07.2015 | RAS | A |  | 7.46 | 28.7 | 1.1 |
| 23.07.2015 | RAS | A | 4.1 | 7.46 | 28.7 | 1.1 |
| 23.07.2015 | RAS | C | 7.8 | 7.41 | 28 | 1.8 |
| 23.07.2015 | RAS | C |  | 7.41 | 28 | 1.8 |
| 23.07.2015 | RAS | C | 4 | 7.41 | 28 | 1.8 |
| 23.07.2015 | RAS | C | 4.4 | 7.41 | 28 | 1.8 |
| 23.07.2015 | RAS | D | 6.6 | 7.61 | 28.7 | 1.8 |
| 23.07.2015 | RAS | D |  | 7.61 | 28.7 | 1.8 |
| 23.07.2015 | RAS | D | 4.4 | 7.61 | 28.7 | 1.8 |
| 23.07.2015 | RAS | D |  | 7.61 | 28.7 | 1.8 |
| 24.07.2015 | Hydro | D | 8.34 | 6.11 | 24.7 | 5.08 |
| 24.07.2015 | RAS | A | 6.5 | 7.16 | 28.2 | 1.061 |
| 24.07.2015 | RAS | A | 4.7 | 7.16 | 28.2 | 1.061 |
| 24.07.2015 | RAS | A | 6.44 | 7.16 | 28.2 | 1.061 |
| 24.07.2015 | RAS | A | 4.3 | 7.16 | 28.2 | 1.061 |
| 24.07.2015 | RAS | C | 7.9 | 7.36 | 27.6 | 1.7 |
| 24.07.2015 | RAS | C | 6.35 | 7.36 | 27.6 | 1.7 |
| 24.07.2015 | RAS | C | 5.24 | 7.36 | 27.6 | 1.7 |
| 24.07.2015 | RAS | C | 4.6 | 7.36 | 27.6 | 1.7 |
| 24.07.2015 | RAS | D | 6.5 | 7.49 | 28.2 | 1.8 |
| 24.07.2015 | RAS | D | 5.54 | 7.49 | 28.2 | 1.8 |
| 24.07.2015 | RAS | D | 4.6 | 7.49 | 28.2 | 1.8 |
| 24.07.2015 | RAS | D | 5.41 | 7.49 | 28.2 | 1.8 |
| 25.07.2015 | Hydro | D | 7.88 | 7.17 | 25.5 | 2.37 |
| 25.07.2015 | RAS | A | 5.5 | 7.37 | 28.2 | 1.155 |
| 25.07.2015 | RAS | A | 5.5 | 7.37 | 28.2 | 1.155 |
| 25.07.2015 | RAS | A | 5.5 | 7.37 | 28.2 | 1.155 |
| 25.07.2015 | RAS | A | 5.5 | 7.37 | 28.2 | 1.155 |
| 25.07.2015 | RAS | C | 6.7 | 6.99 | 27.8 | 1.8 |
| 25.07.2015 | RAS | C | 6.7 | 6.99 | 27.8 | 1.8 |
| 25.07.2015 | RAS | C | 6.7 | 6.99 | 27.8 | 1.8 |
| 25.07.2015 | RAS | C | 6.7 | 6.99 | 27.8 | 1.8 |
| 25.07.2015 | RAS | D | 6.8 | 7.17 | 28.5 | 1.8 |
| 25.07.2015 | RAS | D | 6.8 | 7.17 | 28.5 | 1.8 |
| 25.07.2015 | RAS | D | 6.8 | 7.17 | 28.5 | 1.8 |
| 25.07.2015 | RAS | D | 6.8 | 7.17 | 28.5 | 1.8 |
| 26.07.2015 | Hydro | D | 8.31 | 7.05 | 25.4 | 2.51 |
| 26.07.2015 | RAS | A | 6 | 7.09 | 27.7 | 1.166 |
| 26.07.2015 | RAS | A | 6 | 7.09 | 27.7 | 1.166 |
| 26.07.2015 | RAS | A | 6 | 7.09 | 27.7 | 1.166 |
| 26.07.2015 | RAS | A | 6 | 7.09 | 27.7 | 1.166 |
| 26.07.2015 | RAS | C | 6.9 | 6.64 | 27.2 | 1.8 |
| 26.07.2015 | RAS | C | 6.9 | 6.64 | 27.2 | 1.8 |
| 26.07.2015 | RAS | C | 6.9 | 6.64 | 27.2 | 1.8 |
| 26.07.2015 | RAS | C | 6.9 | 6.64 | 27.2 | 1.8 |
| 26.07.2015 | RAS | D | 7 | 7.25 | 28 | 1.8 |
| 26.07.2015 | RAS | D | 7 | 7.25 | 28 | 1.8 |
| 26.07.2015 | RAS | D | 7 | 7.25 | 28 | 1.8 |
| 26.07.2015 | RAS | D | 7 | 7.25 | 28 | 1.8 |
| 27.07.2015 | Hydro | D | 8.17 | 7.63 | 24.6 | 2.73 |
| 27.07.2015 | RAS | A | 6 |  | 27.2 | 1.165 |
| 27.07.2015 | RAS | A | 6 |  | 27.2 | 1.165 |
| 27.07.2015 | RAS | A | 6 |  | 27.2 | 1.165 |
| 27.07.2015 | RAS | A | 6 |  | 27.2 | 1.165 |
| 27.07.2015 | RAS | C | 6.7 |  | 26.6 | 1.8 |
| 27.07.2015 | RAS | C | 6.7 |  | 26.6 | 1.8 |
| 27.07.2015 | RAS | C | 6.7 |  | 26.6 | 1.8 |
| 27.07.2015 | RAS | C | 6.7 |  | 26.6 | 1.8 |
| 27.07.2015 | RAS | D | 7.1 |  | 27.4 | 1.9 |
| 27.07.2015 | RAS | D | 7.1 |  | 27.4 | 1.9 |
| 27.07.2015 | RAS | D | 7.1 |  | 27.4 | 1.9 |
| 27.07.2015 | RAS | D | 7.1 |  | 27.4 | 1.9 |
| 28.07.2015 | Hydro | D | 8.18 | 5.8 | 24 | 3.08 |
| 28.07.2015 | RAS | A | 5.9 | 7.25 | 26.9 |  |
| 28.07.2015 | RAS | A | 5.9 | 7.25 | 26.9 |  |
| 28.07.2015 | RAS | A | 5.9 | 7.25 | 26.9 |  |
| 28.07.2015 | RAS | A | 5.9 | 7.25 | 26.9 |  |
| 28.07.2015 | RAS | C | 7 | 7.17 | 26.2 | 1.7 |
| 28.07.2015 | RAS | C | 7 | 7.17 | 26.2 | 1.7 |
| 28.07.2015 | RAS | C | 7 | 7.17 | 26.2 | 1.7 |
| 28.07.2015 | RAS | C | 7 | 7.17 | 26.2 | 1.7 |
| 28.07.2015 | RAS | D | 7.2 | 7.16 | 26.7 | 1.8 |
| 28.07.2015 | RAS | D | 7.2 | 7.16 | 26.7 | 1.8 |
| 28.07.2015 | RAS | D | 7.2 | 7.16 | 26.7 | 1.8 |
| 28.07.2015 | RAS | D | 7.2 | 7.16 | 26.7 | 1.8 |
| 29.07.2015 | Hydro | D | 8.07 | 6.96 | 25.4 | 2.79 |
| 29.07.2015 | RAS | A | 8 | 7.26 | 27.2 |  |
| 29.07.2015 | RAS | A | 6.39 | 7.26 | 27.2 |  |
| 29.07.2015 | RAS | A | 6.33 | 7.26 | 27.2 |  |
| 29.07.2015 | RAS | A | 6.16 | 7.26 | 27.2 |  |
| 29.07.2015 | RAS | C | 7.89 | 7.05 | 26.6 | 1.9 |
| 29.07.2015 | RAS | C | 6.39 | 7.05 | 26.6 | 1.9 |
| 29.07.2015 | RAS | C | 5.4 | 7.05 | 26.6 | 1.9 |
| 29.07.2015 | RAS | C | 5.88 | 7.05 | 26.6 | 1.9 |
| 29.07.2015 | RAS | D | 7.91 | 7.03 | 27.2 | 1.9 |
| 29.07.2015 | RAS | D | 5.44 | 7.03 | 27.2 | 1.9 |
| 29.07.2015 | RAS | D | 5.35 | 7.03 | 27.2 | 1.9 |
| 29.07.2015 | RAS | D | 6.37 | 7.03 | 27.2 | 1.9 |
| 30.07.2015 | Hydro | D | 8.25 | 7.06 | 24.6 | 3.07 |
| 30.07.2015 | RAS | A | 8.12 | 7.44 | 26.9 | 1.24 |
| 30.07.2015 | RAS | A | 6.28 | 7.44 | 26.9 | 1.24 |
| 30.07.2015 | RAS | A | 8.12 | 7.44 | 26.9 | 1.24 |
| 30.07.2015 | RAS | A | 5.98 | 7.44 | 26.9 | 1.24 |
| 30.07.2015 | RAS | C | 7.97 | 7.18 | 26.2 | 1.9 |
| 30.07.2015 | RAS | C | 6.3 | 7.18 | 26.2 | 1.9 |
| 30.07.2015 | RAS | C | 5.31 | 7.18 | 26.2 | 1.9 |
| 30.07.2015 | RAS | C | 5.66 | 7.18 | 26.2 | 1.9 |
| 30.07.2015 | RAS | D | 8.08 | 7.18 | 27 | 1.9 |
| 30.07.2015 | RAS | D | 5.26 | 7.18 | 27 | 1.9 |
| 30.07.2015 | RAS | D | 5.41 | 7.18 | 27 | 1.9 |
| 30.07.2015 | RAS | D | 6.13 | 7.18 | 27 | 1.9 |
| 31.07.2015 | Hydro | D | 8.35 | 5.87 | 23.8 | 3.3 |
| 31.07.2015 | RAS | A | 7.2 | 7.19 | 26.4 |  |
| 31.07.2015 | RAS | A | 7.2 | 7.19 | 26.4 |  |
| 31.07.2015 | RAS | A | 7.2 | 7.19 | 26.4 |  |
| 31.07.2015 | RAS | A | 7.2 | 7.19 | 26.4 |  |
| 31.07.2015 | RAS | C | 7.4 | 7.28 | 26.2 | 2 |
| 31.07.2015 | RAS | C | 7.4 | 7.28 | 26.2 | 2 |
| 31.07.2015 | RAS | C | 7.4 | 7.28 | 26.2 | 2 |
| 31.07.2015 | RAS | C | 7.4 | 7.28 | 26.2 | 2 |
| 31.07.2015 | RAS | D | 5.8 | 7.24 | 26.7 | 1.9 |
| 31.07.2015 | RAS | D | 5.8 | 7.24 | 26.7 | 1.9 |
| 31.07.2015 | RAS | D | 5.8 | 7.24 | 26.7 | 1.9 |
| 31.07.2015 | RAS | D | 5.8 | 7.24 | 26.7 | 1.9 |
| 01.08.2015 | Hydro | D | 8.45 |  | 23.4 | 3.06 |
| 01.08.2015 | RAS | A | 7.5 | 7.25 | 26 | 1.26 |
| 01.08.2015 | RAS | A | 7.5 | 7.25 | 26 | 1.26 |
| 01.08.2015 | RAS | A | 7.5 | 7.25 | 26 | 1.26 |
| 01.08.2015 | RAS | A | 7.5 | 7.25 | 26 | 1.26 |
| 01.08.2015 | RAS | C | 7.4 | 7.25 | 26.3 | 2 |
| 01.08.2015 | RAS | C | 7.4 | 7.25 | 26.3 | 2 |
| 01.08.2015 | RAS | C | 7.4 | 7.25 | 26.3 | 2 |
| 01.08.2015 | RAS | C | 7.4 | 7.25 | 26.3 | 2 |
| 01.08.2015 | RAS | D | 6 | 7.22 | 26.3 | 2 |
| 01.08.2015 | RAS | D | 6 | 7.22 | 26.3 | 2 |
| 01.08.2015 | RAS | D | 6 | 7.22 | 26.3 | 2 |
| 01.08.2015 | RAS | D | 6 | 7.22 | 26.3 | 2 |
| 02.08.2015 | Hydro | D | 8.28 |  | 24.4 | 3.34 |
| 02.08.2015 | RAS | A | 7.4 | 6.98 | 26.9 |  |
| 02.08.2015 | RAS | A | 7.4 | 6.98 | 26.9 |  |
| 02.08.2015 | RAS | A | 7.4 | 6.98 | 26.9 |  |
| 02.08.2015 | RAS | A | 7.4 | 6.98 | 26.9 |  |
| 02.08.2015 | RAS | C | 7.3 | 7.16 | 26.8 | 2 |
| 02.08.2015 | RAS | C | 7.3 | 7.16 | 26.8 | 2 |
| 02.08.2015 | RAS | C | 7.3 | 7.16 | 26.8 | 2 |
| 02.08.2015 | RAS | C | 7.3 | 7.16 | 26.8 | 2 |
| 02.08.2015 | RAS | D | 5.8 | 7.17 | 27.2 | 2 |
| 02.08.2015 | RAS | D | 5.8 | 7.17 | 27.2 | 2 |
| 02.08.2015 | RAS | D | 5.8 | 7.17 | 27.2 | 2 |
| 02.08.2015 | RAS | D | 5.8 | 7.17 | 27.2 | 2 |
| 03.08.2015 | Hydro | D | 8.29 | 6.91 | 24.5 | 4.07 |
| 03.08.2015 | RAS | A | 8.1 | 7.02 | 27.7 | 1.274 |
| 03.08.2015 | RAS | A | 5.6 | 7.02 | 27.7 | 1.274 |
| 03.08.2015 | RAS | A | 5.51 | 7.02 | 27.7 | 1.274 |
| 03.08.2015 | RAS | A | 5.23 | 7.02 | 27.7 | 1.274 |
| 03.08.2015 | RAS | C |  | 7.14 | 27.5 | 2 |
| 03.08.2015 | RAS | C | 4.99 | 7.14 | 27.5 | 2 |
| 03.08.2015 | RAS | C | 6.6 | 7.14 | 27.5 | 2 |
| 03.08.2015 | RAS | C | 5.28 | 7.14 | 27.5 | 2 |
| 03.08.2015 | RAS | D | 7.8 | 7.15 | 27.9 | 2 |
| 03.08.2015 | RAS | D | 4.57 | 7.15 | 27.9 | 2 |
| 03.08.2015 | RAS | D | 5.5 | 7.15 | 27.9 | 2 |
| 03.08.2015 | RAS | D | 5.2 | 7.15 | 27.9 | 2 |
| 04.08.2015 | Hydro | D | 8.1 | 5.56 | 25.4 | 4.83 |
| 04.08.2015 | RAS | A | 7.9 | 7.66 | 26.4 | 1.079 |
| 04.08.2015 | RAS | A | 6.6 | 7.66 | 26.4 | 1.079 |
| 04.08.2015 | RAS | A | 6.5 | 7.66 | 26.4 | 1.079 |
| 04.08.2015 | RAS | A | 6.21 | 7.66 | 26.4 | 1.079 |
| 04.08.2015 | RAS | C |  | 6.99 | 27.5 | 1.9 |
| 04.08.2015 | RAS | C | 6.09 | 6.99 | 27.5 | 1.9 |
| 04.08.2015 | RAS | C | 7.9 | 6.99 | 27.5 | 1.9 |
| 04.08.2015 | RAS | C | 5.72 | 6.99 | 27.5 | 1.9 |
| 04.08.2015 | RAS | D | 7.7 | 7.04 | 27.8 | 1.9 |
| 04.08.2015 | RAS | D | 5.36 | 7.04 | 27.8 | 1.9 |
| 04.08.2015 | RAS | D | 6.8 | 7.04 | 27.8 | 1.9 |
| 04.08.2015 | RAS | D | 6.28 | 7.04 | 27.8 | 1.9 |
| 05.08.2015 | Hydro | D | 8.17 | 6.65 | 25.2 |  |
| 05.08.2015 | RAS | A | 7.6 | 7.51 | 27.8 | 1.084 |
| 05.08.2015 | RAS | A | 5.9 | 7.51 | 27.8 | 1.084 |
| 05.08.2015 | RAS | A | 6.3 | 7.51 | 27.8 | 1.084 |
| 05.08.2015 | RAS | A | 6.04 | 7.51 | 27.8 | 1.084 |
| 05.08.2015 | RAS | C |  | 7.36 | 26.4 | 1.5 |
| 05.08.2015 | RAS | C | 6.25 | 7.36 | 26.4 | 1.5 |
| 05.08.2015 | RAS | C | 7.7 | 7.36 | 26.4 | 1.5 |
| 05.08.2015 | RAS | C | 6.25 | 7.36 | 26.4 | 1.5 |
| 05.08.2015 | RAS | D | 7.6 | 7.45 | 26.4 | 1.5 |
| 05.08.2015 | RAS | D | 5.65 | 7.45 | 26.4 | 1.5 |
| 05.08.2015 | RAS | D | 6.8 | 7.45 | 26.4 | 1.5 |
| 05.08.2015 | RAS | D | 6.62 | 7.45 | 26.4 | 1.5 |
| 06.08.2015 | Hydro | D | 7.91 | 6.07 | 25.4 | 2.37 |
| 06.08.2015 | RAS | A | 7.2 | 7.35 | 27.8 | 1.087 |
| 06.08.2015 | RAS | A | 5 | 7.35 | 27.8 | 1.087 |
| 06.08.2015 | RAS | A | 5.59 | 7.35 | 27.8 | 1.087 |
| 06.08.2015 | RAS | A | 5.44 | 7.35 | 27.8 | 1.087 |
| 06.08.2015 | RAS | C | 8.7 | 7.17 | 27.2 | 1.6 |
| 06.08.2015 | RAS | C | 5.71 | 7.17 | 27.2 | 1.6 |
| 06.08.2015 | RAS | C | 5.4 | 7.17 | 27.2 | 1.6 |
| 06.08.2015 | RAS | C | 5.23 | 7.17 | 27.2 | 1.6 |
| 06.08.2015 | RAS | D | 7.7 | 7.25 | 27.4 | 1.5 |
| 06.08.2015 | RAS | D | 5 | 7.25 | 27.4 | 1.5 |
| 06.08.2015 | RAS | D | 6 | 7.25 | 27.4 | 1.5 |
| 06.08.2015 | RAS | D | 5.64 | 7.25 | 27.4 | 1.5 |
| 07.08.2015 | Hydro | D | 7.85 | 6.13 | 26.6 | 2.64 |
| 07.08.2015 | RAS | A | 5.8 | 7.46 | 28.8 | 1.061 |
| 07.08.2015 | RAS | A | 5.3 | 7.46 | 28.8 | 1.061 |
| 07.08.2015 | RAS | A | 6.23 | 7.46 | 28.8 | 1.061 |
| 07.08.2015 | RAS | A | 7.82 | 7.46 | 28.8 | 1.061 |
| 07.08.2015 | RAS | C | 6.6 | 7.24 | 28.6 | 1.5 |
| 07.08.2015 | RAS | C | 5.94 | 7.24 | 28.6 | 1.5 |
| 07.08.2015 | RAS | C | 6.9 | 7.24 | 28.6 | 1.5 |
| 07.08.2015 | RAS | C | 7.71 | 7.24 | 28.6 | 1.5 |
| 07.08.2015 | RAS | D | 5.9 | 7.26 | 29.1 | 1.5 |
| 07.08.2015 | RAS | D | 5.72 | 7.26 | 29.1 | 1.5 |
| 07.08.2015 | RAS | D | 6.2 | 7.26 | 29.1 | 1.5 |
| 07.08.2015 | RAS | D | 7.77 | 7.26 | 29.1 | 1.5 |
| 08.08.2015 | Hydro | D | 7.83 | 6.97 | 26.9 | 3.17 |
| 08.08.2015 | RAS | A | 6.2 | 7.43 | 29.3 | 1.089 |
| 08.08.2015 | RAS | A | 6.2 | 7.43 | 29.3 | 1.089 |
| 08.08.2015 | RAS | A | 6.2 | 7.43 | 29.3 | 1.089 |
| 08.08.2015 | RAS | A | 6.2 | 7.43 | 29.3 | 1.089 |
| 08.08.2015 | RAS | C | 6.8 | 7.22 | 29.1 | 1.6 |
| 08.08.2015 | RAS | C | 6.8 | 7.22 | 29.1 | 1.6 |
| 08.08.2015 | RAS | C | 6.8 | 7.22 | 29.1 | 1.6 |
| 08.08.2015 | RAS | C | 6.8 | 7.22 | 29.1 | 1.6 |
| 08.08.2015 | RAS | D | 6.5 | 7.27 | 29.6 | 1.6 |
| 08.08.2015 | RAS | D | 6.5 | 7.27 | 29.6 | 1.6 |
| 08.08.2015 | RAS | D | 6.5 | 7.27 | 29.6 | 1.6 |
| 08.08.2015 | RAS | D | 6.5 | 7.27 | 29.6 | 1.6 |
| 09.08.2015 | Hydro | D | 8.11 | 7.03 | 25.7 | 3.94 |
| 09.08.2015 | RAS | A | 6.1 | 7.19 | 29.3 | 1.107 |
| 09.08.2015 | RAS | A | 6.1 | 7.19 | 29.3 | 1.107 |
| 09.08.2015 | RAS | A | 6.1 | 7.19 | 29.3 | 1.107 |
| 09.08.2015 | RAS | A | 6.1 | 7.19 | 29.3 | 1.107 |
| 09.08.2015 | RAS | C | 6.7 | 7.25 | 29.1 | 1.6 |
| 09.08.2015 | RAS | C | 6.7 | 7.25 | 29.1 | 1.6 |
| 09.08.2015 | RAS | C | 6.7 | 7.25 | 29.1 | 1.6 |
| 09.08.2015 | RAS | C | 6.7 | 7.25 | 29.1 | 1.6 |
| 09.08.2015 | RAS | D | 6.2 | 6.95 | 29.5 | 1.6 |
| 09.08.2015 | RAS | D | 6.2 | 6.95 | 29.5 | 1.6 |
| 09.08.2015 | RAS | D | 6.2 | 6.95 | 29.5 | 1.6 |
| 09.08.2015 | RAS | D | 6.2 | 6.95 | 29.5 | 1.6 |
| 10.08.2015 | Hydro | D | 8.03 | 7.17 | 26 | 5.02 |
| 10.08.2015 | RAS | A | 6.05 | 7.19 | 28.9 | 1.123 |
| 10.08.2015 | RAS | A | 5.8 | 7.19 | 28.9 | 1.123 |
| 10.08.2015 | RAS | A | 6.07 | 7.19 | 28.9 | 1.123 |
| 10.08.2015 | RAS | A | 7.85 | 7.19 | 28.9 | 1.123 |
| 10.08.2015 | RAS | C | 5.28 | 7.21 | 28.8 | 1.6 |
| 10.08.2015 | RAS | C | 5.67 | 7.21 | 28.8 | 1.6 |
| 10.08.2015 | RAS | C | 6.1 | 7.21 | 28.8 | 1.6 |
| 10.08.2015 | RAS | C | 7.7 | 7.21 | 28.8 | 1.6 |
| 10.08.2015 | RAS | D | 5.52 | 6.9 | 29.2 | 1.6 |
| 10.08.2015 | RAS | D | 5.7 | 6.9 | 29.2 | 1.6 |
| 10.08.2015 | RAS | D | 6.2 | 6.9 | 29.2 | 1.6 |
| 10.08.2015 | RAS | D | 7.8 | 6.9 | 29.2 | 1.6 |
| 11.08.2015 | Hydro | D | 7.57 | 7.26 | 26.5 | 2.77 |
| 11.08.2015 | RAS | A | 5.8 | 7.22 | 29.1 | 1.154 |
| 11.08.2015 | RAS | A | 5.8 | 7.22 | 29.1 | 1.154 |
| 11.08.2015 | RAS | A | 5.8 | 7.22 | 29.1 | 1.154 |
| 11.08.2015 | RAS | A | 5.8 | 7.22 | 29.1 | 1.154 |
| 11.08.2015 | RAS | C | 6.8 | 7.29 | 28.4 | 1.6 |
| 11.08.2015 | RAS | C | 6.8 | 7.29 | 28.4 | 1.6 |
| 11.08.2015 | RAS | C | 6.8 | 7.29 | 28.4 | 1.6 |
| 11.08.2015 | RAS | C | 6.8 | 7.29 | 28.4 | 1.6 |
| 11.08.2015 | RAS | D | 5.4 | 7.29 | 28.8 | 1.6 |
| 11.08.2015 | RAS | D | 5.4 | 7.29 | 28.8 | 1.6 |
| 11.08.2015 | RAS | D | 5.4 | 7.29 | 28.8 | 1.6 |
| 11.08.2015 | RAS | D | 5.4 | 7.29 | 28.8 | 1.6 |
| 12.08.2015 | Hydro | D | 7.87 | 7.34 | 26.1 | 3.04 |
| 12.08.2015 | RAS | A | 5.43 | 7.14 | 29.4 | 1.179 |
| 12.08.2015 | RAS | A | 4.65 | 7.14 | 29.4 | 1.179 |
| 12.08.2015 | RAS | A | 5.57 | 7.14 | 29.4 | 1.179 |
| 12.08.2015 | RAS | A | 7.84 | 7.14 | 29.4 | 1.179 |
| 12.08.2015 | RAS | C | 4.72 | 7.11 | 28.9 | 1.6 |
| 12.08.2015 | RAS | C | 5.27 | 7.11 | 28.9 | 1.6 |
| 12.08.2015 | RAS | C | 5.24 | 7.11 | 28.9 | 1.6 |
| 12.08.2015 | RAS | C | 7.79 | 7.11 | 28.9 | 1.6 |
| 12.08.2015 | RAS | D | 5.09 | 7.15 | 29.4 | 1.6 |
| 12.08.2015 | RAS | D | 4.76 | 7.15 | 29.4 | 1.6 |
| 12.08.2015 | RAS | D | 5.04 | 7.15 | 29.4 | 1.6 |
| 12.08.2015 | RAS | D | 7.79 | 7.15 | 29.4 | 1.6 |
| 13.08.2015 | Hydro | D | 7.99 | 5.8 | 26.7 | 2.75 |
| 13.08.2015 | RAS | A | 6 | 7.13 | 29.1 | 1.201 |
| 13.08.2015 | RAS | A | 6.12 | 7.13 | 29.1 | 1.201 |
| 13.08.2015 | RAS | A | 6 | 7.13 | 29.1 | 1.201 |
| 13.08.2015 | RAS | A | 7.79 | 7.13 | 29.1 | 1.201 |
| 13.08.2015 | RAS | C | 5.58 | 7.05 | 28.7 | 1.7 |
| 13.08.2015 | RAS | C | 6.05 | 7.05 | 28.7 | 1.7 |
| 13.08.2015 | RAS | C | 5.82 | 7.05 | 28.7 | 1.7 |
| 13.08.2015 | RAS | C | 7.78 | 7.05 | 28.7 | 1.7 |
| 13.08.2015 | RAS | D | 5.66 | 7.12 | 29.2 | 1.7 |
| 13.08.2015 | RAS | D | 5.47 | 7.12 | 29.2 | 1.7 |
| 13.08.2015 | RAS | D | 5.48 | 7.12 | 29.2 | 1.7 |
| 13.08.2015 | RAS | D | 7.78 | 7.12 | 29.2 | 1.7 |
| 14.08.2015 | Hydro | D | 7.71 | 7.02 | 25.7 | 2.95 |
| 14.08.2015 | RAS | A | 5.91 | 7.15 | 28.8 | 1.21 |
| 14.08.2015 | RAS | A | 6.13 | 7.15 | 28.8 | 1.21 |
| 14.08.2015 | RAS | A | 5.95 | 7.15 | 28.8 | 1.21 |
| 14.08.2015 | RAS | A | 7.8 | 7.15 | 28.8 | 1.21 |
| 14.08.2015 | RAS | C | 5.2 | 6.99 | 28.6 | 1.7 |
| 14.08.2015 | RAS | C | 5.74 | 6.99 | 28.6 | 1.7 |
| 14.08.2015 | RAS | C | 5.55 | 6.99 | 28.6 | 1.7 |
| 14.08.2015 | RAS | C | 7.71 | 6.99 | 28.6 | 1.7 |
| 14.08.2015 | RAS | D | 5.41 | 7.03 | 29.1 | 1.7 |
| 14.08.2015 | RAS | D | 5.27 | 7.03 | 29.1 | 1.7 |
| 14.08.2015 | RAS | D | 5.19 | 7.03 | 29.1 | 1.7 |
| 14.08.2015 | RAS | D | 7.7 | 7.03 | 29.1 | 1.7 |
| 15.08.2015 | Hydro | D | 7.81 | 7.31 | 26.5 | 3.24 |
| 15.08.2015 | RAS | A | 5.9 | 7.25 | 29.3 | 1.2 |
| 15.08.2015 | RAS | A | 5.9 | 7.25 | 29.3 | 1.2 |
| 15.08.2015 | RAS | A | 5.9 | 7.25 | 29.3 | 1.2 |
| 15.08.2015 | RAS | A | 5.9 | 7.25 | 29.3 | 1.2 |
| 15.08.2015 | RAS | C | 6.6 | 7.13 | 29.2 | 1.7 |
| 15.08.2015 | RAS | C | 6.6 | 7.13 | 29.2 | 1.7 |
| 15.08.2015 | RAS | C | 6.6 | 7.13 | 29.2 | 1.7 |
| 15.08.2015 | RAS | C | 6.6 | 7.13 | 29.2 | 1.7 |
| 15.08.2015 | RAS | D | 5.9 | 7.31 | 29.6 | 1.7 |
| 15.08.2015 | RAS | D | 5.9 | 7.31 | 29.6 | 1.7 |
| 15.08.2015 | RAS | D | 5.9 | 7.31 | 29.6 | 1.7 |
| 15.08.2015 | RAS | D | 5.9 | 7.31 | 29.6 | 1.7 |
| 16.08.2015 | Hydro | D | 8 | 7.31 | 29.5 | 1.8 |
| 16.08.2015 | RAS | A | 7.88 | 7.63 | 29.1 | 1.2 |
| 16.08.2015 | RAS | A | 7.88 | 7.63 | 29.1 | 1.2 |
| 16.08.2015 | RAS | A | 7.88 | 7.63 | 29.1 | 1.2 |
| 16.08.2015 | RAS | A | 7.88 | 7.63 | 29.1 | 1.2 |
| 16.08.2015 | RAS | C | 7.84 | 7.44 | 29.5 | 1.5 |
| 16.08.2015 | RAS | C | 7.84 | 7.44 | 29.5 | 1.5 |
| 16.08.2015 | RAS | C | 7.84 | 7.44 | 29.5 | 1.5 |
| 16.08.2015 | RAS | C | 7.84 | 7.44 | 29.5 | 1.5 |
| 16.08.2015 | RAS | D | 7.81 | 7.46 | 29.5 | 1.5 |
| 16.08.2015 | RAS | D | 7.81 | 7.46 | 29.5 | 1.5 |
| 16.08.2015 | RAS | D | 7.81 | 7.46 | 29.5 | 1.5 |
| 16.08.2015 | RAS | D | 7.81 | 7.46 | 29.5 | 1.5 |
| 17.08.2015 | Hydro | D | 8.11 | 7.38 | 26.2 | 3.91 |
| 17.08.2015 | RAS | A | 5.24 | 7.11 | 29 | 1.65 |
| 17.08.2015 | RAS | A | 5.65 | 7.11 | 29 | 1.65 |
| 17.08.2015 | RAS | A | 5.23 | 7.11 | 29 | 1.65 |
| 17.08.2015 | RAS | A | 7.82 | 7.11 | 29 | 1.65 |
| 17.08.2015 | RAS | C | 5.28 | 7.06 | 28.8 | 1.8 |
| 17.08.2015 | RAS | C | 4.76 | 7.06 | 28.8 | 1.8 |
| 17.08.2015 | RAS | C | 4.67 | 7.06 | 28.8 | 1.8 |
| 17.08.2015 | RAS | C | 7.81 | 7.06 | 28.8 | 1.8 |
| 17.08.2015 | RAS | D | 5.1 | 7.35 | 29.5 | 1.8 |
| 17.08.2015 | RAS | D | 3.91 | 7.35 | 29.5 | 1.8 |
| 17.08.2015 | RAS | D | 4.1 | 7.35 | 29.5 | 1.8 |
| 17.08.2015 | RAS | D | 7.72 | 7.35 | 29.5 | 1.8 |
| 18.08.2015 | Hydro | D | 8.52 | 7.32 | 24.4 | 4.18 |
| 18.08.2015 | RAS | A | 5.2 | 6.95 | 28.4 | 1.3 |
| 18.08.2015 | RAS | A | 5.2 | 6.95 | 28.4 | 1.3 |
| 18.08.2015 | RAS | A | 5.2 | 6.95 | 28.4 | 1.3 |
| 18.08.2015 | RAS | A | 5.2 | 6.95 | 28.4 | 1.3 |
| 18.08.2015 | RAS | C | 7.1 | 7.1 | 26.7 | 1.7 |
| 18.08.2015 | RAS | C | 7.1 | 7.1 | 26.7 | 1.7 |
| 18.08.2015 | RAS | C | 7.1 | 7.1 | 26.7 | 1.7 |
| 18.08.2015 | RAS | C | 7.1 | 7.1 | 26.7 | 1.7 |
| 18.08.2015 | RAS | D | 6.7 | 7.2 | 27.4 | 1.7 |
| 18.08.2015 | RAS | D | 6.7 | 7.2 | 27.4 | 1.7 |
| 18.08.2015 | RAS | D | 6.7 | 7.2 | 27.4 | 1.7 |
| 18.08.2015 | RAS | D | 6.7 | 7.2 | 27.4 | 1.7 |
| 19.08.2015 | Hydro | D | 8.21 | 5.56 | 26.1 | 4.46 |
| 19.08.2015 | RAS | A | 6.2 | 7.25 | 28.2 | 1.3 |
| 19.08.2015 | RAS | A | 6.2 | 7.25 | 28.2 | 1.3 |
| 19.08.2015 | RAS | A | 6.2 | 7.25 | 28.2 | 1.3 |
| 19.08.2015 | RAS | A | 6.2 | 7.25 | 28.2 | 1.3 |
| 19.08.2015 | RAS | C | 7.1 | 6.75 | 27 | 1.7 |
| 19.08.2015 | RAS | C | 7.1 | 6.75 | 27 | 1.7 |
| 19.08.2015 | RAS | C | 7.1 | 6.75 | 27 | 1.7 |
| 19.08.2015 | RAS | C | 7.1 | 6.75 | 27 | 1.7 |
| 19.08.2015 | RAS | D | 6.7 | 7 | 28 | 1.8 |
| 19.08.2015 | RAS | D | 6.7 | 7 | 28 | 1.8 |
| 19.08.2015 | RAS | D | 6.7 | 7 | 28 | 1.8 |
| 19.08.2015 | RAS | D | 6.7 | 7 | 28 | 1.8 |
| 20.08.2015 | Hydro | D | 8.54 | 6.14 | 24.5 | 4.72 |
| 20.08.2015 | RAS | A | 6.41 | 7.16 | 27.6 | 1.232 |
| 20.08.2015 | RAS | A | 7.1 | 7.16 | 27.6 | 1.232 |
| 20.08.2015 | RAS | A | 6.58 | 7.16 | 27.6 | 1.232 |
| 20.08.2015 | RAS | A | 8.6 | 7.16 | 27.6 | 1.232 |
| 20.08.2015 | RAS | C | 6.07 | 7.02 | 27.1 | 1.8 |
| 20.08.2015 | RAS | C | 5.88 | 7.02 | 27.1 | 1.8 |
| 20.08.2015 | RAS | C | 6.55 | 7.02 | 27.1 | 1.8 |
| 20.08.2015 | RAS | C | 8.05 | 7.02 | 27.1 | 1.8 |
| 20.08.2015 | RAS | D | 6.06 | 7.12 | 27.9 | 1.8 |
| 20.08.2015 | RAS | D | 5.4 | 7.12 | 27.9 | 1.8 |
| 20.08.2015 | RAS | D | 5.7 | 7.12 | 27.9 | 1.8 |
| 20.08.2015 | RAS | D | 8.31 | 7.12 | 27.9 | 1.8 |
| 21.08.2015 | Hydro | D | 8.45 | 6.66 | 24.3 | 3.74 |
| 21.08.2015 | RAS | A | 6.5 | 7.27 | 27 | 1.239 |
| 21.08.2015 | RAS | A | 6.9 | 7.27 | 27 | 1.239 |
| 21.08.2015 | RAS | A | 6.7 | 7.27 | 27 | 1.239 |
| 21.08.2015 | RAS | A | 8.28 | 7.27 | 27 | 1.239 |
| 21.08.2015 | RAS | C | 6.15 | 7.05 | 27 | 1.8 |
| 21.08.2015 | RAS | C | 5.75 | 7.05 | 27 | 1.8 |
| 21.08.2015 | RAS | C | 6.7 | 7.05 | 27 | 1.8 |
| 21.08.2015 | RAS | C | 7.47 | 7.05 | 27 | 1.8 |
| 21.08.2015 | RAS | D | 6.28 | 7.15 | 27.5 | 1.8 |
| 21.08.2015 | RAS | D | 5.79 | 7.15 | 27.5 | 1.8 |
| 21.08.2015 | RAS | D | 5.9 | 7.15 | 27.5 | 1.8 |
| 21.08.2015 | RAS | D | 8.35 | 7.15 | 27.5 | 1.8 |
| 22.08.2015 | Hydro | D | 8.52 |  | 24.5 | 4.01 |
| 22.08.2015 | RAS | A | 4.9 | 7.33 | 27.2 | 1.26 |
| 22.08.2015 | RAS | A | 4.9 | 7.33 | 27.2 | 1.26 |
| 22.08.2015 | RAS | A | 4.9 | 7.33 | 27.2 | 1.26 |
| 22.08.2015 | RAS | A | 4.9 | 7.33 | 27.2 | 1.26 |
| 22.08.2015 | RAS | C | 7 | 7.13 | 27.3 | 1.8 |
| 22.08.2015 | RAS | C | 7 | 7.13 | 27.3 | 1.8 |
| 22.08.2015 | RAS | C | 7 | 7.13 | 27.3 | 1.8 |
| 22.08.2015 | RAS | C | 7 | 7.13 | 27.3 | 1.8 |
| 22.08.2015 | RAS | D | 6.8 | 7.21 | 27.6 | 1.8 |
| 22.08.2015 | RAS | D | 6.8 | 7.21 | 27.6 | 1.8 |
| 22.08.2015 | RAS | D | 6.8 | 7.21 | 27.6 | 1.8 |
| 22.08.2015 | RAS | D | 6.8 | 7.21 | 27.6 | 1.8 |
| 23.08.2015 | Hydro | D | 8.56 | 6.85 | 24.5 | 4.36 |
| 23.08.2015 | RAS | A | 5 | 7.29 | 27.5 | 1.472 |
| 23.08.2015 | RAS | A | 5 | 7.29 | 27.5 | 1.472 |
| 23.08.2015 | RAS | A | 5 | 7.29 | 27.5 | 1.472 |
| 23.08.2015 | RAS | A | 5 | 7.29 | 27.5 | 1.472 |
| 23.08.2015 | RAS | C | 7 | 7.15 | 27.4 | 1.971 |
| 23.08.2015 | RAS | C | 7 | 7.15 | 27.4 | 1.971 |
| 23.08.2015 | RAS | C | 7 | 7.15 | 27.4 | 1.971 |
| 23.08.2015 | RAS | C | 7 | 7.15 | 27.4 | 1.971 |
| 23.08.2015 | RAS | D | 6.7 | 7.22 | 27.7 | 1.831 |
| 23.08.2015 | RAS | D | 6.7 | 7.22 | 27.7 | 1.831 |
| 23.08.2015 | RAS | D | 6.7 | 7.22 | 27.7 | 1.831 |
| 23.08.2015 | RAS | D | 6.7 | 7.22 | 27.7 | 1.831 |
| 24.08.2015 | Hydro | D | 8.31 | 6.73 | 25.2 | 4.65 |
| 24.08.2015 | RAS | A | 6.1 | 7.12 | 27.6 | 1.302 |
| 24.08.2015 | RAS | A | 6.3 | 7.12 | 27.6 | 1.302 |
| 24.08.2015 | RAS | A | 6.48 | 7.12 | 27.6 | 1.302 |
| 24.08.2015 | RAS | A | 7.96 | 7.12 | 27.6 | 1.302 |
| 24.08.2015 | RAS | C | 5.76 | 6.97 | 27.3 | 1.9 |
| 24.08.2015 | RAS | C | 5.31 | 6.97 | 27.3 | 1.9 |
| 24.08.2015 | RAS | C | 7.1 | 6.97 | 27.3 | 1.9 |
| 24.08.2015 | RAS | C | 7.84 | 6.97 | 27.3 | 1.9 |
| 24.08.2015 | RAS | D | 5.99 | 7.11 | 27.8 | 1.9 |
| 24.08.2015 | RAS | D | 5.61 | 7.11 | 27.8 | 1.9 |
| 24.08.2015 | RAS | D | 6.1 | 7.11 | 27.8 | 1.9 |
| 24.08.2015 | RAS | D | 8.13 | 7.11 | 27.8 | 1.9 |
| 25.08.2015 | Hydro | D | 8.39 | 6.67 | 25 | 4.97 |
| 25.08.2015 | RAS | A | 6.12 | 7.35 | 27.1 | 1.281 |
| 25.08.2015 | RAS | A | 6.3 | 7.35 | 27.1 | 1.281 |
| 25.08.2015 | RAS | A | 6.52 | 7.35 | 27.1 | 1.281 |
| 25.08.2015 | RAS | A | 8.09 | 7.35 | 27.1 | 1.281 |
| 25.08.2015 | RAS | C | 5.84 | 7.16 | 27 | 1.8 |
| 25.08.2015 | RAS | C | 5.44 | 7.16 | 27 | 1.8 |
| 25.08.2015 | RAS | C | 6 | 7.16 | 27 | 1.8 |
| 25.08.2015 | RAS | C | 7.95 | 7.16 | 27 | 1.8 |
| 25.08.2015 | RAS | D | 5.64 | 7.28 | 27.5 | 1.8 |
| 25.08.2015 | RAS | D | 5.67 | 7.28 | 27.5 | 1.8 |
| 25.08.2015 | RAS | D | 6.1 | 7.28 | 27.5 | 1.8 |
| 25.08.2015 | RAS | D | 8.26 | 7.28 | 27.5 | 1.8 |
| 26.08.2015 | Hydro | D | 8.6 | 6.72 | 24.4 | 5.23 |
| 26.08.2015 | RAS | A | 6.04 | 7.39 | 27.2 | 1.271 |
| 26.08.2015 | RAS | A | 6.6 | 7.39 | 27.2 | 1.271 |
| 26.08.2015 | RAS | A | 6.14 | 7.39 | 27.2 | 1.271 |
| 26.08.2015 | RAS | A | 8.17 | 7.39 | 27.2 | 1.271 |
| 26.08.2015 | RAS | C | 5.85 | 7.25 | 26.8 | 1.9 |
| 26.08.2015 | RAS | C | 5.46 | 7.25 | 26.8 | 1.9 |
| 26.08.2015 | RAS | C | 6.2 | 7.25 | 26.8 | 1.9 |
| 26.08.2015 | RAS | C | 8.07 | 7.25 | 26.8 | 1.9 |
| 26.08.2015 | RAS | D | 5.78 | 7.27 | 27.5 | 1.9 |
| 26.08.2015 | RAS | D | 5.28 | 7.27 | 27.5 | 1.9 |
| 26.08.2015 | RAS | D | 5.4 | 7.27 | 27.5 | 1.9 |
| 26.08.2015 | RAS | D | 8.32 | 7.27 | 27.5 | 1.9 |
| 27.08.2015 | Hydro | D | 8.13 | 5.78 | 27 | 1.809 |
| 27.08.2015 | RAS | A | 6.31 | 7.36 | 27.2 | 1.265 |
| 27.08.2015 | RAS | A | 6.4 | 7.36 | 27.2 | 1.265 |
| 27.08.2015 | RAS | A | 6.61 | 7.36 | 27.2 | 1.265 |
| 27.08.2015 | RAS | A | 8.12 | 7.36 | 27.2 | 1.265 |
| 27.08.2015 | RAS | C | 5.8 | 7.15 | 27 | 1.9 |
| 27.08.2015 | RAS | C | 5.75 | 7.15 | 27 | 1.9 |
| 27.08.2015 | RAS | C | 6 | 7.15 | 27 | 1.9 |
| 27.08.2015 | RAS | C | 7.97 | 7.15 | 27 | 1.9 |
| 27.08.2015 | RAS | D | 6.02 | 7.27 | 27.6 | 1.9 |
| 27.08.2015 | RAS | D | 5.85 | 7.27 | 27.6 | 1.9 |
| 27.08.2015 | RAS | D | 5.7 | 7.27 | 27.6 | 1.9 |
| 27.08.2015 | RAS | D | 8.25 | 7.27 | 27.6 | 1.9 |
| 28.08.2015 | Hydro | D | 8.31 | 6.87 | 25.4 | 2.38 |
| 28.08.2015 | RAS | A | 6.03 | 7.32 | 27.6 | 1.27 |
| 28.08.2015 | RAS | A | 6.3 | 7.32 | 27.6 | 1.27 |
| 28.08.2015 | RAS | A | 5.8 | 7.32 | 27.6 | 1.27 |
| 28.08.2015 | RAS | A | 8.05 | 7.32 | 27.6 | 1.27 |
| 28.08.2015 | RAS | C | 5.86 | 7.17 | 27.4 | 1.9 |
| 28.08.2015 | RAS | C | 6.1 | 7.17 | 27.4 | 1.9 |
| 28.08.2015 | RAS | C | 7.7 | 7.17 | 27.4 | 1.9 |
| 28.08.2015 | RAS | C | 6.8 | 7.17 | 27.4 | 1.9 |
| 28.08.2015 | RAS | D | 6.9 | 7.27 | 28 | 1.9 |
| 28.08.2015 | RAS | D | 5.26 | 7.27 | 28 | 1.9 |
| 28.08.2015 | RAS | D | 5.4 | 7.27 | 28 | 1.9 |
| 28.08.2015 | RAS | D | 8.18 | 7.27 | 28 | 1.9 |
| 29.08.2015 | Hydro | D | 8.6 | 6.52 | 24.3 |  |
| 29.08.2015 | RAS | A | 6.8 | 7.35 | 27 | 1.3 |
| 29.08.2015 | RAS | A | 6.8 | 7.35 | 27 | 1.3 |
| 29.08.2015 | RAS | A | 6.8 | 7.35 | 27 | 1.3 |
| 29.08.2015 | RAS | A | 6.8 | 7.35 | 27 | 1.3 |
| 29.08.2015 | RAS | C | 7.5 | 7.22 | 26.9 | 1.8 |
| 29.08.2015 | RAS | C | 7.5 | 7.22 | 26.9 | 1.8 |
| 29.08.2015 | RAS | C | 7.5 | 7.22 | 26.9 | 1.8 |
| 29.08.2015 | RAS | C | 7.5 | 7.22 | 26.9 | 1.8 |
| 29.08.2015 | RAS | D | 6.7 | 7.21 | 27.5 | 1.9 |
| 29.08.2015 | RAS | D | 6.7 | 7.21 | 27.5 | 1.9 |
| 29.08.2015 | RAS | D | 6.7 | 7.21 | 27.5 | 1.9 |
| 29.08.2015 | RAS | D | 6.7 | 7.21 | 27.5 | 1.9 |
| 30.08.2015 | Hydro | D | 8.75 | 6.52 | 24.4 |  |
| 30.08.2015 | RAS | A | 6.9 | 7.31 | 27.1 |  |
| 30.08.2015 | RAS | A | 6.9 | 7.31 | 27.1 |  |
| 30.08.2015 | RAS | A | 6.9 | 7.31 | 27.1 |  |
| 30.08.2015 | RAS | A | 6.9 | 7.31 | 27.1 |  |
| 30.08.2015 | RAS | C | 7.4 | 7.23 | 27.2 | 1.8 |
| 30.08.2015 | RAS | C | 7.4 | 7.23 | 27.2 | 1.8 |
| 30.08.2015 | RAS | C | 7.4 | 7.23 | 27.2 | 1.8 |
| 30.08.2015 | RAS | C | 7.4 | 7.23 | 27.2 | 1.8 |
| 30.08.2015 | RAS | D | 6.8 | 7.15 | 27.5 | 1.9 |
| 30.08.2015 | RAS | D | 6.8 | 7.15 | 27.5 | 1.9 |
| 30.08.2015 | RAS | D | 6.8 | 7.15 | 27.5 | 1.9 |
| 30.08.2015 | RAS | D | 6.8 | 7.15 | 27.5 | 1.9 |
| 31.08.2015 | Hydro | D | 7.85 | 7.04 | 28.8 | 2.9 |
| 31.08.2015 | RAS | A | 5.7 | 7.3 | 29.3 | 1.3 |
| 31.08.2015 | RAS | A | 5.7 | 7.3 | 29.3 | 1.3 |
| 31.08.2015 | RAS | A | 5.7 | 7.3 | 29.3 | 1.3 |
| 31.08.2015 | RAS | A | 5.7 | 7.3 | 29.3 | 1.3 |
| 31.08.2015 | RAS | C | 6.8 | 7.05 | 29.5 | 2 |
| 31.08.2015 | RAS | C | 6.8 | 7.05 | 29.5 | 2 |
| 31.08.2015 | RAS | C | 6.8 | 7.05 | 29.5 | 2 |
| 31.08.2015 | RAS | C | 6.8 | 7.05 | 29.5 | 2 |
| 31.08.2015 | RAS | D | 6.6 | 6.89 | 29.6 | 2 |
| 31.08.2015 | RAS | D | 6.6 | 6.89 | 29.6 | 2 |
| 31.08.2015 | RAS | D | 6.6 | 6.89 | 29.6 | 2 |
| 31.08.2015 | RAS | D | 6.6 | 6.89 | 29.6 | 2 |
| 01.09.2015 | Hydro | D | 7.91 | 6.48 | 27.7 | 3.15 |
| 01.09.2015 | RAS | A | 6.3 |  | 29.3 | 1.4 |
| 01.09.2015 | RAS | A | 6.3 |  | 29.3 | 1.4 |
| 01.09.2015 | RAS | A | 6.3 |  | 29.3 | 1.4 |
| 01.09.2015 | RAS | A | 6.3 |  | 29.3 | 1.4 |
| 01.09.2015 | RAS | C | 6.6 |  | 29.5 | 2.1 |
| 01.09.2015 | RAS | C | 6.6 |  | 29.5 | 2.1 |
| 01.09.2015 | RAS | C | 6.6 |  | 29.5 | 2.1 |
| 01.09.2015 | RAS | C | 6.6 |  | 29.5 | 2.1 |
| 01.09.2015 | RAS | D | 6.5 |  | 29.6 | 2.1 |
| 01.09.2015 | RAS | D | 6.5 |  | 29.6 | 2.1 |
| 01.09.2015 | RAS | D | 6.5 |  | 29.6 | 2.1 |
| 01.09.2015 | RAS | D | 6.5 |  | 29.6 | 2.1 |
| 02.09.2015 | Hydro | D | 8.55 | 6.6 | 25.1 | 3.23 |
| 02.09.2015 | RAS | A | 5.82 | 7.34 | 28.1 | 1.3 |
| 02.09.2015 | RAS | A | 6.21 | 7.34 | 28.1 | 1.3 |
| 02.09.2015 | RAS | A | 6.42 | 7.34 | 28.1 | 1.3 |
| 02.09.2015 | RAS | A |  | 7.34 | 28.1 | 1.3 |
| 02.09.2015 | RAS | C | 5.45 | 7.34 | 27.5 | 2 |
| 02.09.2015 | RAS | C | 5.15 | 7.34 | 27.5 | 2 |
| 02.09.2015 | RAS | C | 5.33 | 7.34 | 27.5 | 2 |
| 02.09.2015 | RAS | C |  | 7.34 | 27.5 | 2 |
| 02.09.2015 | RAS | D | 5.91 | 7.32 | 28.1 | 2 |
| 02.09.2015 | RAS | D | 5.5 | 7.32 | 28.1 | 2 |
| 02.09.2015 | RAS | D | 5.18 | 7.32 | 28.1 | 2 |
| 02.09.2015 | RAS | D |  | 7.32 | 28.1 | 2 |
| 03.09.2015 | Hydro | D | 8.49 | 6.22 | 24.3 | 3.21 |
| 03.09.2015 | RAS | A | 6.75 | 7.77 | 24 | 1.1 |
| 03.09.2015 | RAS | A | 7.07 | 7.77 | 24 | 1.1 |
| 03.09.2015 | RAS | A | 7.15 | 7.77 | 24 | 1.1 |
| 03.09.2015 | RAS | A |  | 7.77 | 24 | 1.1 |
| 03.09.2015 | RAS | C | 5.79 | 7.1 | 26.9 | 2 |
| 03.09.2015 | RAS | C | 5.23 | 7.1 | 26.9 | 2 |
| 03.09.2015 | RAS | C | 5.63 | 7.1 | 26.9 | 2 |
| 03.09.2015 | RAS | C |  | 7.1 | 26.9 | 2 |
| 03.09.2015 | RAS | D | 5.17 | 7.1 | 26.9 | 2 |
| 03.09.2015 | RAS | D | 5.13 | 7.1 | 26.9 | 2 |
| 03.09.2015 | RAS | D | 5.76 | 7.1 | 26.9 | 2 |
| 03.09.2015 | RAS | D |  | 7.1 | 26.9 | 2 |
| 04.09.2015 | Hydro | D | 8.65 | 6.17 |  | 3.62 |
| 04.09.2015 | RAS | A | 5.18 | 7.69 | 26.3 | 1.1 |
| 04.09.2015 | RAS | A | 5.51 | 7.69 | 26.3 | 1.1 |
| 04.09.2015 | RAS | A | 5.3 | 7.69 | 26.3 | 1.1 |
| 04.09.2015 | RAS | A |  | 7.69 | 26.3 | 1.1 |
| 04.09.2015 | RAS | C | 5.1 | 7.13 | 26.4 | 2.1 |
| 04.09.2015 | RAS | C | 4.3 | 7.13 | 26.4 | 2.1 |
| 04.09.2015 | RAS | C | 4.75 | 7.13 | 26.4 | 2.1 |
| 04.09.2015 | RAS | C |  | 7.13 | 26.4 | 2.1 |
| 04.09.2015 | RAS | D | 4.98 | 7.18 | 27.1 | 2 |
| 04.09.2015 | RAS | D | 3.97 | 7.18 | 27.1 | 2 |
| 04.09.2015 | RAS | D | 3.98 | 7.18 | 27.1 | 2 |
| 04.09.2015 | RAS | D |  | 7.18 | 27.1 | 2 |
| 05.09.2015 | Hydro | D | 8.58 | 6.61 | 23.7 | 3.08 |
| 05.09.2015 | RAS | A | 6.7 | 7.63 | 26.3 | 1.1 |
| 05.09.2015 | RAS | A | 6.7 | 7.63 | 26.3 | 1.1 |
| 05.09.2015 | RAS | A | 6.7 | 7.63 | 26.3 | 1.1 |
| 05.09.2015 | RAS | A | 6.7 | 7.63 | 26.3 | 1.1 |
| 05.09.2015 | RAS | C | 7.3 | 7.07 | 26.1 | 2.1 |
| 05.09.2015 | RAS | C | 7.3 | 7.07 | 26.1 | 2.1 |
| 05.09.2015 | RAS | C | 7.3 | 7.07 | 26.1 | 2.1 |
| 05.09.2015 | RAS | C | 7.3 | 7.07 | 26.1 | 2.1 |
| 05.09.2015 | RAS | D | 7.1 | 7.19 | 26.8 | 2.1 |
| 05.09.2015 | RAS | D | 7.1 | 7.19 | 26.8 | 2.1 |
| 05.09.2015 | RAS | D | 7.1 | 7.19 | 26.8 | 2.1 |
| 05.09.2015 | RAS | D | 7.1 | 7.19 | 26.8 | 2.1 |
| 06.09.2015 | Hydro | D | 8.65 |  | 23.2 | 3.22 |
| 06.09.2015 | RAS | A | 6.7 | 7.51 | 26 | 1.1 |
| 06.09.2015 | RAS | A | 6.7 | 7.51 | 26 | 1.1 |
| 06.09.2015 | RAS | A | 6.7 | 7.51 | 26 | 1.1 |
| 06.09.2015 | RAS | A | 6.7 | 7.51 | 26 | 1.1 |
| 06.09.2015 | RAS | C | 7.3 | 6.93 | 26 | 2.2 |
| 06.09.2015 | RAS | C | 7.3 | 6.93 | 26 | 2.2 |
| 06.09.2015 | RAS | C | 7.3 | 6.93 | 26 | 2.2 |
| 06.09.2015 | RAS | C | 7.3 | 6.93 | 26 | 2.2 |
| 06.09.2015 | RAS | D | 7.2 | 7.11 | 26.5 | 2.1 |
| 06.09.2015 | RAS | D | 7.2 | 7.11 | 26.5 | 2.1 |
| 06.09.2015 | RAS | D | 7.2 | 7.11 | 26.5 | 2.1 |
| 06.09.2015 | RAS | D | 7.2 | 7.11 | 26.5 | 2.1 |
| 07.09.2015 | Hydro | D | 8.75 | 6.28 | 23.4 | 3.35 |
| 07.09.2015 | RAS | A | 6.13 | 7.44 | 26 | 1.1 |
| 07.09.2015 | RAS | A | 6.68 | 7.44 | 26 | 1.1 |
| 07.09.2015 | RAS | A | 6.46 | 7.44 | 26 | 1.1 |
| 07.09.2015 | RAS | A |  | 7.44 | 26 | 1.1 |
| 07.09.2015 | RAS | C | 5.78 | 6.94 | 25.8 | 2.2 |
| 07.09.2015 | RAS | C | 5.78 | 6.94 | 25.8 | 2.2 |
| 07.09.2015 | RAS | C | 5.78 | 6.94 | 25.8 | 2.2 |
| 07.09.2015 | RAS | C | 5.78 | 6.94 | 25.8 | 2.2 |
| 07.09.2015 | RAS | D | 5.92 | 7.1 | 26.5 | 2.1 |
| 07.09.2015 | RAS | D | 5.6 | 7.1 | 26.5 | 2.1 |
| 07.09.2015 | RAS | D | 5.45 | 7.1 | 26.5 | 2.1 |
| 07.09.2015 | RAS | D |  | 7.1 | 26.5 | 2.1 |
