# Supplementary material for: Decoupled systems on trial: Eliminating bottlenecks to improve aquaponic processes
Source: PLoS One. 2017 Sep 28;12(9):e0183056. doi: 10.1371/journal.pone.0183056 (PMC5619720; doi:10.1371/journal.pone.0183056)
Supplement: S2 Table — Nutrients in RAS C correspond to the nutrients in Hydro C, since both are arranged as coupled aquaponic system. (DOCX) [file pone.0183056.s002.docx]

**S2 Table: Dissolved nutrients in the fish (RAS A, C, D) and hydroponic units (Hydro C, Hydro D) of a conventional aquaculture reference (A), a coupled (C) and a decoupled (D) aquaponic system, assessed over the experimental period of 154 days (07.04 - 07.09.2015). Nutrients in RAS C correspond to the nutrients in Hydro C, since both are arranged as coupled aquaponic system.**

| **RAS / Hydro** | **sampling date** | **NO_3_^-^-N**  **[mgL^-1^]** | **NO_2_^-^-N**  **[mgL^-1^]** | **NH_4_^+^-N**  **[mgL^-1^]** | **PO_4_^3-^**  **[mgL^-1^]** | **K^+^**  **[mgL^-1^]** | **Ca^2+^**  **[mgL^-1^]** | **Mg^2+^**  **[mgL^-1^]** | **SO_4_^2-^**  **[mgL^-1^]** | **Cl^-^**  **[mgL^-1^]** | **Fe^2+^**  **[mgL^-1^]** |
| --- | --- | --- | --- | --- | --- | --- | --- | --- | --- | --- | --- |
| RAS A | 24.04.2015 | 12.3 | 0.143 | 0.16 | 14.14 | 21 | 122.8 | 14.1 | 140 | 41.5 | 0.01 |
| RAS A | 30.04.2015 | 19 | 0.036 | 0.08 | 15.38 | 23 | 124 | 14.1 | 190 | 31.5 | 0.01 |
| RAS A | 08.05.2015 | 25.8 | 0.117 | 0.14 | 13.16 | 19 | 127.2 | 14.1 | 180 | 26 | 0.01 |
| RAS A | 21.05.2015 | 26.9 | 0.061 | 0.09 | 11.3 | 22.5 | 124 | 14.6 | 180 | 37 | 0.01 |
| RAS A | 28.05.2015 | 24.8 | 0.052 | 0.06 | 8.66 | 22 | 134.4 | 27.4 | 175 | 28 | 0.01 |
| RAS A | 04.06.2015 | 32.8 | 0.057 | 0.07 | 8.49 | 23 | 136.8 | 27.9 | 180 |  | 0.02 |
| RAS A | 10.06.2015 | 20 | 0.034 | 0.05 | 5.28 | 13 | 128 | 17.5 | 160 | 29.5 | 0.01 |
| RAS A | 18.06.2015 | 23.5 | 0.056 | 0.4 | 6.47 | 18 | 142 | 14.8 | 160 | 27 | 0 |
| RAS A | 26.06.2015 | 34 | 0.068 | 0.07 | 7.07 | 23 | 132.8 | 16.7 | 155 | 27.5 | 0.01 |
| RAS A | 02.07.2015 | 44 | 0.07 | 0.11 | 7.79 | 23 | 133.6 | 21.8 | 170 | 32 | 0.01 |
| RAS A | 10.07.2015 | 46 | 0.032 | 0.07 | 7.55 |  | 133.2 | 17.2 | 170 | 33 | 0.01 |
| RAS A | 16.07.2015 | 54 | 0.044 | 0.07 | 6.07 | 17 | 135.2 | 15.5 | 165 | 29 | 0.01 |
| RAS A | 22.07.2015 | 51.5 | 0.042 | 0.07 | 6.47 | 18 | 144.8 | 15.5 | 170 | 31 | 0.01 |
| RAS A | 30.07.2015 | 57.5 | 0.052 | 0.1 | 5.34 | 21 | 134 | 16.7 | 170 | 29.5 | 0 |
| RAS A | 07.08.2015 | 58.5 | 0.031 | 0.04 | 5.54 | 16 | 128 | 14.8 | 160 | 33 | 0.02 |
| RAS A | 11.08.2015 | 68 | 0.04 | 0.07 | 6.15 | 16 | 136.8 | 15.8 | 165 | 37 | 0.01 |
| RAS A | 20.08.2015 | 64 | 0.047 | 0.13 | 4.41 | 19 | 147.6 | 16.7 |  |  |  |
| RAS A | 10.09.2015 | 73 | 0.031 | 0.04 | 6.25 | 17 | 152 | 17.5 | 155 | 44.5 | 0.01 |
| RAS C | 24.04.2015 | 15.4 | 0.049 | 0.05 | 16.74 | 25 | 119.2 | 13.3 | 150 | 45 | 0.01 |
| RAS C | 30.04.2015 | 24.1 | 0.056 | 0.07 | 17.36 | 30.5 | 120.4 | 14.3 | 200 | 34.5 | 0 |
| RAS C | 08.05.2015 | 19.9 | 0.124 | 0.04 | 15.48 | 25.5 | 122 | 14.1 | 200 | 25.5 | 0 |
| RAS C | 21.05.2015 | 27.3 | 0.067 | 0.05 | 13.06 | 26.5 | 139.2 | 16.7 | 200 | 37.5 | 0.01 |
| RAS C | 28.05.2015 | 48.8 | 0.071 | 0.03 | 11.29 | 28.5 | 148 | 27.2 | 195 | 30 | 0 |
| RAS C | 04.06.2015 | 48.8 | 0.073 | 0.05 | 11.2 | 31.5 | 144.8 | 29.6 | 195 |  | 0.01 |
| RAS C | 10.06.2015 | 45.5 | 0.043 | 0.04 | 9.22 | 27 | 142 | 16.7 | 190 | 32.5 | 0 |
| RAS C | 18.06.2015 | 51 | 0.072 | 0.3 | 9.43 | 38 | 146.4 | 20.4 | 195 | 33.5 | 0.01 |
| RAS C | 26.06.2015 | 65 | 0.084 | 0.08 | 9.68 | 48 | 153.6 | 19.9 | 180 | 32.5 | 0.01 |
| RAS C | 02.07.2015 | 76.5 | 0.093 | 0.18 | 10.85 | 50 | 152 | 20.6 | 200 | 40 | 0.01 |
| RAS C | 10.07.2015 | 82 | 0.039 | 0.04 | 9.5 |  | 154 | 18.2 | 200 | 39 | 0.02 |
| RAS C | 16.07.2015 | 60.5 | 0.051 | 0.07 | 9.57 | 40 | 142.8 | 19.9 | 195 | 39 | 0.03 |
| RAS C | 22.07.2015 | 56 | 0.056 | 0.05 | 8.03 | 38 | 136.4 | 17.5 | 185 | 36.5 | 0.03 |
| RAS C | 30.07.2015 | 62.5 | 0.063 | 0.06 | 6.18 | 38 | 146 | 21.6 | 200 | 42 | 0 |
| RAS C | 07.08.2015 | 60.5 | 0.054 | 0.05 | 6.82 | 24 | 147.6 | 19.2 | 210 | 39.5 | 0.04 |
| RAS C | 11.08.2015 | 55 | 0.045 | 0.07 | 7.24 | 27 | 151.6 | 19.4 | 175 | 45.5 | 0.02 |
| RAS C | 20.08.2015 | 76 | 0.079 | 0.07 | 5.76 | 33 | 151.6 | 21.6 |  |  |  |
| RAS C | 10.09.2015 | 99.5 | 0.037 | 0.03 | 5.43 | 25 | 146 | 21.6 | 185 | 54.5 | 0 |
| RAS D | 24.04.2015 | 12.3 | 0.021 | 0.03 | 19.62 | 24.5 | 126 | 15.3 | 150 | 45.5 | 0.01 |
| RAS D | 30.04.2015 | 22.7 | 0.011 | 0.02 | 14.24 | 19.5 | 124.4 | 14.6 | 165 | 30.5 | 0.01 |
| RAS D | 08.05.2015 | 21.3 | 0.06 | 0.03 | 13.3 | 22 | 128.4 | 14.6 | 165 | 23 | 0.01 |
| RAS D | 21.05.2015 | 22.1 | 0.075 | 0.07 | 15.02 | 25 | 147.2 | 17.2 | 175 | 36 | 0.01 |
| RAS D | 28.05.2015 | 28.6 | 0.044 | 0.04 | 13.68 | 25.5 | 146 | 28.9 | 175 | 30 | 0.01 |
| RAS D | 04.06.2015 | 36.4 | 0.044 | 0.05 | 14.94 | 29 | 139.2 | 30.8 | 180 |  | 0.01 |
| RAS D | 10.06.2015 | 40.5 | 0.028 | 0.01 | 12.52 | 27 | 156.4 | 19.2 | 180 | 33.5 | 0 |
| RAS D | 18.06.2015 | 44 | 0.06 | 0.4 | 13.16 | 37 | 153.6 | 20.6 | 185 | 32 | 0.01 |
| RAS D | 26.06.2015 | 57 | 0.08 | 0.02 | 13.51 | 47 | 152 | 20.6 | 180 | 31.5 | 0.02 |
| RAS D | 02.07.2015 | 60 | 0.076 | 0.06 | 14.59 | 49 | 146 | 18.2 | 200 | 38 | 0.01 |
| RAS D | 10.07.2015 | 69 | 0.029 | 0.05 | 13.05 |  | 152 | 22.1 | 195 | 38 | 0.02 |
| RAS D | 16.07.2015 | 81 | 0.055 | 0.06 | 12.47 | 42 | 148.4 | 34.0 | 185 | 37 | 0.01 |
| RAS D | 22.07.2015 | 76.5 | 0.051 | 0.05 | 11.49 | 42 | 149.6 | 19.7 | 190 | 40 | 0.01 |
| RAS D | 30.07.2015 | 84 | 0.069 | 0.07 | 10.54 | 41 | 146 | 17.7 | 205 | 39 | 0.01 |
| RAS D | 07.08.2015 | 92 | 0.041 | 0.04 | 9.22 | 28 | 141.2 | 19.2 | 180 | 35.5 | 0.02 |
| RAS D | 11.08.2015 | 94.5 | 0.044 | 0.05 | 9.73 | 28 | 148.4 | 19.2 | 175 | 24 | 0.01 |
| RAS D | 20.08.2015 | 100.5 | 0.08 | 0.08 | 8.17 | 34 | 151.2 | 21.1 |  |  |  |
| RAS D | 10.09.2015 | 116 | 0.039 | 0.03 | 11.16 | 29 | 156.4 | 19.4 | 195 | 41.5 | 0.01 |
| Hydro D | 24.04.2015 | 82 | 0.13 | 3.8 | 33.9 | 205 | 620.0 | 57.0 | 260 | 53 | 0.01 |
| Hydro D | 30.04.2015 | 115.5 | 0.012 | 3.4 | 24.35 | 210 | 492.0 | 42.0 | 330 | 39 | 0.01 |
| Hydro D | 08.05.2015 | 107.5 | 0.011 | 2.6 | 27.65 | 85 | 396.0 | 33.5 | 400 | 26 | 0.31 |
| Hydro D | 21.05.2015 | 224 | 0.012 | 0 | 13.65 | 30 | 404.0 | 50.2 | 860 | 43.5 | 0.1 |
| Hydro D | 28.05.2015 | 100 | 0.006 | 0 | 31.4 | 14 | 380.0 | 35.2 | 540 | 40 | 0.02 |
| Hydro D | 04.06.2015 | 116 | 0.051 | 6.4 | 31.75 | 38 | 271.2 | 26.7 | 260 |  | 0.02 |
| Hydro D | 10.06.2015 | 136 | 0.003 | 0.04 | 10.72 | 16 | 440.0 | 40.0 | 720 | 71.5 | 0.02 |
| Hydro D | 18.06.2015 | 228 | 0.011 | 0.1 | 14.74 | 14 | 662.0 | 57.0 | 1300 | 139 | 0.04 |
| Hydro D | 26.06.2015 | 162 | 0.014 | 1 | 14.14 | 74 | 692.0 | 95.9 | 320 | 42 | 0.04 |
| Hydro D | 02.07.2015 | 174 | 0.028 | 1.4 | 12 | 96 | 440.0 | 36.4 | 300 | 54 | 0.08 |
| Hydro D | 10.07.2015 | 292 | 0.013 | 0.13 | 10.78 |  | 476.0 | 43.7 | 740 | 58 | 0.22 |
| Hydro D | 16.07.2015 | 156 | 0.006 | 0.07 | 7.18 | 56 | 412.0 | 88.6 | 280 | 55 | 0.04 |
| Hydro D | 22.07.2015 | 238 | 0.008 | 0.09 | 7.18 | 9 | 484.0 | 42.0 | 460 | 86.5 | 0.12 |
| Hydro D | 30.07.2015 | 144 | 0.014 | 0.02 | 3.7 | 7 | 399.2 | 51.0 | 400 | 77 | 0.08 |
| Hydro D | 07.08.2015 | 168 | 0.063 | 0.02 | 8.22 | 3.5 | 436.0 | 36.4 | 320 | 65 | 0.13 |
| Hydro D | 11.08.2015 | 142 | 0.017 | 0.01 | 8.2 | 10.5 | 440.0 | 40.0 | 380 | 91.5 | 0.05 |
| Hydro D | 20.08.2015 | 230 | 0.013 | 0.03 | 5.18 | 0.7 | 696.0 | 74.0 |  |  |  |
| Hydro D | 10.09.2015 | 158 | 0.01 | 0 | 5.34 | 2 | 356.0 | 49.8 | 420 | 112.5 | 0.12 |
