# Supplementary material for: Decoupled systems on trial: Eliminating bottlenecks to improve aquaponic processes
Source: PLoS One. 2017 Sep 28;12(9):e0183056. doi: 10.1371/journal.pone.0183056 (PMC5619720; doi:10.1371/journal.pone.0183056)
Supplement: S3 Table — Additionally data for fish and sludge are presented. (DOCX) [file pone.0183056.s003.docx]

S3 Table: Elemental analysis (ICP-OES) of plant leaves and tomatoes harvested from the hydroponic unit of the coupled (Hydro C) and the decoupled (Hydro D) aquaponic system after 30 d, 63 d, 94 d, 122 d and 154 d. Additionally data for fish and sludge are presented.

| **system** | **date** | **sample** | **Ca**  **[g kg^-1^]** | **K**  **[g kg^-1^]** | **Mg**  **[g kg^-1^]** | **Na**  **[g kg^-1^]** | **P**  **[g kg^-1^]** |
| --- | --- | --- | --- | --- | --- | --- | --- |
| Hydro C | 28.05.2015 | leaf | 32.51 | 44.58 | 4.64 | 0.32 | 4.86 |
| Hydro C | 28.05.2015 | leaf | 29.68 | 44.74 | 4.21 | 0.29 | 5.20 |
| Hydro C | 28.05.2015 | leaf | 28.94 | 46.98 | 4.30 | 0.33 | 5.13 |
| Hydro C | 02.07.2015 | leaf | 31.69 | 38.51 | 4.37 | 0.33 | 4.14 |
| Hydro C | 02.07.2015 | leaf | 35.69 | 34.12 | 5.33 | 0.27 | 4.47 |
| Hydro C | 02.07.2015 | leaf | 29.77 | 48.29 | 4.71 | 0.33 | 4.68 |
| Hydro C | 12.08.2015 | leaf | 23.50 | 37.11 | 3.76 | 0.34 | 4.96 |
| Hydro C | 12.08.2015 | leaf | 28.13 | 32.88 | 4.19 | 0.31 | 4.80 |
| Hydro C | 12.08.2015 | leaf | 26.48 | 35.88 | 3.89 | 0.35 | 4.42 |
| Hydro C | 09.09.2015 | leaf | 36.34 | 31.15 | 3.50 | 0.37 | 4.03 |
| Hydro C | 09.09.2015 | leaf | 35.87 | 31.51 | 4.18 | 0.40 | 4.04 |
| Hydro C | 09.09.2015 | leaf | 29.90 | 36.83 | 3.59 | 0.33 | 4.92 |
| Hydro C | 28.05.2015 | tomato | 1.77 | 47.23 | 1.48 | 0.27 | 4.70 |
| Hydro C | 28.05.2015 | tomato | 3.32 | 47.69 | 1.26 | 0.28 | 4.33 |
| Hydro C | 28.05.2015 | tomato | 1.53 | 47.52 | 1.21 | 0.26 | 4.64 |
| Hydro C | 02.07.2015 | tomato | 1.95 | 43.59 | 1.29 | 0.24 | 4.20 |
| Hydro C | 02.07.2015 | tomato | 2.45 | 38.73 | 1.27 | 0.20 | 4.19 |
| Hydro C | 02.07.2015 | tomato | 2.01 | 42.37 | 1.53 | 0.24 | 4.50 |
| Hydro C | 12.08.2015 | tomato | 1.01 | 42.79 | 1.46 | 0.34 | 3.43 |
| Hydro C | 12.08.2015 | tomato | 1.61 | 39.60 | 1.53 | 0.27 | 4.35 |
| Hydro C | 12.08.2015 | tomato | 1.22 | 40.59 | 1.51 | 0.27 | 4.33 |
| Hydro C | 09.01.2015 | tomato | 1.17 | 38.22 | 1.21 | 0.20 | 4.14 |
| Hydro C | 09.09.2015 | tomato | 1.07 | 40.81 | 1.38 | 0.23 | 4.59 |
| Hydro C | 09.09.2015 | tomato | 0.99 | 46.86 | 1.86 | 0.49 | 4.57 |
| Hydro D | 28.05.2015 | leaf | 25.78 | 41.92 | 3.96 | 1.07 | 2.78 |
| Hydro D | 28.05.2015 | leaf | 22.91 | 37.26 | 4.03 | 0.91 | 2.62 |
| Hydro D | 28.05.2015 | leaf | 31.45 | 40.46 | 3.69 | 1.18 | 2.76 |
| Hydro D | 02.07.2015 | leaf | 21.13 | 47.01 | 3.15 | 1.33 | 2.14 |
| Hydro D | 02.07.2015 | leaf | 21.31 | 45.43 | 2.94 | 1.21 | 2.61 |
| Hydro D | 02.07.2015 | leaf | 27.00 | 45.47 | 3.48 | 1.42 | 3.00 |
| Hydro D | 12.08.2015 | leaf | 22.89 | 37.76 | 3.72 | 0.90 | 2.90 |
| Hydro D | 12.08.2015 | leaf | 25.12 | 35.41 | 3.94 | 0.81 | 3.12 |
| Hydro D | 12.08.2015 | leaf | 28.37 | 34.73 | 4.20 | 1.01 | 2.80 |
| Hydro D | 09.09.2015 | leaf | 30.13 | 37.82 | 3.61 | 0.88 | 3.04 |
| Hydro D | 09.09.2015 | leaf | 35.61 | 36.34 | 3.84 | 0.76 | 2.84 |
| Hydro D | 09.09.2015 | leaf | 14.33 | 24.19 | 2.16 | 0.53 | 2.07 |
| Hydro D | 28.05.2015 | tomato | 1.83 | 50.79 | 1.35 | 0.36 | 4.35 |
| Hydro D | 28.05.2015 | tomato | 1.86 | 40.31 | 0.97 | 0.35 | 3.20 |
| Hydro D | 28.05.2015 | tomato | 1.49 | 45.76 | 1.32 | 0.38 | 3.63 |
| Hydro D | 02.07.2015 | tomato | 1.38 | 31.87 | 1.12 | 0.45 | 2.51 |
| Hydro D | 02.07.2015 | tomato | 1.37 | 36.89 | 1.29 | 0.49 | 2.99 |
| Hydro D | 02.07.2015 | tomato | 1.19 | 39.45 | 1.38 | 0.51 | 3.76 |
| Hydro D | 12.08.2015 | tomato | 0.72 | 42.87 | 1.34 | 0.46 | 2.52 |
| Hydro D | 12.08.2015 | tomato | 1.53 | 37.29 | 1.20 | 0.21 | 3.99 |
| Hydro D | 12.08.2015 | tomato | 0.91 | 41.25 | 1.27 | 0.42 | 2.58 |
| Hydro D | 09.09.2015 | tomato | 0.96 | 41.10 | 1.42 | 0.53 | 3.33 |
| Hydro D | 09.09.2015 | tomato | 0.91 | 44.43 | 1.54 | 0.56 | 2.76 |
| Hydro D | 09.09.2015 | tomato | 1.81 | 38.95 | 1.34 | 0.23 | 4.05 |
| RAS A-C-D | 09.09.2015 | fish | 30.74 | 1.43 | 2.05 | 0.66 | 17.18 |
| RAS A-C-D | 09.09.2015 | fish | 31.05 | 1.42 | 2.05 | 0.66 | 17.53 |
| RAS A-C-D | 09.09.2015 | fish | 32.42 | 1.51 | 2.13 | 0.67 | 17.78 |
| RAS A-C-D | 09.09.2015 | fish | 32.61 | 1.50 | 2.15 | 0.69 | 18.27 |
| RAS C-D | 09.09.2015 | sludge | 18.96 | 8.21 | 0.72 | 3.56 | 12.25 |
| RAS C-D | 09.09.2015 | sludge | 13.29 | 8.23 | 0.65 | 3.45 | 9.63 |
| RAS C-D | 09.09.2015 | sludge | 4.98 | 8.43 | 0.53 | 3.38 | 5.60 |
| RAS C-D | 09.09.2015 | sludge | 10.40 | 8.29 | 0.60 | 3.42 | 8.22 |
