# Supplementary material for: Decoupled systems on trial: Eliminating bottlenecks to improve aquaponic processes
Source: PLoS One. 2017 Sep 28;12(9):e0183056. doi: 10.1371/journal.pone.0183056 (PMC5619720; doi:10.1371/journal.pone.0183056)
Supplement: S4 Table — Additionally, data for fish and sludge are presented. (DOCX) [file pone.0183056.s004.docx]

S4 Table: Elemental analysis (C/N) of plant leaves and tomatoes harvested from the hydroponic unit of the coupled (Hydro C) and the decoupled (Hydro D) aquaponic system after 30 d, 63 d, 94 d, 122 d and 154 d. Additionally, data for fish and sludge are presented.

| **experimental period / date** | **compartment** | **sample name** | **N**  **[%]** | **C**  **[%]** | **C/N** |
| --- | --- | --- | --- | --- | --- |
| 07.05-08.06.15 | leaf | C1 | 3.18 | 36.5 | 11.5 |
| 07.05-08.06.15 | leaf | C1 | 3.16 | 36.5 | 11.6 |
| 07.05-08.06.15 | leaf | C2 | 3.44 | 36.6 | 10.7 |
| 07.05-08.06.15 | leaf | C2 | 3.46 | 36.6 | 10.6 |
| 07.05-08.06.15 | leaf | C3 | 3.45 | 36.7 | 10.7 |
| 07.05-08.06.15 | leaf | C3 | 3.45 | 36.7 | 10.6 |
| 09.06-09.07.15 | leaf | C7 | 2.94 | 38.0 | 12.9 |
| 09.06-09.07.15 | leaf | C7 | 2.95 | 37.9 | 12.9 |
| 09.06-09.07.15 | leaf | C8 | 2.90 | 37.4 | 12.9 |
| 09.06-09.07.15 | leaf | C8 | 2.92 | 37.4 | 12.8 |
| 09.06-09.07.15 | leaf | C9 | 3.27 | 37.0 | 11.3 |
| 09.06-09.07.15 | leaf | C9 | 3.28 | 37.0 | 11.3 |
| 10.07.-06.08.15 | leaf | C13 | 3.44 | 38.4 | 11.2 |
| 10.07.-06.08.15 | leaf | C13 | 3.46 | 38.5 | 11.1 |
| 10.07.-06.08.15 | leaf | C14 | 2.93 | 38.1 | 13.0 |
| 10.07.-06.08.15 | leaf | C14 | 2.95 | 38.0 | 12.9 |
| 10.07.-06.08.15 | leaf | C15 | 3.27 | 38.0 | 11.6 |
| 10.07.-06.08.15 | leaf | C15 | 3.27 | 37.9 | 11.6 |
| 07.08.-07.09.15 | leaf | C19 | 2.47 | 36.7 | 14.9 |
| 07.08.-07.09.15 | leaf | C19 | 2.46 | 36.6 | 14.9 |
| 07.08.-07.09.15 | leaf | C20 | 2.46 | 37.3 | 15.1 |
| 07.08.-07.09.15 | leaf | C20 | 2.46 | 37.2 | 15.1 |
| 07.08.-07.09.15 | leaf | C21 | 2.97 | 37.3 | 12.6 |
| 07.08.-07.09.15 | leaf | C21 | 2.96 | 37.3 | 12.6 |
| 07.05-08.06.15 | tomato | C4 | 2.24 | 39.1 | 17.4 |
| 07.05-08.06.15 | tomato | C4 | 2.22 | 39.0 | 17.6 |
| 07.05-08.06.15 | tomato | C5 | 1.97 | 38.4 | 19.5 |
| 07.05-08.06.15 | tomato | C5 | 1.97 | 38.4 | 19.5 |
| 07.05-08.06.15 | tomato | C6 | 1.93 | 39.1 | 20.2 |
| 07.05-08.06.15 | tomato | C6 | 1.93 | 39.1 | 20.2 |
| 09.06-09.07.15 | tomato | C10 | 1.68 | 39.8 | 23.7 |
| 09.06-09.07.15 | tomato | C10 | 1.67 | 39.8 | 23.8 |
| 09.06-09.07.15 | tomato | C11 | 1.43 | 40.2 | 28.1 |
| 09.06-09.07.15 | tomato | C11 | 1.42 | 39.9 | 28.1 |
| 09.06-09.07.15 | tomato | C12 | 1.90 | 40.0 | 21.1 |
| 09.06-09.07.15 | tomato | C12 | 1.90 | 39.8 | 21.0 |
| 10.07.-06.08.15 | tomato | C16 | 2.22 | 39.3 | 17.8 |
| 10.07.-06.08.15 | tomato | C16 | 2.16 | 39.0 | 18.0 |
| 10.07.-06.08.15 | tomato | C17 | 1.90 | 39.0 | 20.6 |
| 10.07.-06.08.15 | tomato | C17 | 1.96 | 39.4 | 20.1 |
| 10.07.-06.08.15 | tomato | C18 | 1.86 | 39.7 | 21.3 |
| 10.07.-06.08.15 | tomato | C18 | 1.90 | 39.6 | 20.8 |
| 07.08.-07.09.15 | tomato | C22 | 1.65 | 40.4 | 24.5 |
| 07.08.-07.09.15 | tomato | C22 | 1.66 | 40.4 | 24.3 |
| 07.08.-07.09.15 | tomato | C23 | 1.82 | 39.7 | 21.8 |
| 07.08.-07.09.15 | tomato | C23 | 1.83 | 39.7 | 21.7 |
| 07.08.-07.09.15 | tomato | C24 | 2.64 | 38.9 | 14.7 |
| 07.08.-07.09.15 | tomato | C24 | 2.63 | 38.9 | 14.8 |
| 07.05-08.06.15 | leaf | D1 | 3.98 | 38.3 | 9.6 |
| 07.05-08.06.15 | leaf | D1 | 3.97 | 38.2 | 9.6 |
| 07.05-08.06.15 | leaf | D2 | 3.78 | 39.5 | 10.4 |
| 07.05-08.06.15 | leaf | D2 | 3.82 | 39.7 | 10.4 |
| 07.05-08.06.15 | leaf | D3 | 3.79 | 38.2 | 10.1 |
| 07.05-08.06.15 | leaf | D3 | 3.77 | 38.2 | 10.1 |
| 09.06-09.07.15 | leaf | D7 | 3.13 | 39.2 | 12.5 |
| 09.06-09.07.15 | leaf | D7 | 3.11 | 39.2 | 12.6 |
| 09.06-09.07.15 | leaf | D8 | 3.17 | 39.6 | 12.5 |
| 09.06-09.07.15 | leaf | D8 | 3.14 | 39.5 | 12.6 |
| 09.06-09.07.15 | leaf | D9 | 3.29 | 38.5 | 11.7 |
| 09.06-09.07.15 | leaf | D9 | 3.27 | 38.5 | 11.8 |
| 10.07.-06.08.15 | leaf | D13 | 3.80 | 39.7 | 10.4 |
| 10.07.-06.08.15 | leaf | D13 | 3.79 | 39.6 | 10.5 |
| 10.07.-06.08.15 | leaf | D14 | 3.88 | 39.3 | 10.1 |
| 10.07.-06.08.15 | leaf | D14 | 3.88 | 39.3 | 10.1 |
| 10.07.-06.08.15 | leaf | D15 | 3.65 | 38.4 | 10.5 |
| 10.07.-06.08.15 | leaf | D15 | 3.66 | 38.4 | 10.5 |
| 07.08.-07.09.15 | leaf | D19 | 3.47 | 38.1 | 11.0 |
| 07.08.-07.09.15 | leaf | D19 | 3.56 | 39.0 | 10.9 |
| 07.08.-07.09.15 | leaf | D20 | 2.45 | 40.1 | 16.4 |
| 07.08.-07.09.15 | leaf | D20 | 3.22 | 37.5 | 11.7 |
| 07.08.-07.09.15 | leaf | D21 | 3.37 | 39.5 | 11.7 |
| 07.08.-07.09.15 | leaf | D21 | 3.40 | 39.6 | 11.6 |
| 07.05-08.06.15 | tomato | D4 | 2.39 | 39.1 | 16.3 |
| 07.05-08.06.15 | tomato | D4 | 2.39 | 39.0 | 16.3 |
| 07.05-08.06.15 | tomato | D5 | 1.58 | 40.0 | 25.3 |
| 07.05-08.06.15 | tomato | D5 | 1.60 | 40.0 | 25.0 |
| 07.05-08.06.15 | tomato | D6 | 2.26 | 39.4 | 17.4 |
| 07.05-08.06.15 | tomato | D6 | 2.26 | 39.5 | 17.5 |
| 09.06-09.07.15 | tomato | D10 | 1.67 | 38.6 | 23.1 |
| 09.06-09.07.15 | tomato | D10 | 1.80 | 39.3 | 21.8 |
| 09.06-09.07.15 | tomato | D11 | 2.05 | 39.6 | 19.3 |
| 09.06-09.07.15 | tomato | D11 | 2.05 | 39.6 | 19.3 |
| 09.06-09.07.15 | tomato | D12 | 2.15 | 39.7 | 18.5 |
| 09.06-09.07.15 | tomato | D12 | 2.16 | 39.7 | 18.4 |
| 10.07.-06.08.15 | tomato | D16 | 2.16 | 39.5 | 18.3 |
| 10.07.-06.08.15 | tomato | D16 | 2.15 | 39.5 | 18.4 |
| 10.07.-06.08.15 | tomato | D17 | 1.68 | 39.8 | 23.8 |
| 10.07.-06.08.15 | tomato | D17 | 1.64 | 39.6 | 24.1 |
| 10.07.-06.08.15 | tomato | D18 | 2.15 | 39.7 | 18.5 |
| 10.07.-06.08.15 | tomato | D18 | 2.17 | 39.7 | 18.3 |
| 07.08.-07.09.15 | tomato | D22 | 2.12 | 39.3 | 18.5 |
| 07.08.-07.09.15 | tomato | D22 | 2.11 | 39.2 | 18.6 |
| 07.08.-07.09.15 | tomato | D23 | 2.52 | 39.3 | 15.6 |
| 07.08.-07.09.15 | tomato | D23 | 2.54 | 39.4 | 15.5 |
| 07.08.-07.09.15 | tomato | D24 | 1.72 | 39.3 | 22.9 |
| 07.08.-07.09.15 | tomato | D24 | 1.72 | 39.3 | 22.9 |
| 09.09.2015 | fish | Pool A | 7.4 | 56.4 | 7.57 |
| 09.09.2015 | fish | Pool A | 7.5 | 58.0 | 7.76 |
| 09.09.2015 | fish | Pool C | 7.3 | 58.9 | 8.10 |
| 09.09.2015 | fish | Pool C | 7.2 | 59.3 | 8.19 |
| 09.09.2015 | fish | Pool D | 7.8 | 56.3 | 7.23 |
| 09.09.2015 | fish | Pool D | 7.4 | 50.3 | 6.76 |
| 09.09.2015 | sludge | sludge B | 4.1 | 37.7 | 9.1 |
| 09.09.2015 | sludge | sludge B | 4.0 | 37.0 | 9.3 |
| 09.09.2015 | sludge | sludge C | 4.4 | 35.3 | 8.1 |
| 09.09.2015 | sludge | sludge C | 3.9 | 36.5 | 9.3 |
