# Supplementary material for: Decoupled systems on trial: Eliminating bottlenecks to improve aquaponic processes
Source: PLoS One. 2017 Sep 28;12(9):e0183056. doi: 10.1371/journal.pone.0183056 (PMC5619720; doi:10.1371/journal.pone.0183056)
Supplement: S5 Table — (DOCX) [file pone.0183056.s005.docx]

S5 Table: Solid removal (g dry weight * L^-1^) in the fish unit of the coupled (RAS C) and the decoupled (RAS D) aquaponic system due to weekly cleaning of the clarifier (V = 1.5 m^3^) over three consecutive weeks.

| **RAS** | **week** | **sample_id** | **dry weight [g] in 10 L** | **dryweight [gL^-1^]** |
| --- | --- | --- | --- | --- |
|  |  |  |  |  |
| D | 1 | 1 | 18.5277 | 1.9 |
| D | 1 | 2 | 20.2294 | 2.0 |
| D | 1 | 3 | 23.1401 | 2.3 |
| D | 1 | 4 | 20.6678 | 2.1 |
| D | 1 | 5 | 19.4015 | 1.9 |
| D | 2 | 6 | 19.8060 | 2.0 |
| D | 2 | 7 | 19.9613 | 2.0 |
| D | 2 | 8 | 20.1679 | 2.0 |
| D | 2 | 9 | 19.3804 | 1.9 |
| D | 2 | 10 | 19.1364 | 1.9 |
| D | 3 | 11 | 18.3822 | 1.8 |
| D | 3 | 12 | 18.3329 | 1.8 |
| D | 3 | 13 | 18.5595 | 1.9 |
| D | 3 | 14 | 18.2229 | 1.8 |
| D | 3 | 15 | 19.2992 | 1.9 |
| C | 1 | 16 | 19.8735 | 2.0 |
| C | 1 | 17 | 21.9586 | 2.2 |
| C | 1 | 18 | 17.8345 | 1.8 |
| C | 1 | 19 | 18.6593 | 1.9 |
| C | 1 | 20 | 17.7682 | 1.8 |
| C | 2 | 21 | 17.6191 | 1.8 |
| C | 2 | 22 | 19.3564 | 1.9 |
| C | 2 | 23 | 18.5932 | 1.9 |
| C | 2 | 24 | 16.7612 | 1.7 |
| C | 2 | 25 | 16.9435 | 1.7 |
| C | 3 | 26 | 17.5891 | 1.8 |
| C | 3 | 27 | 18.1678 | 1.8 |
| C | 3 | 28 | 19.1056 | 1.9 |
| C | 3 | 29 | 18.3526 | 1.8 |
| C | 3 | 30 | 17.9687 | 1.8 |
