# Supplementary material for: Decoupled systems on trial: Eliminating bottlenecks to improve aquaponic processes
Source: PLoS One. 2017 Sep 28;12(9):e0183056. doi: 10.1371/journal.pone.0183056 (PMC5619720; doi:10.1371/journal.pone.0183056)
Supplement: S6 Table — SD–standard deviation. (DOC) [file pone.0183056.s006.doc]

**S6 Table: Total suspended solids (TSS, g dry weight/L rearing water) in the fish unit of the aquaculture reference (RAS A), the coupled (RAS C) and decoupled (RAS D) aquaponic system after 30 d (1 month), 94 d (3 month) and 154 d (5 month). SD – standard deviation**

| **RAS** | **month** | **TSS in [mg] in 1 L** | **mean** | **SD** |
| --- | --- | --- | --- | --- |
| A | April | 0.9 | 0.9 | 0.1 |
| A | April | 0.75 |
| A | April | 0.96 |
| B | April | 1.04 | 1.0 | 0.1 |
| B | April | 0.93 |
| B | April | 0.99 |
| C | April | 1.1 | 1.0 | 0.2 |
| C | April | 0.8 |
| C | April | 1.15 |
| A | June | 2.1 | 2.2 | 0.3 |
| A | June | 2.5 |
| A | June | 1.9 |
| B | June | 5.8 | 5.0 | 0.7 |
| B | June | 4.7 |
| B | June | 4.5 |
| C | June | 3.8 | 3.4 | 0.6 |
| C | June | 3.7 |
| C | June | 2.8 |
| A | September | 3.8 | 3.6 | 0.2 |
| A | September | 3.5 |
| A | September | 3.6 |
| B | September | 7.5 | 6.9 | 0.5 |
| B | September | 6.5 |
| B | September | 6.7 |
| C | September | 5.9 | 5.9 | 0.4 |
| C | September | 6.2 |
| C | September | 5.5 |
